# AI-Enhanced Understanding of Retention Interactions in Supercritical Fluid Chromatography: Neural Network Insights into Retention on Selected Non-Polar Stationary Phases

**DOI:** 10.1021/acs.analchem.4c05176

**Published:** 2025-01-21

**Authors:** Kateřina Plachká, Veronika Pilařová, Tat́ána Gazárková, František Švec, Jean-Christophe Garrigues, Lucie Nováková

**Affiliations:** † Department of Analytical Chemistry, Faculty of Pharmacy in Hradec Králové, Charles University, 500 05 Hradec Králové, Czechia; ‡ SOFTMAT (IMRCP) Laboratory, SMODD Team, CNRS, Toulouse III Paul Sabatier University, 31400 Toulouse, France

## Abstract

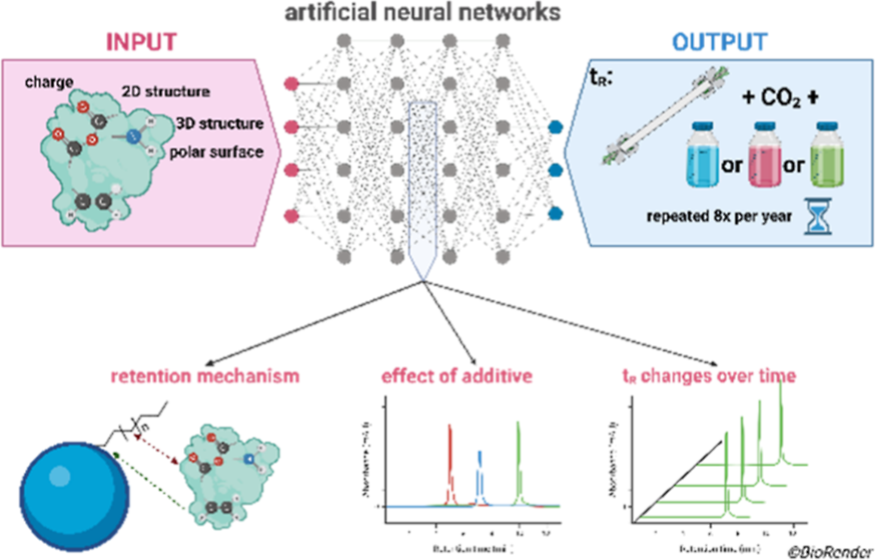

The retention behavior in supercritical fluid chromatography (SFC) remains a complex and poorly understood phenomenon despite the development of various models to explain retention mechanisms. This study aims to deepen the understanding of retention by investigating three distinct stationary phases: high-strength silica octadecyl (HSS C18 SB), charged surface hybrid pentafluorophenyl (CSH PFP), and porous graphitic carbon (PGC) as a nonsilica-based phase. Three mobile phase compositions, i.e., CO_2_/methanol, CO_2_/methanol +10 mmol/L NH_3_, and CO_2_/methanol +2% H_2_O, were investigated using an extensive set of analytes characterized by over 200 molecular descriptors. Artificial neural networks were employed to analyze the influence of these descriptors on retention behavior, revealing the most significant molecular features that increase or decrease retention on each column with the three different mobile phases. This complex evaluation of the large set of experimental data enabled to link specific analyte properties to retention interactions in SFC, including the interaction of analytes with partial positive charge with silanol groups on the HSS C18 SB column when using methanol + H_2_O as the organic modifier. The flexibility of the alkyl chain in the HSS C18 SB column is also affected by the composition of the organic modifier, which alters retention mechanisms, especially when NH_3_ is used as an additive. This highlights the critical role of the mobile phase composition in modulating the behavior of nonpolar stationary phases. Completely different interaction mechanisms were observed for the PGC column when comparing methanol with and without additives, suggesting possible modifications to the planar structure and surface polarizability of the PGC phase. Statistical evaluation of data collected over a year of column usage demonstrated distinct long-term retention stability trends. The HSS C18 SB column exhibited the greatest stability with methanol + H_2_O, whereas significant retention decreases were observed with methanol + NH_3_ modifier, particularly for CSH PFP and, unexpectedly, also for PGC. These findings provide crucial insights into the long-term retention behavior and aging of SFC columns, with practical implications for optimizing SFC conditions and improving column lifetime.

Current supercritical fluid chromatography (SFC) is an important research tool in many fields due to its unique mobile phase properties, the possibility of employing any stationary phase, and tunable selectivity.^1−6^ Despite its undeniable advantages, SFC suffers from several negative aspects related to long-term retention time stability.^4,7−9^ In this paper, we present a detailed investigation of the retention behavior of two columns with non-endcapped silica support, namely charged surface hybrid silica fluorophenyl (CSH PFP)^10^ and high-strength silica C18 (HSS C18 SB), where SB stands for selectiviy for bases, and porous graphitic carbon (PGC) as nonsilica stationary phase, which should be free of silyl-ether formation (SEF). The C18 stationary phase (Supporting Information Figure S1a) has been used in >25% of published SFC applications, making it the most widely used stationary phase together with silica.^1^ Multiple C18 phases with different end-capping and linkers from different manufacturers are used. Therefore, based on linear solvation energy relationships (LSER), they cluster into two groups.^11^ The first group of nonpolar alkyl phases is more hydrophobic with behavior mostly identical to the C18 used in reversed-phase liquid chromatography. In LSER, they are characterized by strong dispersive (term *e*) and electrostatic (*d*^–^, *d*^+^) interactions, probably related to free silanols. No polar interactions, e.g., H-bonding, are observed (*a*, *b*, and *s*). The second group includes alkyl phases with some polar properties, such as non-endcapped C18 phases (e.g., HSS C18 SB) and polar embedded C18 phases, e.g., BEH Shield RP18, all with more interactions, including strong dispersive interactions complemented by strong polar interactions. HSS C18 SB has pronounced terms *a* and *b*, indicating the H-bonding of acids by free silanols and interactions with basic compounds. HSS C18 SB has a polymeric character due to the use of trifunctional silane for stationary phase synthesis,^12^ resulting in the presence of pendant silanols within the bulk of the bonded chain, which are available for the H-bonding with polar compounds.^11−13^ In addition, the *d*^–^ term results from the repulsion of anions from silanol groups, and the positive *d*^+^ term signifies the attraction of cations. HSS C18 SB surface is characterized by large accessible polar sites, allowing shape recognition as confirmed by the carotenoid test.^13,14^ CSH PFP is considered an alternative nonpolar stationary phase, where the charged hybrid silica is substituted by the stable ring characterized by low polarizability and high electron density. Due to its solvophobicity and fluorophilicity, it provides different selectivity for molecules with rigid skeletons. Linear compounds are weakly retained. The PFP ligand is responsible for π–π and dispersive interactions (*e* and *s* terms in LSER, Supporting Information Figure S1b), while dipole–dipole interactions are reduced (negative *v* term).^15^ Fluorine heteroatoms can attract cations and basic compounds due to the electron density (*b* and *d*^+^ terms). The interactions with anions and acids via H-bonding are affected by the positively charged surface (terms *a* and *d*^–^). Ionized silanols may be responsible for the retention of oxygen-containing molecules, probably due to the electrostatic interactions resulting from the ion exchange.^11,13,16,17^

The PGC is the most studied stationary phase in SFC among nonsilica based stationary phases.^1^ It consists of flat sheets of carbon atoms arranged hexagonally (Figure S1a). The carbon network contains overlapping hybridized orbitals (sp^2^) responsible for the electron lone pair donor–acceptor interactions.^18^ The –OH, –CHO, and –COOH groups are expected at the edge of the graphite sheet. They should represent <1% of the surface and not significantly affect the retention.^19^ The PGC retention mechanism is based on the combination of hydrophobic, dispersive, dipole–dipole, and electron lone pair donor–acceptor interactions. The hydrophobicity is a result of the absence of silanols. The polarity of the PGC can be easily increased by the adsorption of methanol (MeOH), which affects the *v* and *e* terms in the LSER, i.e., dipole–dipole and dispersive interactions. The MeOH adsorption also reduces the ability of charge transfer interactions as increases the density of electrons. Thus, the selectivity is mainly affected by (i) planar surface bringing high steric selectivity, (ii) dispersive interactions providing higher methylene selectivity compared to C18, and (iii) hydroxyl selectivity affected by H-bond accepting ability of the stationary phase. PGC has excellent thermal and pH stability, but a long equilibration time is recommended.^1,20−22^

To quantitatively determine the analyte features contributing to the retention on HSS C18, CSH PFP, and PGC and to increase the knowledge in SEF, we conducted a quantitative structure–retention relationship study using artificial neural networks (ANN) to identify the interaction mechanisms between 107 analytes and the stationary phases. We tested three different mobile phase compositions, including CO_2_ with (i) pure MeOH, (ii) MeOH + 2% H_2_O, and (iii) MeOH + 10 mmol/L NH_3_ to cover different pH^23^ and to observe the effect of the additive on SEF.

## Experimental Section

### Chemicals

LC/MS grade MeOH, acetonitrile (ACN), 2-propanol, and water were provided by VWR International (Prague, Czechia). Ammonia 4 mol/L solution in MeOH for LC/MS was purchased from Sigma-Aldrich (Steinheim, Germany) and pressurized liquid CO_2_ 4.5 grade (99.9995%) from Messer (Prague, Czechia). Most of the 107 reference standards listed in Supporting Information Table S1 were obtained from Sigma-Aldrich (Prague, Czechia) except for several standards kindly donated by Zentiva, k.s. (Prague, Czechia).

### Analytical Conditions

**Figure 1:**
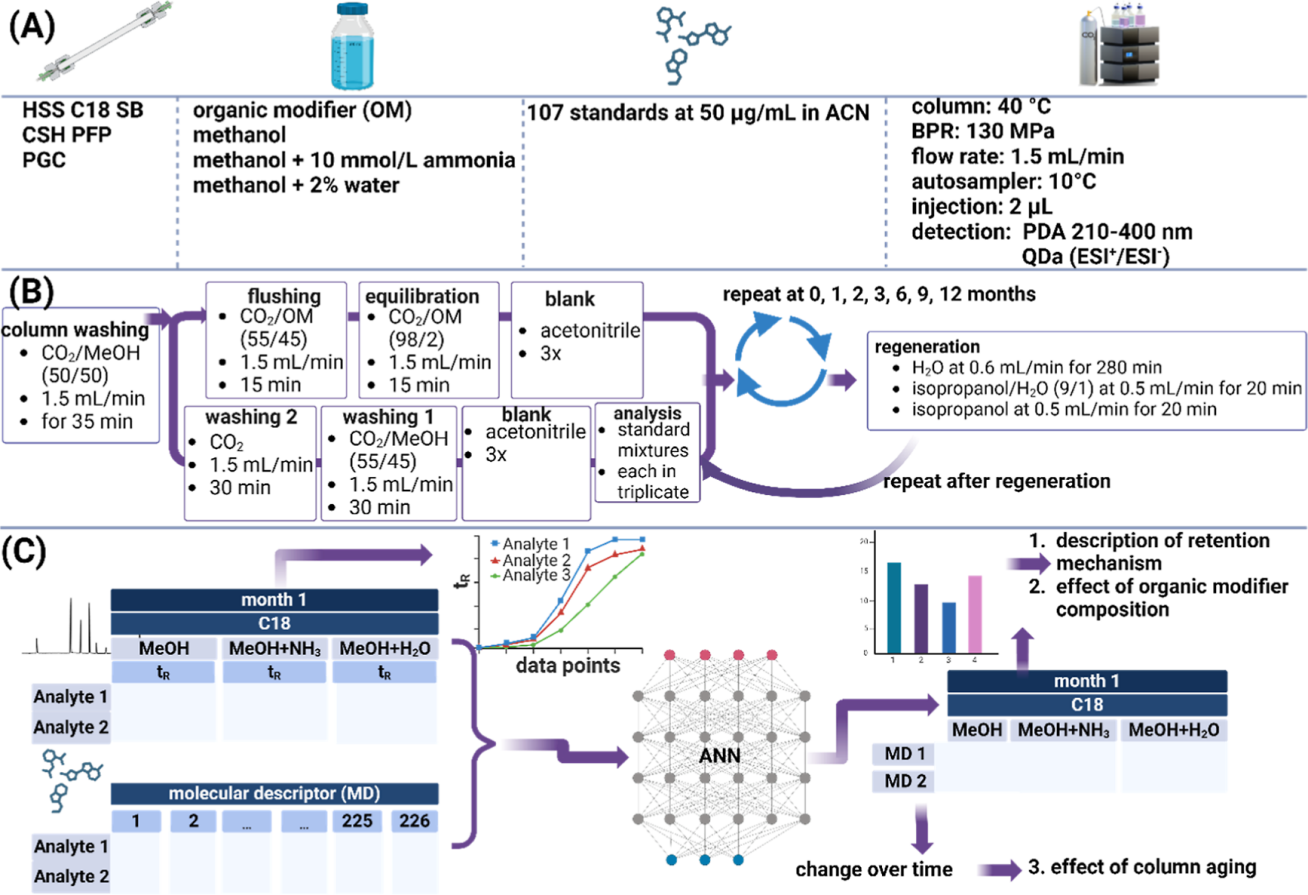
Workflow of the study, including the used analytical conditions. The figure was created using BioRender.com.

The analytical conditions and protocols followed those described previously.^5,6^ Briefly, the standard stock solutions of all reference standards were prepared in MeOH, diluted by ACN, and divided into working mixtures with a final concentration of 50 μg/mL. The experiments were carried out on an Acquity UPC^2^ supercritical fluid chromatography system (Waters, USA) configured with a binary pump, an autosampler, a column thermostat, a back pressure regulator (BPR), a PDA detector, and a single quadrupole MS detector (QDa, Waters) with an SFC-MS dedicated pre-BPR splitter with an additional isocratic pump (Waters). The system was controlled by Empower 3 software. The conditions are listed in Figure 1A. A generic gradient method with a mobile phase composed of CO_2_ (A) and organic modifier (B) was used: 2% B for 1 min, 0–45% B in 1.0–5.0 min, followed by 1 min isocratic step at 45% B and 1.5 min equilibration at initial conditions. The gradient program extended for 14 min of isocratic elution at 45% of organic modifier was used for experiments on the PGC columns to increase the number of eluting compounds. The BPR pressure was adjusted prior to each sequence to ensure that the system pressure remained within ±0.07 MPa (10 psi) throughout the study. QDa detector was used to confirm the identity of each analyte. MeOH + 10 mmol/L NH_3_ was used as the makeup solvent at 0.3 mL/min. The study workflow is summarized in Figure 1.^6^ The regeneration protocol (Figure 1B) was carried out according to the information from previous research and the guidelines from Waters Column Care & Manual.^6,9,24^ Acquity UPLC (Waters) controlled by Empower 3 software was used for the column regeneration. Three stationary phases were tested: high-strength silica modified with C18 (Viridis HSS C18 SB, Waters, C18), charged hybrid silica with pentafluorophenyl modification (Viridis CSH PFP, Waters, CSH PFP), and porous graphitic carbon (Hypercarb, ThermoScientific, USA, PGC). All columns had dimensions of 100 × 3.0 mm and were packed with 1.8, 1.7, and 3.0 μm particles, respectively.

### Data Processing and Evaluation

An overview of the data processing protocol is shown in Figure 1C. Raw data was evaluated using Empower 3 software, and the percentage change in *t*_R_ over time was calculated for each analyte, column, and organic modifier using Excel. The 3D structures of the analytes were optimized by semiempirical AM1 quantum mechanical calculations (MOPAC application of Chem 3D Pro version 14.0 software CambridgeSoft) with a root-mean-square gradient of 0.100 to minimize their energy. The optimized structures were used to calculate 2D and 3D molecular descriptors using the CDK Descriptor Calculator (v.1.4.8). The 226 calculated molecular descriptors are listed in the Supporting Information Table S2 and grouped in Supporting Information Table S3. All the molecular descriptors and *k*′ were normalized by dividing by the maximum value.

ANN were created using the neural network simulator in MATLAB R2023a with the deep learning toolbox V.23.2 (The MathWorks, Inc., USA) with a sigmoid activation function and a 500-cycle backpropagation learning algorithm. The input layer was connected to the 226 molecular descriptors and an output layer to the retention factors (*k*′). The weights assigned to each input neuron were extracted, and the key parameters with the highest absolute weights >1.5 were examined.^25^ The relevance of regression learning was assessed by retaining only ANN systems that showed an RMSE (Root Mean Squared Error) of less than 0.5 between the target training values and the calculated values after 500 learning cycles. The standard deviation (SD) of the molecular descriptor weights at different time points was calculated and used to explain the observed changes in *t*_R_.

## Results and Discussion

Our study was carried out using (i) separate columns for each of the organic modifiers to avoid switching of additives within one column, (ii) a long equilibration time using a higher proportion of organic modifiers with additives to ensure repeatable coverage of the stationary phase surface, (iii) flushing of the column after its use with large volumes of organic solvent and neat CO_2_ (>30 column volumes), and (iv) storing of the columns in neat CO_2_ to prevent SEF.

The set of analytes included mostly pharmaceutical and biologically active compounds representing compounds, most commonly analyzed by SFC. The diversity in physicochemical properties then enables to describe various interaction mechanisms between analytes and the stationary phase. The selection of organic modifiers in this study was based on the need to balance feasibility and scientific relevance. Indeed, these three organic modifiers cover the most commonly used SFC mobile phases. Methanol was selected to provide a baseline free from additive-induced interactions, while methanol with 2% water was included for its known benefits in stabilizing retention times.^2^ Furthermore, the apparent pH of a CO_2_/MeOH/H_2_O mobile phase is acidic^23^ mimicking the acidic conditions of commonly used additives, su formic acid. Methanol with ammonia was selected to explore its direct effects on retention, avoiding the additional complexity introduced by ionic interactions from ammonium salts.

**Figure 2:**
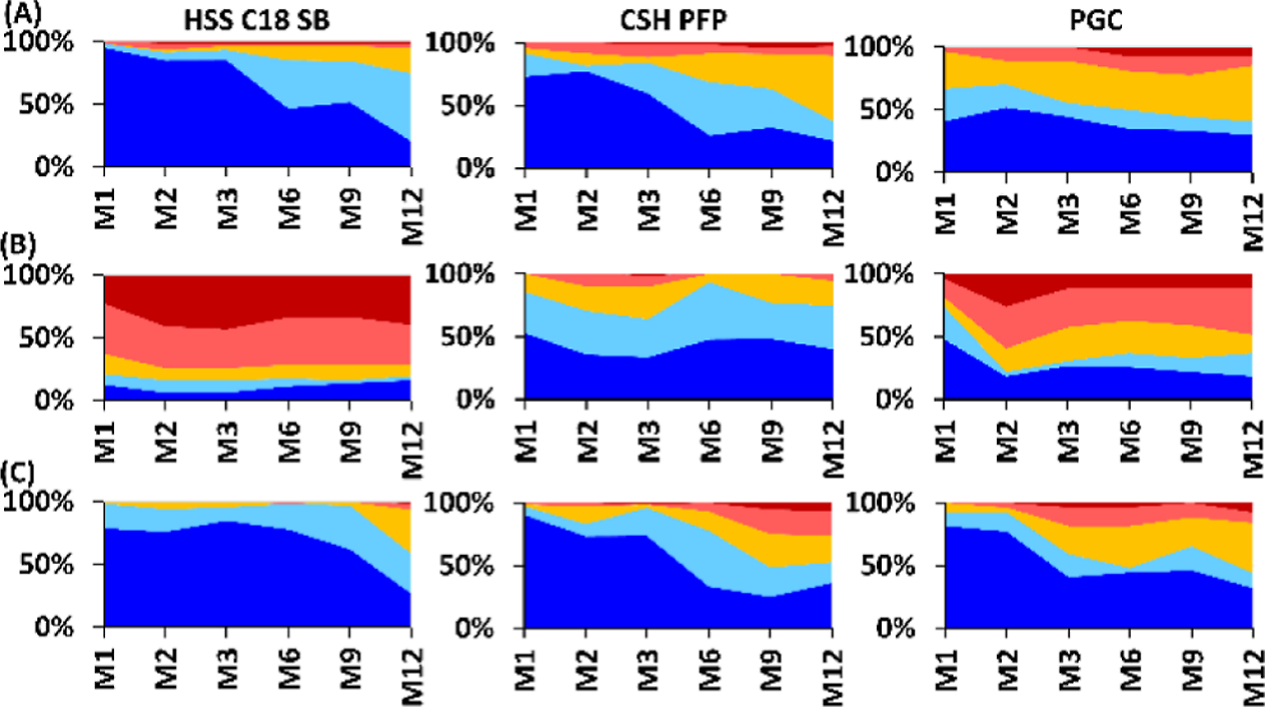
Retention time shifts on HSS C18 SB, CSH PFP, and PGC stationary phases within one year using (A) MeOH, (B) MeOH + 10 mmol/L NH_3_, and (C) MeOH + 2% H_2_O as organic modifiers, expressed as %-difference: <0.5% (dark blue), 0.5–1.0% (light blue), 1.0–2.0% (yellow), 2.0–5.0% (light red), and >5.0% (dark red), M = month.

The *t*_R_ were relatively stable over one year on the C18 column using MeOH and MeOH + H_2_O, with >80% of compounds having *t*_R_ within ±1% of the first injection even after 9 months (M). On the contrary, the differences between *t*_R_ were quite severe when using MeOH + NH_3_. However, there was an abrupt change between the first injection and M1, followed by stable *t*_R_. This suggests that the coverage of the stationary phase surface by the NH_3_ was insufficient and even longer equilibration was needed to achieve a repeatable retention similar to that of the silica stationary phase discussed previously.^6^ The opposite behavior was observed on the CSH PFP column. Higher *t*_R_ instability was observed on CSH PFP using MeOH and MeOH + H_2_O, in contrast to MeOH + NH_3_ (Figure 2A). Unstable *t*_R_ were also observed on the PGC column. *T*_R_ shifts caused by SEF should not be observed on PGC, suggesting other mechanisms significantly affected the stationary phase surface and changed the retention behavior over time. The variability of *t*_R_ on PGC columns is generally attributed to high susceptibility to contamination, adsorption of mobile phase, and possible oxidation/reduction of the surface.^22^ As high purity solvents were used and the cross-contamination by mobile phase components was avoided by using three different PGC columns, the *t*_R_ shifts are probably related to occurring redox reactions on stationary phase surface.

To determine which analyte properties play a critical role in retention and *t*_R_ shifts over time, ANN were used to identify by what weight a given molecular descriptor affects the observed *k*′ (Supporting Information Figures S2–S4). Only 28 analytes in our tested set eluted from the PGC column due to their strong retention, which significantly reduced the statistical significance of the data evaluation. However, the PCA analysis of molecular descriptors of analytes eluting on all three stationary phases showed that these analytes cover the same molecular space without significant clustering or separation between the groups (Supporting Information Figure S5). Furthermore, the comparison of retention factors showed that the *t*_R_ variation was consistent using all tested conditions (Supporting Information Figure S6).

Only C18 and CSH PFP were compared in the first step. Although both columns are nonpolar stationary phases, their retention behavior differed significantly allowing retention/elution of analytes with different properties. Thus, the retention behavior was described using both, a set of analytes eluting on both stationary phases and two separate sets of analytes specific to each column to avoid misinterpretation of the results.

50 analytes eluted from both C18 and CSH PFP using all three organic modifiers. This set of analytes was used for the first ANN evaluation, allowing a direct comparison of the retention behavior (Supporting Information Figure S7). The molecular descriptors with the most significant differences in weights, i.e., their effect on retention, between C18 and CSH PFP are shown in Supporting Information Figure S7. The largest difference was observed for descriptors, which expresses the distance edge between oxygens. High values of MDEO-22, i.e., the distance edge between secondary oxygens, increased the retention of CSH PFP, whereas high values of MDEO-22 and MDEO-11, i.e., the distance edge between the primary oxygens, decreased the retention on CSH PFP. That shows that the type of bonding of the oxygens in the molecule is crucial for its retention behavior. The bond between carbon and primary oxygen, e.g., in alcohols and aldehydes, is highly polarizable, resulting in a positive partial charge on the carbon as opposed to a negative partial charge on oxygen. On the contrary, the bonds in secondary oxygen groups, i.e., ethers, have a small dipole moment. Compounds with a high number of primary oxygens, i.e., low values of distance edge between them, and a low number of secondary oxygens, i.e., high values of distance edge between them, were more strongly retained on CSH PFP. The distance edge between primary/secondary and secondary/tertiary carbons (MDEC-12 and MDEC-23) significantly increased the retention on C18 and had almost no effect on the retention on CSH PFP. A similar effect was observed also for the LipinskiFailure, a 2D descriptor based on 5 rules related to the solubility and pharmacokinetic properties.^26^ As LipinskiFailure calculates how much the compounds is outside of Lipinski rules, it cannot be directly correlated to retention interactions. However, it shows what type of molecules will be more strongly retained. More detailed explanation focusing on particular properties included in Lipinski rules is provided in the discussion focusing on each stationary phase. This first comparison showed that the retention behavior on C18 was more directly affected by the dispersive interactions than CSH PFP, where the π–π interactions were more pronounced.

**Figure 3:**
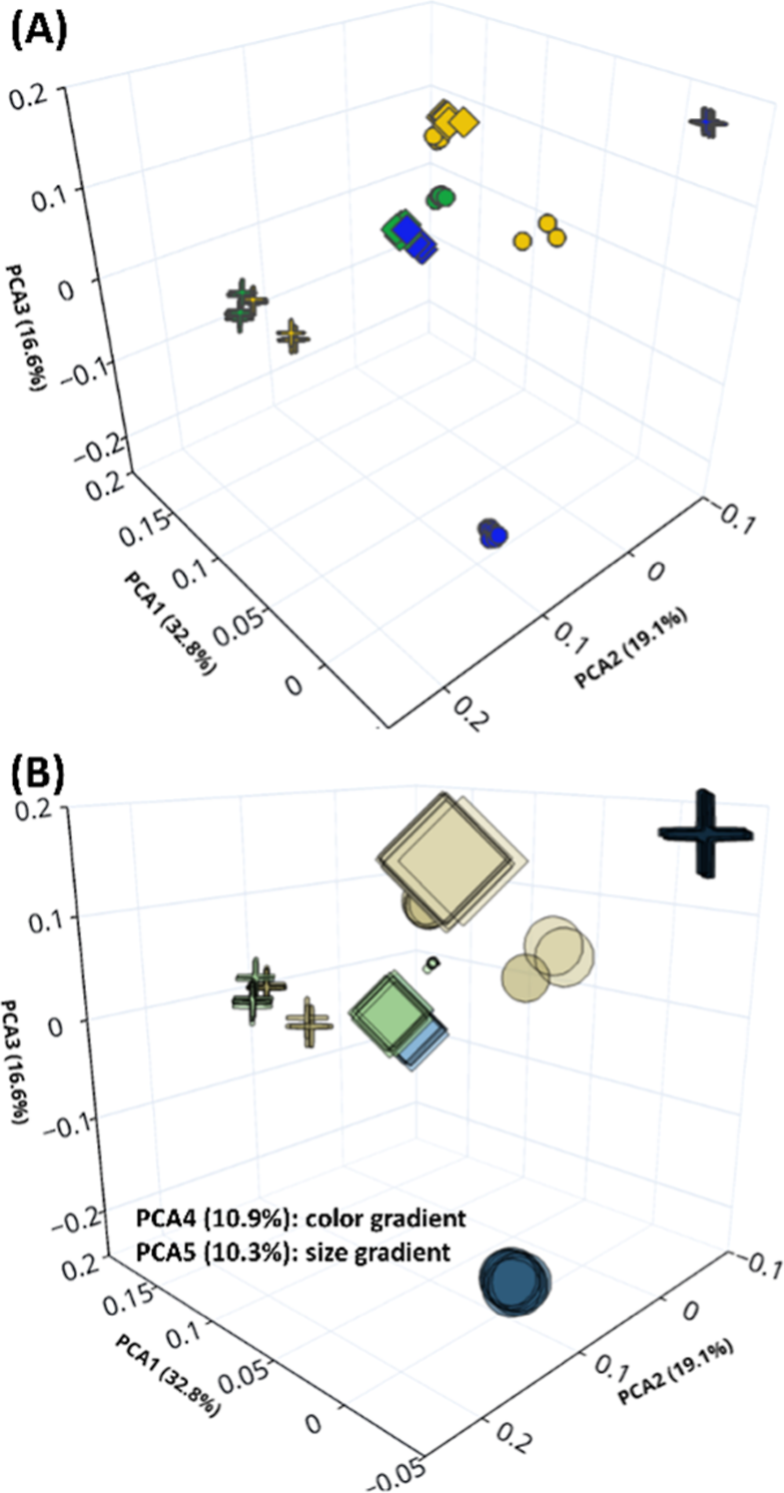
Principal component analysis using three (A) and five (B) principal components of weights assigned by ANN to molecular descriptors based on the extended set of compounds analyzed on HSS C18 SB (●), CSH PFP (◆), and PGC (+) using MeOH (a-blue, b-gradient of blue), MeOH + 2% H_2_O (a-green, b-gradient of green), and MeOH + 10 mmol/L NH_3_ (a-yellow, b-gradient of yellow to grey). Multiple markers correspond to different data points.

Principal component analysis of the ANN-assigned weights of the different molecular descriptors showed that the retention interactions changed significantly with the change in organic modifier on all three columns (Figure 3). Similar retention interactions were observed on CSH PFP using MeOH and MeOH + H_2_O clustering closely together. In contrast, the use of MeOH + NH_3_ resulted in a completely different retention mechanism. Surprisingly, more similar interactions were observed on C18 using MeOH + H_2_O and MeOH + NH_3_ contrary to MeOH, as the conditions with additives clustered closer in Figure 3A. However, this plot represents only three principal components (PC), which account for only 68.5% of the data variance. Five principal components were required to describe 89.7% of the data, with PC 4 corresponding to the color gradient and PC 5 to the size gradient in Figure 3B. Figure 3B clearly shows that although C18/MeOH + NH_3_ and CHS PFP/MeOH + NH_3_ cluster closely in the 3D plot, they differ significantly in the third and fourth PC. The most significant difference in PGC was observed between organic modifiers with and without additives regardless of the type of additive, even in the 3D plot.

### Retention Behavior on HSS C18 SB

**Figure 4:**
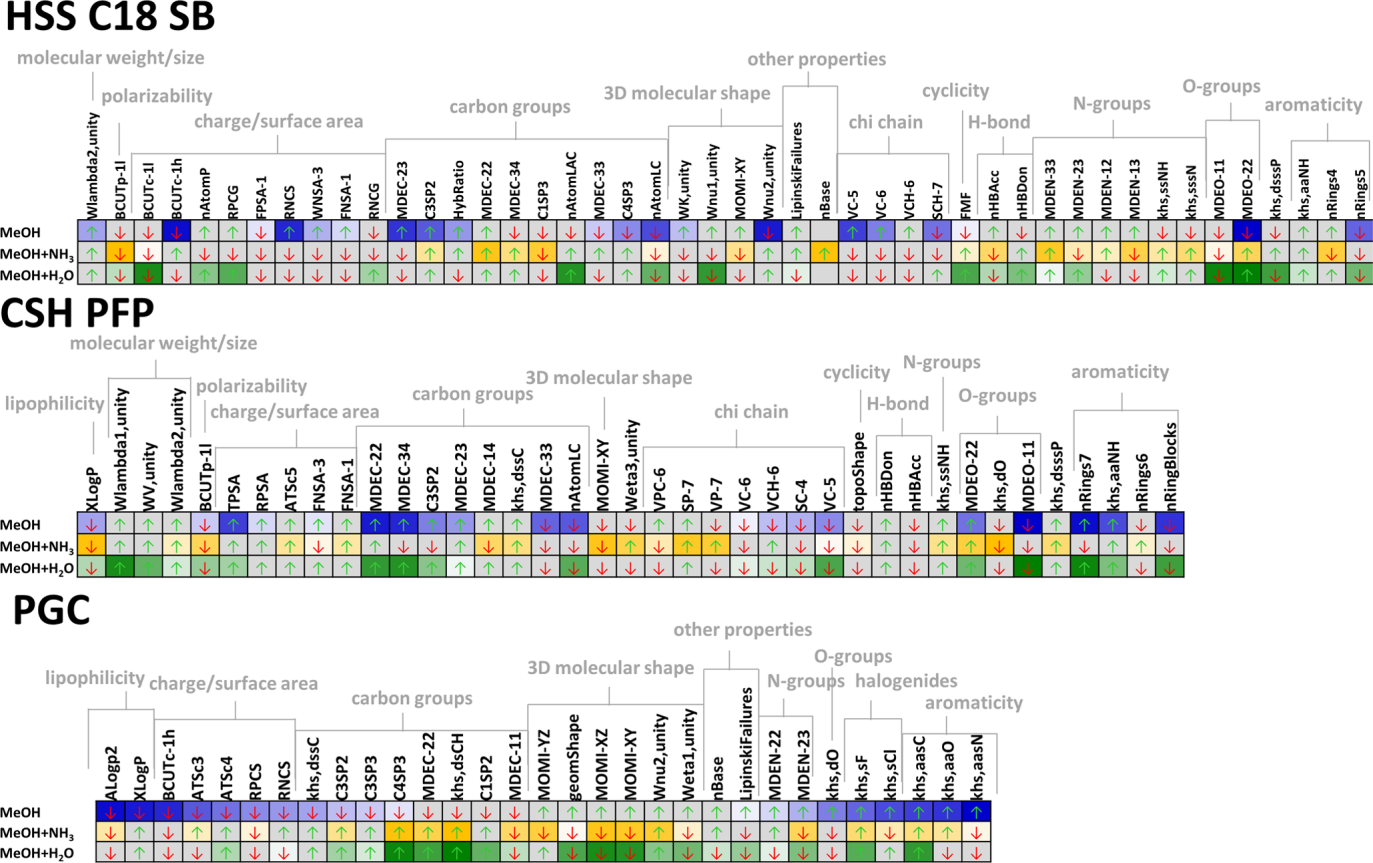
Molecular descriptors affecting the retention on the tested stationary phases by the highest weights as determined by ANN using the extended set of compounds for each organic modifier: methanol (blue), 10 mmol/L ammonia in methanol (yellow), and 2% water in methanol (green). The shade of color in the heatmaps corresponds to a ranking of the molecular descriptor for each makeup solvent composition (ranking 1 = the highest absolute ANN-assigned weight = the darkest color). ↑-increasing effect on retention, ↓-decreasing effect on retention.

Seven additional compounds were eluted from the C18 column using MeOH compared to the set of analytes for C18/CSH PFP. Most of these 57 analytes were neutral compounds. Twenty-three additional compounds were successfully analyzed using MeOH + NH_3_, most of them with strong acidic and/or basic properties (Supporting Information Table S4). Supporting Information Figure S8 shows the 20 key molecular descriptors affecting the retention on C18 separately for each tested organic modifier and considering all compounds eluting using each mobile phase (extended set). Figure 4 then compares the weights of all these selected molecular descriptors to demonstrate changes caused by different organic modifier compositions. All analytes eluting using each of the organic modifiers were used for this evaluation.

The change in organic modifier shifts the apparent pH of the mobile phase, which may affect the free silanols but should not affect the C18 functional groups. Using pure MeOH with apparent pH ≈ 5,^23^ the retention decreased with increasing (i) MDEO-22, (ii) charge expressed as BCUTc-1h, (iii) number of atoms in the longest chain (nAtomLC), (iv) carbons bonded to 4 other carbons (C4SP3, quaternary carbon), (v) distance between tertiary carbons (MDEC-33), and (vi) molecular framework (FMF). FMF describes the complexity of the molecule, quantifying the ratio of molecule part of the framework related to the entire molecular structure.^27,28^ As acyclic molecules have no framework, their FMF equals 0. On the contrary, compounds with both aromatic and nonaromatic cycles in the structure have FMF equal to 1. The negative weight assigned by ANN to FMF means that cyclic rigid compounds will be significantly less retained than acyclic. (vii) Wnu, a holistic WHIM (Weighted Holistic Invariant Molecular) descriptor related to the molecular shape^29^ decreased *t*_R_. On the other hand, Wlambda and WK, related to molecular size and atom distribution/density, respectively, increased the retention. (viii) The compounds with a predominantly negative charge on the molecule surface were more strongly retained on C18 using MeOH. High values of FNSA-1, i.e., the sum of the surface area on negative parts of the molecule/total molecular surface area, increased the retention using MeOH. This was confirmed by high weights of molecular descriptors WNSA-3 (charge weighted partial negative surface area × total molecular surface area/1000) and RNCS (relative negative charge surface area). (ix) HybRatio, calculated as sp^3^ carbon atoms divided by (sp^3^ + sp^2^ carbons), had an increasing effect on retention with decreasing HybRatio value caused by a number of sp^2^ carbons increase. However, HybRatio does not account for the type of atom to which the carbon is doubly bonded. C3SP2 descriptor counts carbons bonded to three other carbons with one double bond, thus, only sp^2^ carbons bonded to other carbons. It was assigned a positive weight by ANN, similar to HybRatio. We suppose that sp^2^ carbons doubly bonded to heteroatoms decrease the retention. The importance of the valence electrons and dispersive interactions is further confirmed by VC-5 and VC-6 descriptors, i.e., chi chain descriptors of valence clusters.

The presence of NH_3_ to the modifier results in more stable acid-base properties over the gradient program. The change of apparent pH was observed ranging from ≈3 to ≈8.^23^ In our study, the ammonia completely changed the retention behavior when compared with MeOH and MeOH + H_2_O. We hypothesize that it could be a result of an apparent pH change. (i) MDEO-22 remained one of the most influential molecular descriptors, but it increased retention. This suggests that the secondary oxygens located closely together in the structure of the analytes promoted elution. In contrast, a large value of MDEO-11 decreased retention, suggesting a higher number of primary oxygens enabled stronger interactions with the stationary phase. The main difference between primary and secondary oxygens is in the polarizability of their bonds. We assume that the polarized primary groups with a partial negative charge on the oxygen can interact with the free silanols of the stationary phase and/or methanol adsorbed on these silanols, resulting in higher retention. (ii) The high negative weight of BCUTp-1l associated with the lowest polarizability confirmed this hypothesis.

Similarly to C18/MeOH, retention using MeOH + NH_3_ decreased with increasing (iii) nAtomLC. (iv) Molecules with large distance edges between two secondary and tertiary and quaternary carbons (MDEC-22, MDEC-34) and a high number of =C< groups (C3SP2) were retained more strongly than compounds with a high number of methyl groups (C1SP3). (v) Molecules with low charge (BCUT-c1l) were less retained. (vi) This preference for the retention of charged molecules can be correlated with the positive weights assigned to the number of basic groups (nBase) and secondary and tertiary amines (khs.ssNH and khs.sssN). Indeed, nBase was the most influential molecular descriptor increasing retention using MeOH + NH_3_ but had almost no effect using the other organic modifiers. The key effect of amine groups and their position in the chemical structure was confirmed by descriptors related to the molecular distance edge between them (MDEN-12, MDEN-13, MDEN-23, and MDEN-33).

(vii) Contrary to C18/MeOH, positive weight was assigned to FMF using MeOH + NH_3_. A higher FMF value indicates that the molecule is dominated by its core framework, contrary to compounds with many substituents or side chains, which have lower FMF values. The ANN-assigned positive weight means that cyclic rigid molecules were retained more than acyclic compounds with lower values of FMF. It was shown that in reversed-phase liquid chromatography, the organic modifiers can modify the flexibility of the alkyl chains in C18 stationary phases. Specifically, MeOH increases flexibility, which affects retention mechanisms.^30^ We observed similar results in the CO_2_-based mobile phase using MeOH as an organic modifier where high flexibility of C18 chains corresponds with higher retention of acyclic, less rigid compounds. We assume that the addition of NH_3_ to the mobile phase changed this behavior resulting in less flexible C18 chains and thus enhanced retention of cyclic compounds. (ix) The negative weight of the number of H-bond acceptors (nHBAcc) shows that the surface of the stationary phase contains sites that act as H-bond acceptors, probably free silanols, resulting in repulsion between these sites and analytes with a high number of H-bond acceptors.

The 3D shape of the molecule must also be considered. (viii) Retention decreased with increasing values of MOMI-XY. The moment of inertia (MOMI) describes the 3D shape of the molecule. It is calculated based on three perpendicular axes passing through the center of mass and the mass distribution from these axes. Four types of 3D shapes can be determined: linear, symmetric top, spherical, and asymmetric top (Supporting Information Figure S9). High values of MOMI-XY correspond to oblate and asymmetric molecule indicating lower retention compared to linear compounds.

The presence of H_2_O in the organic modifier resulted in retention mechanisms more similar to MeOH + NH_3_ than to pure MeOH. (i) The increasing effect of MDEO-22 was again observed together with the decreasing effect of MDEO-11. (ii) The repulsion of compounds with H-bond acceptor groups and the increasing effect of the H-bond donor groups were observed. A closer look at Figure 4 shows that the positive effect of nHBDon was also observed using MeOH + NH_3._ However, it was not among the 10 key descriptors. (iii) In contrast to MeOH, where RNCS significantly increased retention, both RPCG and RNCG, i.e., the relative positive and negative charge, increased retention when MeOH + H_2_O was used. The use of MeOH + H_2_O resulted in an acidic mobile phase with an apparent pH close to aqueous pH ≈ 1.^23^ The HSS C18 SB stationary phase contains large accessible polar sites that allow H-bonding with acids (*a* term in LSER evaluation).^11^ These polar sites can interact either with the negatively charged analytes (RNCG) and/or with the highly abundant carbonic acid formed in the acidic mobile phase,^23^ resulting in pseudocation exchange sites enabling interactions with bases, i.e., analytes with relative positive charge on the surface (RPCG). A slight preference for the second type of interaction can be seen by comparing the weights assigned to RPCG and RNCG. The stronger interactions with analytes with a positive charge are further confirmed by pronounced positive weights of molecular descriptors related to the number of amine groups, i.e., khs.ssNH, khs.aaNH, and MDEN-23. This type of interaction has been previously described for the C8SCX column used for the analysis of alkaloids.^31^ However, our results suggest that even the HSS C18 SB column provides this type of interaction without the need for specific cation exchange moieties such as sulfonic acid, as in the case of C8SCX. Carbonic acid formation is not as pronounced using pure MeOH,^7^ leaving the polar sites on the stationary phase surface free for interactions with acids, i.e., analytes with a higher relative negative charge on the surface (RNCS). Our results suggest, similarly to the conclusions of Fu et al.,^31^ that NH_4_^+^ present in the mobile phase when using MeOH + NH_3_ may act as a competitor for interactions with these polar sites. Thus, other retention mechanisms than the positive and/or negative charge of the analytes are crucial here (Figure 4). (iv) The 2D and 3D structure of the analytes must be considered. nAtomLAC increased retention as expected due to possible interactions with C18 ligands. On the contrary, the nAtomLC counting both carbons and heteroatoms in chains decreases retention. Using MeOH + H_2_O, high positive weights of FMF are observed, showing increased retention with an increase in the cyclicity of the analytes, related to greater flexibility of the alkyl chains in the stationary phase. Wnu1.unity related to the molecular shape decreases the retention. (v) Low charge (BCUTc-1l), low polarizability (BCUTp-1l), and high LipinskiFailures of the analyte decreases retention.

**Figure 5:**
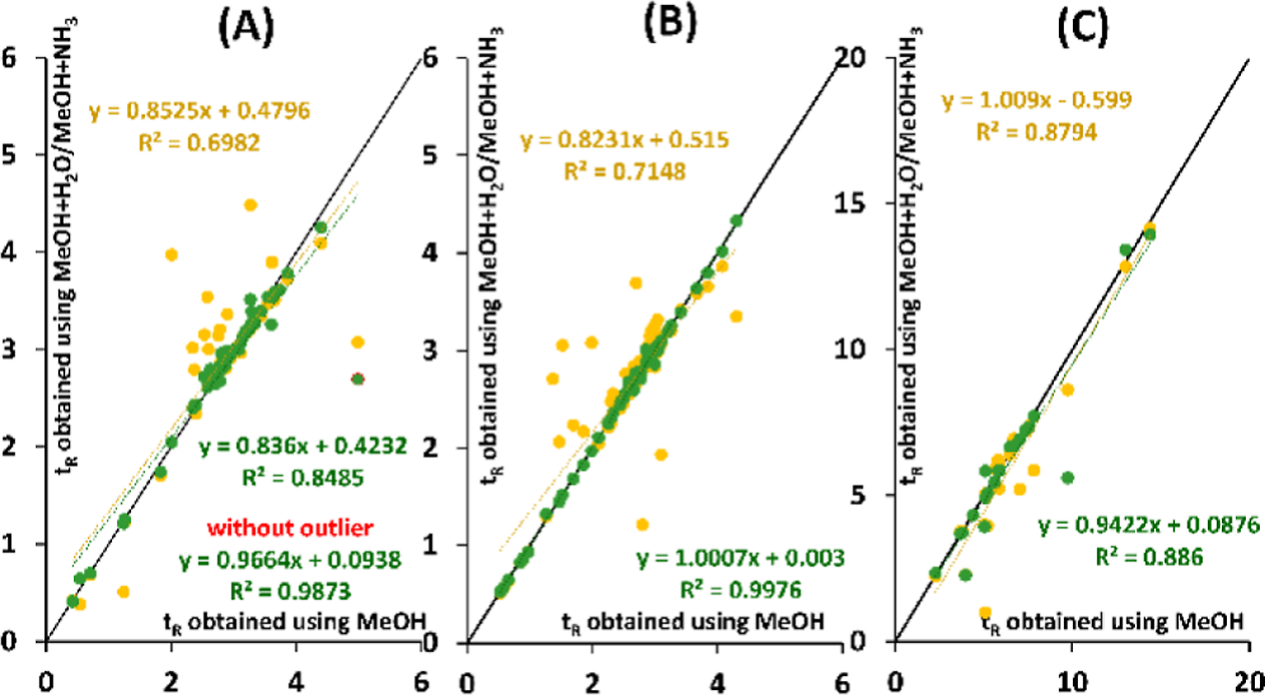
Comparison of *t*_R_ observed on (A) HSS C18 SB, (B) CSH PFP, and (C) PGC using MeOH + 2% H_2_O (green) and MeOH+10 mmol/L NH_3_ (yellow) to *t*_R_ obtained using pure MeOH. The black line corresponds to the first bisector.

Overall, the retention mechanism on the HSS C18 SB column changed significantly when using MeOH + NH_3_ as an organic modifier (*R*^2^ of 0.7). A more similar retention mechanism was observed between MeOH and MeOH + H_2_O with *R*^2^ over 0.98 and a slope close to 1 (Figure 5). However, one outlier was identified here as 3-hydroxy-4-methoxycinnamic acid, which was excluded from this comparison.

### Retention Behavior on CSH PFP

68 out of the 107 tested analytes eluted on CSH PFP using all 3 organic modifiers. Compared to the 50 analytes eluting on both C18 and CSH PFP, 18 additional compounds were analyzed with a mostly acidic nature (Supporting Information Table S5). The use of MeOH + NH_3_ allowed the elution of 23 additional compounds compared to MeOH and MeOH + H_2_O. Most of these compounds had strong alkaline properties (Supporting Information Table S5). To authentically describe the retention mechanism at each condition tested, the set of 68 analytes was used to evaluate MeOH and MeOH + H_2_O, but the expanded set of 91 analytes was used for MeOH + NH_3_.

The retention mechanism on CSH PFP was quite similar using MeOH and MeOH + H_2_O as the same molecular descriptors had the highest effect on the retention with only slightly different weights (Supporting Information Figures S10 and Figure 4). Similarly to the HSS C18 SB stationary phase, the molecular distance edge between primary and/or secondary oxygens played a crucial role in the retention on CSH PFP. This confirmed the retention of O-containing molecules due to the electrostatic interactions described by the group of C. West.^11,13,16,17^ However, the type of oxygen must be considered. Indeed, (i) increasing the distance edge between primary oxygens (MDEO-11) decreased retention, contrary to the molecular distance edge between secondary oxygens (MDEO-22) increasing retention. (ii) Similarly, a low retention of linear compounds was confirmed as the increasing number of atoms in the longest chain (nAtomLC) decreased retention. Contrary, retention increased with the total number of 7-membered rings (nRings7). However, the retention decreased significantly with an increasing number of distinct ring blocks (nRingBlocks). This means that a single aromatic system is preferable for retention on CSH PFP, as opposed to compounds with multiple aromatic parts. (iii) The branching of the molecule also needs to be accounted for, as the molecular distance edge between tertiary carbons (MDEC-33) decreased retention, whereas the edge between tertiary/quaternary (MDEC-34), secondary carbons (MDEC-22), and secondary/tertiary carbons (MDEC-23) increased retention. Similarly, retention increased with the number of =C< groups (C3SP2). (iv) A high electron density was described for CSH PFP and valence clusters expressed as VC-5, SC-4, VCH-6, and VC-6 decreased retention. (v) Low polarizability also decreased retention (BCUTp-1l). (vi) Lipophilic compounds were less retained than hydrophilic compounds, as indicated by the negative weight of XLogP (Figure 4). (vii) TPSA calculates the sum of solvent-accessible surface areas of atoms with an absolute value of partial charges ≥0.2. It was one of the most influential molecular descriptors increasing retention. Compounds with a partial charge on the surface were retained more strongly regardless of the positivity/negativity of the charge. (viii) Retention was increased for molecules with a higher number of aromatically bonded –NH groups, corresponding to the preferential retention of aromatic compounds with a partial charge on the molecular surface (khs.aaNH). (ix) Using MeOH + H_2_O, the retention was slightly more affected by the WHIM molecular descriptors related to molecular size (Wlambda1.unity, Wlambda2.unity) and dimension (WV.unity).^32^ The high values of these molecular descriptors increased the retention also using pure MeOH, but their effect was slightly less pronounced. (x) RPSA calculated as the ratio of TPSA to total molecular surface area confirmed the critical effect of charged surface for the retention on CSH PFP. FNSA-3 is a charge-weighted descriptor with values in the negative range. The weight assigned to this descriptor shows that increasing the value of FNSA-3, i.e., decreasing the negative charge, also increases retention. It confirms the same conclusions as TPSA and RPSA, but specifically for a negative charge. More pronounced interactions of positively charged analytes with the electronegative fluorine from PFP functional groups were observed, contrary to the interactions of negatively charged analytes with the positively charged stationary phase surface.^11^ Kadlecová et al., showed that CSH particles increase retention of anionic analytes at acidic mobile phase pH in reversed-phase liquid chromatography when the pyridyl surface modifier of the CSH (p*K*_a_ ∼ 5–6) is positively charged.^33,34^ Similar apparent pH is expected in CO_2_/MeOH mobile phase.^23^ As the short PFP ligand should not completely shield the CSH surface,^33^ relatively strong interactions between positively charged surface of CSH particle and negatively charged analytes are possible.

The addition of NH_3_ to the organic modifier resulted in the following changes. (i) The decreasing effect of closely located secondary oxygens was expressed by khs.dO, counting only keto oxygens instead of MDEO-22, as in the case of MeOH and MeOH + H_2_O. However, the increasing effect of closely located primary oxygens (MDEO-11) on retention remained similar. (ii) The decreasing effect of lipophilicity (XLogP) and low polarizability (BCUTp-1l) was observed with all three organic modifiers. The role of molecular size was again confirmed (Wlambda2.unity). (iii) The decreasing effect of molecular descriptors related to electron density changed. The decreasing effect of the valence cluster was still observed using MEOH + NH_3,_ as shown by VPC-6 and VC-5. However, Chi descriptors related to paths, i.e., VP-7 and SP-7, significantly increased the retention. (iv) The low retentivity of linear compounds in the case of MeOH + NH_3_ was more accurately expressed by the topoShape descriptor. It assigns a value of 1 to acyclic and 0 to cyclic compounds. As expected, its increasing value had a decreasing effect on *t*_R_. The connectivity and branching of the carbon chains played an important role, as shown by khs.dssC, MDEC-14, MDEC-22, and MDEC-34 with significantly different weights compared to MeOH and MeOH + H_2_O. (v) The aromatically bonded –NH groups (khs.aaNH) increased the retention also using MeOH + NH_3_ (Figure 4). However, the addition of NH_3_ to the organic modifier resulted in stronger interactions with compounds containing especially aliphatic amine groups (−NH–, khs.ssNH). (vi) Compounds with high MOMI-XY were less retained on CSH PFP, especially using MeOH + NH_3_ (Figure 4). Compounds with a higher density of atom distribution (Weta3.unity) were more strongly retained. (vii) The importance of the partial charge of the molecule for the interactions with the stationary phase was showed by high weight values of the ATSc5 descriptor (Supporting Information Figure S10). However, the placement of the charge on the molecular surface played a more important role when MeOH + NH_3_ was used. The effect of RPSA remained comparable to the effect when using MeOH and MeOH + H_2_O, whereas the effect of TPSA became negligible (Figure 4). The decreasing effect of FNSA-3 and the increasing effect of FNSA-1 observed using MeOH + NH_3_ confirmed the same behavior, i.e., strong interactions between compounds with negative surface charge and positively charged stationary phase surface. (viii) H-bond donor groups increased the retention using all modifiers. Such groups can easily interact with a positively charged stationary phase surface, i.e., an H-bond acceptor. However, the repulsive effect of H-bond acceptor groups became significantly more pronounced for MeOH + NH_3_. This suggests competition of compounds with the highly abundant ammonia ions in the mobile phase.

Figure 5B shows the correlation of *t*_R_ obtained using MeOH with and without additives. It further confirms similar retention mechanisms using MeOH and MeOH + H_2_O (*R*^2^ > 0.997, slope of 1.00) and different interactions contributing to the retention using MeOH + NH_3,_ (*R*^2^ 0.71, slope of 0.82). Compounds containing carboxy and hydroxy groups, such as fenoprofen, flurbiprofen, fluvastatin, *trans*-cinnamic acid, and naringenin, belong among the analytes most affected by the use of MeOH + NH_3_. However, when compared to *t*_R_ obtained using MeOH, higher *t*_R_ was observed for flurbiprofen using MeOH + NH_3_ contrary to lower *t*_R_ of fluvastatin. That confirms that other molecular properties of the analytes also play a role.

### Retention Behavior on PGC

One of the most influential molecular descriptors decreasing retention on PGC using MeOH is geomShape describing the 3D shape of the compound. It is calculated similarly to topoShape, except that the longest distance is used to define the geometrical eccentricity.^28^ GeomShape takes values from 0 to 1, with 1 representing a fully circular/spherical compound. An increasing value of GeomShape, i.e., a higher sphericity, resulted in lower retention. The descriptor Wnu2.unity, related to the molecular shape,^28^ also decreases retention. Supporting Information Figure S11 and Figure 4 show that MOMI-XY, MOMI-XZ, and MOMI-YZ related to 3D structure affected the retention on the PGC. Retention increased with increasing MOMI-XZ and MOMI-YZ values, corresponding to symmetrical top molecules and asymmetric molecules, i.e., molecules with a more planar part in the 3D shape. Aromatic rings are often responsible for planar parts of the molecular structures. Hence, an increasing number of aromatically bonded carbons (khs.aasC) enabling stronger retention on the PGC, confirmed the MOMI effect. A lower retention of branched compounds was observed, confirming previous studies.^20,22^ In our work, the branching was described by negative values of the weights of molecular descriptors such as khs.dssC (=C<), MDEC-22, C4SP3 (quaternary sp^3^ carbon), and C3SP2 (=C<). The PGC carbon network contains overlapping hybridized orbitals (sp^2^),^18^ resulting in an electron cloud available for the electron lone pair donor–acceptor interactions. Thus, C3SP2, i.e., carbons in sp^2^ hybridization, decreases *t*_R_, whereas C3SP3 (−C<), carbons in sp^3^ hybridization, increases retention. The electropositive keto group (=O) can donate an electron pair, resulting in repulsion between the PGC and such compounds (khs.dO). Contrarily, strongly electronegative fluorine groups (khs.sF) enable easy interactions with the PGC electron cloud (Figure 4).

A high polarizability of the PGC surface was previously suggested^21,35,36^ enabling polar interactions on graphite.^21^ This effect should depend on the polar groups on the surface of the compound. In our study, we observed an increasing retention with an increasing number of basic functional groups in the molecule (nBase). It suggests a partial positive charge of these groups in the weakly acidic mobile phase, enabling interactions with the electron-rich PGC surface. The molecular distance edge between secondary nitrogens (MDEN-22) increased retention. The secondary nitrogens are either part of the basic secondary amino group and/or a highly electronegative amide group. A positive effect of partial positive charge was confirmed by RPCS, i.e., relative positive charge surface area. Contrarily, RNCS, i.e., relative negative charge surface area, decreased the retention (Figure 4). Overall, compounds with a high percentage of surface covered by partial negative charge were repulsed from the PGC, resulting in their low retention. The importance of the charge state of the molecule was confirmed by the high weights of ATSc3 and BCUTc-1h, which are also based on the charge state of the compound. Increasing values of Weta1.unity related to the density of atoms distribution decreased the retention in contrast to strong lipophilic and hydrophilic properties of analytes expressed by ALogP2.

Even though the 3D structure of the analyte is expected to play a crucial role in the retention mechanism on PGC,^20,22^ the 3D shape of the molecule had almost no effect on the retention using MeOH + NH_3_ and MeOH + H_2_O. In fact, the weights assigned by ANN to these molecular descriptors were close to 0. As shown in Figure 4, the same properties of the compounds were responsible for the interactions with the stationary phase using both MeOH + NH_3_ and MeOH + H_2_O. Compared to the MeOH, (i) the 3D shape of the molecule had a negligible effect on the retention. This suggests that the carbon sheets forming the PGC stationary phase lose their planar structure. The use of additives in the SFC mobile phase changes its apparent pH.^23^ There is an abundance of ions present in the mobile phase, i.e., NH_4_^+^, H^+^, methyl hydrogen carbonate, and/or hydrogen carbonate.^37^ These ions can cause a polarization of the surface. This suggests that the structure of PGC is no longer planar, but its surface is constantly changing with convex and concave motion depending on the ions present. (ii) The value of the LipinskiFailure descriptor increases with the number of H-bond donors and acceptors in the molecule and the molecular weight of the compound. The ANN determined it to be the most important molecular descriptor, causing increased retention on PGC when MeOH with additives was used. The delocalization of π-electrons in the molecule caused by the H-bond donor and acceptor groups was described,^38^ which can result in stronger interactions with the delocalized electrons of PGC, i.e., π-cloud of graphite.^39^ The positive effect of high molecular weight was further confirmed by the positive effect of logP on retention (XLogP). (iii) Similar to pure MeOH, basic functional groups (nBase) in the molecule increased retention using MeOH + NH_3_ and MeOH + H_2_O. However, the molecular distance edge between secondary and tertiary nitrogen (MDEN-23) increased retention, contrary to MDEN-22 increasing retention using MeOH. MDEN-22 significantly decreased retention using additives. Secondary nitrogen can have strong alkaline properties within a secondary amino group or strong electronegative properties within an amide group. In contrast, tertiary nitrogen can only be a part of an alkaline tertiary amine. Thus, the electronegative properties of the nitrogen group play a more important role when MeOH with additive is used. The decreasing effect of khs.aasN (nitrogens bonded by two aromatic and one single bond) suggests that these nitrogens have their lone pair of electrons delocalized to the aromatic π-electron system. On the other hand, aromatically bonded oxygen (khs.aaO) always has at least one lone pair of electrons available for interactions with the PGC stationary phase surface, resulting in stronger retention (Figure 4). (iv) A positive effect of the electronegative fluorine group on retention, strongly pronounced using pure MeOH, significantly decreased using MeOH + NH_3_ and was almost nonexistent using MeOH + H_2_O. Unlike fluorine, chlorine has a greater resonance than an inductive effect, resulting in a possible partial positive charge on the chlorine group. The negative weight of khs.sCl, counting chlorine groups in the molecule, suggests that this localized positive partial charge is repulsed by the PGC. In contrast to MeOH, where a negative charge expressed as RNCS decreased retention, RPCS decreased retention using MeOH + NH_3_ and especially MeOH + H_2_O. We suppose that a partial positive charge is formed on the surface of the stationary phase, which repels compounds with a partial positive charge on the molecular surface. This is further confirmed by the positive weight of the RNCS in the case of MeOH + H_2_O, showing a stronger retention of compounds with a higher relative negative charge surface area. The importance of the charge state of the molecule is further confirmed by ATSc4/ATSc3 descriptors (Figure 4). (v) Branched molecules had lower retention using MeOH, but the effect of branching, represented by the number of quaternary carbons in the molecule with or without a double bond, i.e., C3SP2 and C4SP3 (Figure 4), was almost negligible for MeOH with additives. On the contrary, the high number of =C< groups (C1SP2) in the molecule slightly increased the retention for MeOH but decreased the retention when using MeOH + NH_3_ and especially MeOH + H_2_O. The positive effect of methine groups (C3SP3) remained unchanged regardless of the organic modifier. These molecular descriptors characterize the carbon connectivity in terms of hybridization, i.e., consider only bonds between carbons. Kier-Hall-Smarts descriptors count the number of occurrences of the e-state fragments. This means that khs.dsCH counts all fragments containing carbon with one double bond and one single bond regardless of the type of atom to which it is bonded to. Thus, it counts methine groups with a double bond in alkyl chains as well as aldehyde groups. The number of these groups (khs.dsCH) had a similar effect using all three organic modifiers. Khs.dssC counts fragments containing carbon with one double bond and two single bonds, such as a quaternary carbon with a double bond in the alkyl chain and/or carbons in keto groups. Since the number of keto groups (khs.dO) strongly decreased the retention using MeOH, a similar effect was attributed to khs.dssC. Keto groups had no effect on the retention using MeOH + NH_3_ and MeOH + H_2_O. Thus, the negative effect of khs.dssC was less pronounced.

Overall, the retention mechanism on the PGC column was changed by the additive in the organic modifier, as shown by lower *R*^2^ even though the slopes remained close to 1 (Figure 5C). This further confirms that other molecular properties play a crucial role in retention using organic modifiers with and without additive. While the 3D shape of the molecule mainly affected the *t*_R_ using MeOH, H-bonding, polarizability, and partial positive charge were the main interactions using additives. This difference corresponds with the lower *R*^2^ between *t*_R_ obtained using MeOH and MeOH with additives (Figure 5C).

### Retention Time Changes over Time

Changes in retention over time were observed even by an overlay of the obtained chromatograms, as well as significant differences in behavior based on the organic modifier used and the stationary phase (Supporting Information Figure S12). We can state that the *t*_R_ shifts over time depended on the stationary phase, the organic modifier, and the physicochemical properties of the analyte. Hence, SD between the molecular descriptor weights at each data point were calculated in the next step and divided into 5 groups based on their values. This comparison is shown in Supporting Information Figure S13A, and the molecular descriptors with the weights showing the most significant changes over time are listed in Supporting Information Figure S14. The most stable *t*_R_ on C18 were observed using MeOH + H_2_O, where only marginal fluctuations in molecular descriptor weights were observed. The use of MeOH and MeOH + NH_3_ resulted in similar behavior, with about 10% of the molecular descriptors having SD of weights >0.5. However, a steep shift was observed between the first injection and analyses after one month using MeOH + NH_3_ (Figure 2). This suggests that the additive did not sufficiently cover the stationary phase after the first equilibration procedure and a significantly higher number of column volumes should be used. Using MeOH, the molecular descriptors affected by the highest extent were related to the density of atoms distribution (Weta2.unity), charge state of the molecule (ATSc3, ATSc4), carbon connectivity (MDEC-13), and valence electrons (SC-5). The effect of charge (BCUTc-1l), valence electrons (SC-5, SC-6), and carbon connectivity (MDEC-12, MDEC-24) changed the most over time using MeOH + NH_3_. The most stable *t*_R_ on CSH PFP were observed using MeOH, followed by MeOH + H_2_O and MeOH + NH_3_ (Supporting Information Figure S13). All molecular descriptors were stable within SD < 0.3 over 12 months using MeOH. Five molecular descriptors, i.e., BCUTp-1l, FMF, khs.ssS, MDEC-22, and MDEN-23, had SD of 0.3–0.5 using MeOH + H_2_O. The most affected molecular descriptor using MeOH + NH_3_ was charge-related BCUTc-1l with SD of 1.1, followed by MDEC-12, MDEC-24, SC-5, and SC-6. *t*_R_ on the PGC stationary phase and related molecular descriptor weights were stable over time using MeOH and MeOH + H_2_O. The highest differences were observed using MeOH + NH_3_ where the SD of LipinskiFailure, i.e., the most influential molecular descriptor, was 1.4, followed by molecular descriptors related to the carbon chains (nAtomLC, nAtomLAC, and khs.dsCH). However, the lower number of compounds eluting on PGC decreases the statistical significance of these observations.

### Regeneration Procedure

**Figure 6:**
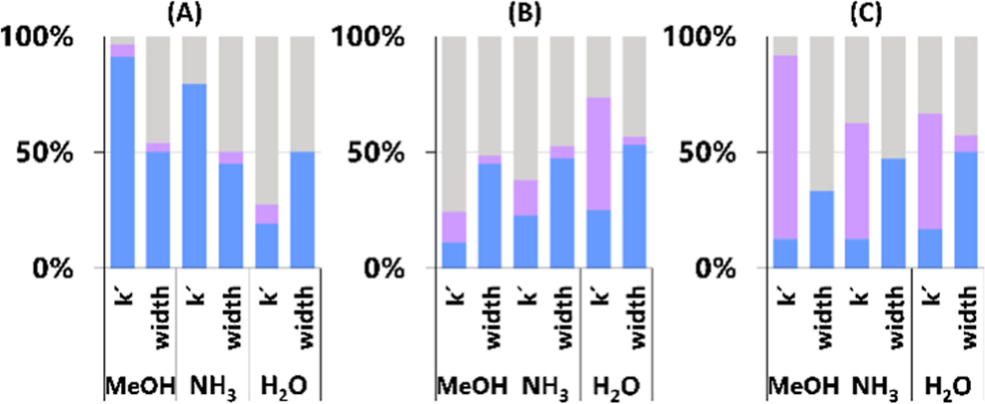
Comparison of changes in retention and peak width: value closer to the value of the first injection after regeneration (blue) vs after 12 months (gray). Changes within ±1% are in purple. (A) HSS C18 SB, (B) CSH PFP, (C) PGC.

The regeneration procedure resulted in approximately 80% of analytes with *t*_R_ within ±1% of the original *t*_R_ on the PGC and on the C18 when using MeOH + NH_3_ (Supporting Information Figure S13B). This further confirms the critical role of ammonia ions in sufficiently covering the C18 stationary phase. The ammonia ions were washed away from the stationary phase surface during the regeneration process. Thus, the subsequent equilibration was again insufficient for the repeatable coverage, resulting in *t*_R_ close to the M0. However, a shift closer to the *t*_R_ observed at M2 is expected in the subsequent analysis. The regeneration procedure was completely unsuccessful in the case of the CSH PFP. More than 80% of compounds had *t*_R_ differences >1% between the first injection and the after-regeneration injection. Nevertheless, the *t*_R_ shifts that occurred during the 12 months of column use differed for each column. The second comparison of *k*′ was carried out to show whether the regeneration procedure reduced the *t*_R_ shifts compared to the shifts observed at M12 (Figure 6). The regeneration had a beneficial effect on *k*′ using C18 and MeOH and/or MeOH + NH_3_. On the contrary, a rather negative effect was observed in the case of MeOH + H_2_O on C18 and all three organic modifiers on CSH PFP. The unsuccessful regeneration procedure could be attributed to the insufficient wash of adsorbed additives, the inability to restore silanol groups, and/or permanent damage occurring of the columns. As all columns were used within the manufacturer-recommended limits, less than 300 injections of standard samples were carried out, and the *t*_R_ and peak shapes changed gradually to different extents for various compounds, the possibility of permanent damage of the columns was not determined as probable. Furthermore, unaffected analytes were also observed.

The regeneration procedure did not significantly affect the retention on PGC. This was expected since the regeneration protocol was established based on recommendations for silica-based columns. Typical PGC washing protocols include the use of strong acids/bases and solvents such as tetrahydrofuran, acetone, and trifluoroacetate.^22^ Thus, the washing with water was probably insufficient to return the PGC column to the original redox state.

## Conclusion

The retention mechanisms on three nonpolar stationary phases, HSS C18 SB, CSH PFP, and PGC, were defined using molecular descriptors explored by ANN. ANN-assigned weights enabled to describe the changes in retention using MeOH, MeOH + 10 mmol/L NH_3_, and MeOH + 2% H_2_O.

We proved the possibility of cation exchange interactions on HSS C18 SB using MeOH + NH_3_. The flexibility of alkyl chains is also affected by the organic modifier composition, which alters the retention. Completely different interaction mechanisms were described for PGC when using MeOH with and without additives. This may suggest modifications to the planar structure and surface polarizability of the PGC.

The best stability over one year of column use was observed for C18 using MeOH + H_2_O, while a significant retention decrease was noticed for MeOH + NH_3_. A similar behavior was also determined for MeOH, confirming the possible SEF. Retention times on CSH PFP were stable using MeOH and MeOH + NH_3_, with the lowest stability achieved for MeOH + H_2_O. Surprisingly, similar trends were also observed for PGC, where the stability was expected to be independent of the organic modifier. However, the low number of compounds eluting from PGC decreases the statistical significance of these results. The regeneration procedure positively affected the *k*′ for C18 using MeOH and MeOH + NH_3_. In addition, the peak width was improved for 50% of the compounds. A significant improvement was also observed for PGC, but the regeneration procedure did not meet the expectations for CSH PFP. The in-depth exploration and understanding of the fundamental retention aspects under different SFC conditions can be used for the evidence-based selection of the stationary phase for targeted application. This approach not only increases the efficiency of method development but also reduces the number of experiments required, contributing to a more environmentally friendly analytical workflow.

## Data Availability

The original data used in this publication are openly available in Zenodo under the doi: 10.5281/zenodo.14651083.

## Supporting Information Available

List of analytes; list of molecular descriptors; chemical structures and LSER parameters; additional compounds eluting on HSS C18 SB and CSH PFP; Heatmaps of molecular descriptor weights; PCA of analytes; Boxplots of *t*_R_; descriptors with the highest differences in weights; graphical representation of MOMI descriptors; key molecular descriptors for each stationary phase; SFC-UV chromatograms of selected analytes at different data points; SD between molecular descriptors at different data points; molecular descriptors with the highest change over time (PDF)

## References

[ref1] Plachká K.; Pilařová V.; Horáček O.; Gazárková T.; Vlčková H. K.; Kučera R.; Nováková L. Columns in analytical-scale supercritical fluid chromatography: From traditional to unconventional chemistries. J. Sep. Sci. 2023, 46, 2300431 10.1002/jssc.202300431. 37568246

[ref2] Losacco G. L.; Veuthey J.-L.; Guillarme D. Metamorphosis of supercritical fluid chromatography: A viable tool for the analysis of polar compounds?. TrAC, Trends Anal. Chem. 2021, 141, 116304 10.1016/j.trac.2021.116304.

[ref3] Si-Hung L.; Bamba T. Current state and future perspectives of supercritical fluid chromatography. TrAC, Trends Anal. Chem. 2022, 149, 116550 10.1016/j.trac.2022.116550.

[ref4] West C.; Lesellier E. Chapter 3 - Selection of SFC stationary and mobile phases. In Separation Science and Technology; Hicks M., Ferguson P., Eds.; Academic Press, 2022; Vol. 14, pp 49–71.

[ref5] Pilařová V.; Plachká K.; Gazárková T.; Svec F.; Garrigues J. C.; Nováková L. Using artificial neural networks to elucidate retention interactions on stationary phases with amine moieties dedicated to supercritical fluid chromatography. Sep. Purif. Technol. 2025, 356, 129965 10.1016/j.seppur.2024.129965.

[ref6] Plachká K.; Pilařová V.; Gazárková T.; Svec F.; Garrigues J. C.; Nováková L. Advancing Fundamental Understanding of Retention Interactions in Supercritical Fluid Chromatography Using Artificial Neural Networks: Polar Stationary Phases with – OH Moieties. Anal. Chem. 2024, 96, 12748–12759. 10.1021/acs.analchem.4c01811. 39069659 PMC11307250

[ref7] West C.; Lemasson E. Unravelling the effects of mobile phase additives in supercritical fluid chromatography—Part II: Adsorption on the stationary phase. J. Chromatogr. A 2019, 1593, 135–146. 10.1016/j.chroma.2019.02.002. 30803789

[ref8] Poole C. F. Stationary phases for packed-column supercritical fluid chromatography. J. Chromatogr. A 2012, 1250, 157–171. 10.1016/j.chroma.2011.12.040. 22209357

[ref9] Fairchild J. N.; Brousmiche D. W.; Hill J. F.; Morris M. F.; Boissel C. A.; Wyndham K. D. Chromatographic Evidence of Silyl Ether Formation (SEF) in Supercritical Fluid Chromatography. Anal. Chem. 2015, 87, 1735–1742. 10.1021/ac5035709. 25514458

[ref10] Waters Corporation. ACQUITY UPC^2^ BEH, HSS, and CSH Columns Care and Use Manual; Waters Corporation: USA, 2015.

[ref11] West C.; Lemasson E.; Bertin S.; Hennig P.; Lesellier E. An improved classification of stationary phases for ultra-high performance supercritical fluid chromatography. J. Chromatogr. A 2016, 1440, 212–228. 10.1016/j.chroma.2016.02.052. 26920664

[ref12] Khater S.; West C.; Lesellier E. Characterization of five chemistries and three particle sizes of stationary phases used in supercritical fluid chromatography. J. Chromatogr. A 2013, 1319, 148–159. 10.1016/j.chroma.2013.10.037. 24377105

[ref13] Lesellier E. Usual, unusual and unbelievable retention behavior in achiral supercritical fluid chromatography: Review and discussion. J. Chromatogr. A 2020, 1614, 460582 10.1016/j.chroma.2019.460582. 31604584

[ref14] Lesellier E. Extension of the carotenoid test to superficially porous C18 bonded phases, aromatic ligand types and new classical C18 bonded phases. J. Chromatogr. A 2012, 1266, 34–42. 10.1016/j.chroma.2012.09.068. 23116802

[ref15] West C.; Lemasson E.; Khater S.; Lesellier E. An attempt to estimate ionic interactions with phenyl and pentafluorophenyl stationary phases in supercritical fluid chromatography. J. Chromatogr. A 2015, 1412, 126–138. 10.1016/j.chroma.2015.08.009. 26278356

[ref16] West C.; Lesellier E. Characterisation of stationary phases in subcritical fluid chromatography with the solvation parameter model IV: Aromatic stationary phases. J. Chromatogr. A 2006, 1115, 233–245. 10.1016/j.chroma.2006.02.050. 16529759

[ref17] Si-Hung L.; Bamba T. A review of retention mechanism studies for packed column supercritical fluid chromatography. Anal. Sci. Adv. 2021, 2, 47–67. 10.1002/ansa.202000144. 38715740 PMC10989630

[ref18] De Matteis C. I.; Simpson D. A.; Euerby M. R.; Shaw P. N.; Barrett D. A. Chromatographic retention behaviour of monosubstituted benzene derivatives on porous graphitic carbon and octadecyl-bonded silica studied using molecular modelling and quantitative structure–retention relationships. J. Chromatogr. A 2012, 1229, 95–106. 10.1016/j.chroma.2011.12.090. 22305358

[ref19] Pereira L. Porous Graphitic Carbon as a Stationary Phase in HPLC: Theory and Applications. J. Liq. Chromatogr. Relat. Technol. 2008, 31, 1687–1731. 10.1080/10826070802126429.

[ref20] West C.; Lesellier E.; Tchapla A. Retention characteristics of porous graphitic carbon in subcritical fluid chromatography with carbon dioxide–methanol mobile phases. J. Chromatogr. A 2004, 1048, 99–109. 10.1016/S0021-9673(04)01107-0. 15453424

[ref21] West C.; Elfakir C.; Lafosse M. Porous graphitic carbon: A versatile stationary phase for liquid chromatography. J. Chromatogr. A 2010, 1217, 3201–3216. 10.1016/j.chroma.2009.09.052. 19811787

[ref22] Bapiro T. E.; Richards F. M.; Jodrell D. I. Understanding the Complexity of Porous Graphitic Carbon (PGC) Chromatography: Modulation of Mobile-Stationary Phase Interactions Overcomes Loss of Retention and Reduces Variability. Anal. Chem. 2016, 88, 6190–6194. 10.1021/acs.analchem.6b01167. 27228284 PMC5362737

[ref23] West C.; Melin J.; Ansouri H.; Mengue Metogo M. Unravelling the effects of mobile phase additives in supercritical fluid chromatography. Part I: Polarity and acidity of the mobile phase. J. Chromatogr. A 2017, 1492, 136–143. 10.1016/j.chroma.2017.02.066. 28274478

[ref24] Plachká K.; Střítecký J.; Svec F.; Nováková L. The effect of column history in supercritical fluid chromatography: Practical implications. J. Chromatogr. A 2021, 1651, 462272 10.1016/j.chroma.2021.462272. 34107402

[ref25] Lafossas C.; Benoit-Marquié F.; Garrigues J. C. Analysis of the retention of tetracyclines on reversed-phase columns: Chemometrics, design of experiments and quantitative structure-property relationship (QSPR) study for interpretation and optimization. Talanta 2019, 198, 550–559. 10.1016/j.talanta.2019.02.051. 30876599

[ref26] Lipinski C. A.; Lombardo F.; Dominy B. W.; Feeney P. J. Experimental and computational approaches to estimate solubility and permeability in drug discovery and development settings. Adv. Drug Delivery Rev. 1997, 23, 3–25. 10.1016/S0169-409X(96)00423-1. 11259830

[ref27] Petitjean M. Applications of the radius-diameter diagram to the classification of topological and geometrical shapes of chemical compounds. J. Chem. Inf. Comput. 1992, 32, 331–337. 10.1021/ci00008a012.

[ref28] Bath P. A.; Poirrette A. R.; Willett P.; Allen F. H. The Extent of the Relationship between the Graph-Theoretical and the Geometrical Shape Coefficients of Chemical Compounds. J. Chem. Inf. Comput. 1995, 35, 714–716. 10.1021/ci00026a007.

[ref29] Muteki K.; Morgado J. E.; Reid G. L.; Wang J.; Xue G.; Riley F. W.; Harwood J. W.; Fortin D. T.; Miller I. J. Quantitative Structure Retention Relationship Models in an Analytical Quality by Design Framework: Simultaneously Accounting for Compound Properties, Mobile-Phase Conditions, and Stationary-Phase Properties. Ind. Eng. Chem. Res. 2013, 52, 12269–12284. 10.1021/ie303459a.

[ref30] Rafferty J. L.; Siepmann J. I.; Schure M. R. Mobile phase effects in reversed-phase liquid chromatography: A comparison of acetonitrile/water and methanol/water solvents as studied by molecular simulation. J. Chromatogr. A 2011, 1218, 2203–2213. 10.1016/j.chroma.2011.02.012. 21388628

[ref31] Fu Q.; Dong W.; Ge D.; Ke Y.; Jin Y. Supercritical fluid chromatography based on reversed-phase/ion chromatography mixed-mode stationary phase for separation of spirooxindole alkaloids. J. Chromatogr. A 2023, 1705, 464163 10.1016/j.chroma.2023.464163. 37348226

[ref32] Hall L. H.; Kier L. B. The Molecular Connectivity Chi Indexes and Kappa Shape Indexes in Structure-Property Modeling. In Reviews in Computational Chemistry; John Wiley & Sons, 1991; pp 367–422.

[ref33] Kadlecová Z.; Kalíková K.; Folprechtová D.; Tesařová E.; Gilar M. Method for evaluation of ionic interactions in liquid chromatography. J. Chromatogr. A 2020, 1625, 461301 10.1016/j.chroma.2020.461301. 32709344

[ref34] Walter T. H.; Alden B. A.; Field J. A.; Lawrence N. L.; Osterman D. L.; Patel A. V.; DeLoffi M. A. Characterization of a highly stable mixed-mode reversed-phase/weak anion-exchange stationary phase based on hybrid organic/inorganic particles. J. Sep. Sci. 2021, 44, 1005–1014. 10.1002/jssc.202001136. 33354922 PMC7986357

[ref35] Bassler B. J.; Kaliszan R.; Hartwick R. A. Retention mechanisms on metallic stationary phases. J. Chromatogr. A 1989, 461, 139–147. 10.1016/S0021-9673(00)94283-3.

[ref36] Polyakova Y.; Ho Row K. HPLC of Some Polar Compounds on a Porous Graphitized Carbon HypercarbTM Column. J. Liq. Chromatogr. Relat. Technol. 2005, 28, 3157–3168. 10.1080/10826070500330687.

[ref37] Oparin R. D.; Krestyaninov M. A.; Vorobyev E. A.; Pokrovskiy O. I.; Parenago O. O.; Kiselev M. G. An insight into possibility of chemical reaction between dense carbon dioxide and methanol. J. Mol. Liq. 2017, 239, 83–91. 10.1016/j.molliq.2016.12.027.

[ref38] Sobczyk L.; Grabowski S. J.; Krygowski T. M. Interrelation between H-Bond and Pi-Electron Delocalization. Chem. Rev. 2005, 105, 3513–3560. 10.1021/cr030083c. 16218560

[ref39] Russo M.; Camillo M. R. T.; La Tella R.; Rigano F.; Donato P.; Mondello L.; Dugo P. Principles and applications of porous graphitic carbon stationary phase in liquid chromatography: An update. J. Chromatogr. A 2024, 1719, 464728 10.1016/j.chroma.2024.464728. 38402696

