# Supplementary material for: AI-Enhanced Understanding of Retention Interactions in Supercritical Fluid Chromatography: Neural Network Insights into Retention on Selected Non-Polar Stationary Phases
Source: Anal Chem. 2025 Jan 21;97(4):2164–75. doi: 10.1021/acs.analchem.4c05176 (PMC11800175; doi:10.1021/acs.analchem.4c05176)
Supplement: Supplementary file 1 — ac4c05176_si_001.pdf [file ac4c05176_si_001.pdf]

Supporting Information: AI-Enhanced Understanding of Retention Interactions in Supercritical Fluid Chromatography: Neural Network Insights into Retention on Selected Non-Polar Stationary Phases

Kateřina Plachká[1], Veronika Pilařová[1], Taťána Gazárková[1], František Švec[1], Jean-Christophe Garrigues[2], Lucie Nováková[1*]

[1] Department of Analytical Chemistry, Faculty of Pharmacy in Hradec Králové, Charles University, Hradec Králové, Czech Republic

2 SOFTMAT (IMRCP) Laboratory, SMODD Team, CNRS, Toulouse III Paul Sabatier University, Toulouse, France

S1. Experimental Part

S1. Description of artificial neural network algorithm in Matlab.

Table S1: List of analytes; their physicochemical properties and elution on tested columns using three organic modifiers, i.e., MeOH, MeOH + 10 mM NH_3, and MeOH + 2% H_2O.

Table S2: Molecular descriptors used in the study, calculated with CDK Descriptor Calculator (v.1.4.8).

Table S3: Molecular descriptors used in the study grouped by primary category.

Figure S1. (A) The chemical structure of tested stationary phases, (B) the effect of LSER parameters on HSS C18 SB, CSH PFP, and PGC columns, and (C) a spider diagram characterizing stationary phases based on LSER.

S2. Results

Table S4. Compounds eluting from HSS C18 SB column using MeOH+NH_3 instead of MeOH and MeOH+H_2O.

Table S5: Compounds eluting from CSH PFP contrary to HSS C18 SB column.

Figure S2. Heatmap of molecular descriptor weights representing their effect on the retention on HSS C18 SB column determined by ANN.

Figure S3. Heatmap of molecular descriptor weights representing their effect on the retention on CSH PFP column determined by ANN.

Figure S4. Heatmap of molecular descriptor weights representing their effect on the retention on PGC column determined by ANN.

Figure S5. Principal Component Analysis of analytes eluting on each of the tested stationary phase, i.e., HSS C18 SB (blue), CSH PFP (green), PGC (red) based on their molecular descriptors.

Figure S6. Boxplots of retention factors of compounds eluting on each stationary phase using methanol (blue), 10 mmol/L ammonia in methanol (yellow), and 2% water in methanol (green) as organic modifier.

Figure S7. Comparison of molecular descriptors with the highest differences in weights assigned by ANN to HSS C18 SB (blue) and CSH PFP (purple) stationary phase, using MeOH as organic modifier and the common set of 50 analytes (Table S1).

Figure S8. Comparison of weights of key molecular descriptors affecting retention on HSS C18 SB. MeOH (dark purple), MeOH+10 mmol/L NH_3 (purple), and MeOH+2% H_2O (pink) as organic modifier, with a set of 57 eluted analytes (Table S1).

Figure S9. Description and general representation of the moment of inertia (MOMI) molecular descriptor.

Figure S10. Comparison of weights of key molecular descriptors affecting retention on CSH PFP. MeOH (dark purple), MeOH+10 mmol/L NH_3 (purple), and MeOH+2% H_2O (pink) as organic modifier, with a set of 68 eluted analytes (Table S1).

Figure S11. Comparison of weights of key molecular descriptors affecting retention on PGC. MeOH (dark purple), MeOH+10 mmol/L NH_3 (purple), and MeOH+2% H_2O (pink) as organic modifier, with a set of 28 eluted analytes (Table S1).

Figure S12. SFC-UV chromatograms of selected analytes collected at different data points using (A) HSS C18 SB/MeOH+10 mmol/L ammonia, (B) HSS C18 SB/MeOH+2% water, (C) CSH PFP/MeOH+10 mmol/L ammonia, (D) CSH PFP/MeOH+2% water. 1st injection (dark blue), month 1 (light blue), month 2 (dark green), month 3 (light green), month 6 (dark yellow), month 9 (yellow), and month 12 (red).

Figure S13. (A) Percentage of molecular descriptors with standard deviations between weights determined by ANN at each data point: < 0.1 (dark blue), 0.1-0.3 (light blue), 0.3-0.5 (yellow), 0.5-1.0 (light red), > 1.0 (dark red). (B) Percentage of compounds with %-error between t_R at the 1st injection and after regeneration procedure: < 0.5 (dark blue), 0.5-1.0 (light blue), 1.0-2.0 (yellow), 2.0-5.0 (light red), > 5.0 (dark red).

Figure S14. The molecular descriptors with the weights with the largest changes over time on HSS C18 SB, CSH PFP, and PGC columns using (A) MeOH, (B) MeOH + 10mmol/L ammonia, (C) MeOH + 2% H_2O. Weights determined by ANN based on analysis at 1st injection (dark blue), month 1 (light blue), month 2 (dark green), month 3 (light green), month 6 (dark yellow), month 9 (yellow), and month 12 (red).

S1. Description of artificial neural network algorithm in Matlab.

The Matlab algorithm employed for training the artificial neural network (ANN) incorporates multiple functions to ensure training termination based on ANN performance, thereby guaranteeing the validity of the results within the software framework. Training was halted under the following conditions: reaching the maximum number of epochs, exceeding the maximum allowed time, achieving the target performance value, the performance gradient falling below the target threshold, or an increase in validation error beyond the target value after the last drop.

Additionally, several performance parameters were monitored during ANN training, including the change in cross-entropy across training epochs, the training gradient values, and the error histogram, which represents the differences between target and output values. Throughout the evaluation, all observed errors remained consistently below 0.05.

S1. Experimental Part

Table S1: List of analytes; their main physicochemical properties, including molecular weight (MW), acidic/basic properties (pKa), lipophilicity (logP), number of H acceptors and donors, and molecular formula; and elution on tested columns (HSS C18 SB, CSH PFP, and PGC) using three tested organic modifiers, namely MeOH, MeOH + 10 mM NH_3, and MeOH + 2% H_2O. n/a indicates, that analyte did not elute that analyte did not elute on any of the d on any of columns.

n.	CAS No.	analyte	Mw	pKa acid	pKa basic	log P	H acc.	H donor	molecular formula	MeOH	MeOH + 10 mM NH_3	MeOH + 2% H_2O
**1**	841-67-8	(-)-thalidomide	258.23	10.7	-2.55	0.33	1	7	$C_{13}H_{10}N_2O_4$	HSS C18; CSH FP; PGC	HSS C18; CSH FP; PGC	HSS C18; CSH FP; PGC
**2**	2614-06-4	(+)-thalidomide	258.23	10.7	-2.55	0.33	1	7	$C_{13}H_{10}N_2O_4$	HSS C18; CSH FP; PGC	CSH FP; PGC	HSS C18; CSH FP; PGC
**3**	303-38-8	2,3-dihydroxy benzoic acid	154.12	2.96	-	1.62	4	3	$C_7H_6O_4$	n/a	CSH FP	n/a
**4**	89-86-1	2,4-dihydroxy benzoic acid	154.12	3.32	-	1.77	4	3	$C_7H_6O_4$	n/a	CSH FP	n/a
**5**	490-79-9	2,5-dihydroxy benzoic acid	154.12	3.01	-	1.40	4	3	$C_7H_6O_4$	n/a	CSH FP	n/a
**6**	303-07-1	2,6-dihydroxy benzoic acid	154.12	1.3	-	2.38	4	3	$C_7H_6O_4$	n/a	CSH FP	n/a
**7**	583-17-5	2-hydroxy cinnamic acid	164.16	4.51	-	1.02	3	2	$C_9H_8O_3$	HSS C18	HSS C18 ; CSH FP	HSS C18 ; CSH FP
**8**	362-07-2	2-methoxyestradiol	302.41	10.29	-	3.84	3	2	$C_{19}H_{26}O_3$	HSS C18 ; CSH FP	HSS C18 ; CSH FP	HSS C18 ; CSH FP
**9**	99-50-3	3,4-dihydroxy benzoic acid	154.12	4.45	-	1.01	4	3	$C_7H_6O_4$	n/a	n/a	n/a
**10**	99-10-5	3,5-dihydroxy benzoic acid	154.12	3.96	-	0.81	4	3	$C_7H_6O_4$	HSS C18 ; CSH FP	HSS C18 ; CSH FP	HSS C18 ; CSH FP
**11**	530-59-6	4-hydroxy-3,5-dimethoxy-cinnamic acid	224.21	4.53	-	1.00	5	2	$C_{11}H_{12}O_5$	HSS C18 ; CSH FP	HSS C18 ; CSH FP	HSS C18 ; CSH FP
**12**	588-30-7	3-hydroxy cinnamic acid	164.16	4.38	-	0.93	3	2	$C_9H_8O_3$	HSS C18 ; CSH FP	HSS C18 ; CSH FP	HSS C18 ; CSH FP
**13**	537-73-5	3-hydroxy-4-methoxycinnamic acid	194.18	4.53	-	0.79	4	2	$C_{10}H_{10}O_4$	HSS C18 ; CSH FP	HSS C18 ; CSH FP	HSS C18 ; CSH FP
**14**	530-57-4	4-hydroxy-3,5-dimethoxybenzoic acid	198.17	4.33	-	1.28	5	2	$C_9H_{10}O_5$	HSS C18 ; CSH FP	HSS C18 ; CSH FP	HSS C18 ; CSH FP
**15**	121-34-6	4-hydroxy-3-methoxy benzoic acid	168.15	4.45	-	1.30	4	2	$C_8H_8O_4$	HSS C18 ; CSH FP	HSS C18 ; CSH FP	HSS C18 ; CSH FP
**16**	99-96-7	4-hydroxybenzoic acid	138.12	4.57	-	1.40	3	2	$C_7H_6O_3$	HSS C18 ; CSH FP	HSS C18 ; CSH FP	HSS C18 ; CSH FP
**17**	100-09-4	4-methoxy benzoic acid	152.15	4.47	-	1.78	3	1	$C_8H_8O_3$	CSH FP; PGC	HSS C18; CSH FP; PGC	CSH FP; PGC
**18**	154229-18-2	abiraterone acetate	391.55	5.31	-	6.58	3	0	$C_{26}H_{33}NO_2$	CSH FP	HSS C18 ; CSH FP	HSS C18 ; CSH FP
**19**	37517-30-9	acebutolol	336.43	13.78	9.4	1.77	6	3	$C_{18}H_{28}N_2O_4$	PGC	CSH FP; PGC	PGC
**20**	138112-76-2	agomelatine	243.3	16.17	-0.53	2.47	3	1	$C_{15}H_{17}NO_2$	HSS C18 ; CSH FP	HSS C18 ; CSH FP	HSS C18 ; CSH FP
**21**	52-39-1	aldosterone	360.44	12.98	-	0.70	2	7	$C_{21}H_{28}O_5$	HSS C18 ; CSH FP	HSS C18 ; CSH FP	HSS C18 ; CSH FP
**22**	59-02-9	alpha-tocopherol	430.71	11.4	-	10.96	2	1	$C_{29}H_{50}O_2$	HSS C18 ; CSH FP	HSS C18 ; CSH FP	HSS C18 ; CSH FP

n.	CAS No.	analyte	Mw	pKa acid	pKa basic	log P	H acc.	H donor	molecular formula	MeOH	MeOH + 10 mM NH_3	MeOH + 2% H_2O
**23**	1721-51-3	alpha-tocotrienol	424.66	11.4	-	9.70	2	1	$C_{29}H_{44}O_2$	HSS C18; CSH PFP	HSS C18; CSH PFP	HSS C18; CSH PFP
**24**	520-36-5	apigenin	270.24	6.53	-	2.13	5	3	$C_{15}H_{10}O_5$	n/a	n/a	n/a
**25**	29122-68-7	atenolol	266.34	13.88	9.43	0.36	5	4	$C_{14}H_{22}N_2O_3$	n/a	CSH PFP	n/a
**26**	98-55-5	a-terpinol	154.25	14.94	-	2.54	1	2	$C_{10}H_{18}O$	HSS C18; CSH PFP	HSS C18; CSH PFP	HSS C18; CSH PFP
**27**	83015-26-3	atomoxetine	255.35	10.15	-	3.36	2	1	$C_{17}H_{21}NO$	n/a	HSS C18; CSH PFP	n/a
**28**	134523-00-5	atorvastatin	558.64	4.29	0.38	3.85	7	4	$C_{33}H_{35}FN_2O_5$	CSH PFP	CSH PFP	CSH PFP
**29**	148-03-8	beta-tocopherol	416.68	11.05	-	10.72	2	1	$C_{28}H_{48}O_2$	HSS C18	HSS C18; CSH PFP	HSS C18
**30**	490-23-3	beta-tocotrienol	410.63	11.05	-	9.46	2	1	$C_{28}H_{42}O_2$	HSS C18	HSS C18; CSH PFP	HSS C18
**31**	378-44-9	betamethasone	392.46	12.13	-	2.03	5	3	$C_{22}H_{29}FO_5$	HSS C18; CSH PFP	HSS C18; CSH PFP	HSS C18; CSH PFP
**32**	62658-63-3	bopindolol	380.48	17.59	9.4	4.82	5	2	$C_{23}H_{28}N_2O_3$	PGC	HSS C18; CSH PFP; PGC	PGC
**33**	51-20-7	bromouracil	190.98	6.77	-	-0.21	4	2	$C_4H_3BrN_2O_2$	HSS C18; CSH PFP	HSS C18; CSH PFP	HSS C18; CSH PFP
**34**	58-08-2	caffeine	194.19	0.52	-	-0.63	6	0	$C_8H_{10}N_4O_2$	HSS C18; CSH PFP	HSS C18; CSH PFP	HSS C18; CSH PFP
**35**	154-23-4	catechin	290.27	9.54	-	0.61	5	11	$C_{15}H_{14}O_6$	n/a	n/a	n/a
**36**	83881-51-0	cetirizine	388.89	3.46	6.71	1.62	5	1	$C_{21}H_{25}ClN_2O_3$	n/a	HSS C18; CSH PFP	CSH PFP
**37**	50-22-6	corticosterone	346.46	12.98	-	1.95	2	6	$C_{21}H_{30}O_4$	HSS C18; CSH PFP	HSS C18; CSH PFP	HSS C18; CSH PFP
**38**	71-30-7	cytosine	111.1	9	4.18	-1.96	4	3	$C_4H_5N_3O$	n/a	HSS C18; CSH PFP	n/a
**39**	1009119-64-5	daclatasvir	738.88	10.92	6.56	2.51	14	4	$C_{40}H_{50}N_8O_6$	CSH PFP	HSS C18; CSH PFP	CSH PFP
**40**	133099-04-4	darifenacin	426.55	15.7	9.32	3.78	4	2	$C_{28}H_{30}N_2O_2$	n/a	HSS C18; CSH PFP	n/a
**41**	302962-49-8	dasatinib	488.01	10.94	7.29	0.14	9	3	$C_{22}H_{26}ClN_7O_2S$	n/a	CSH PFP	CSH PFP
**42**	53-43-0	dehydro-epi-androsterone	288.43	15.02	-	3.31	1	3	$C_{19}H_{28}O_2$	HSS C18; CSH PFP	HSS C18; CSH PFP	HSS C18; CSH PFP
**43**	119-13-1	delta-tocopherol	402.65	10.7	-	10.49	2	1	$C_{27}H_{46}O_2$	HSS C18	HSS C18	HSS C18
**44**	25612-59-3	delta-tocotrienol	396.61	10.69	-	9.23	2	1	$C_{27}H_{40}O_2$	HSS C18	HSS C18	HSS C18
**45**	50-47-5	desipramine	266.38	-	10.4	3.97	2	1	$C_{18}H_{22}N_2$	CSH PFP	HSS C18; CSH PFP	n/a

n.	CAS No.	analyte	Mw	pKa acid	pKa basic	log P	H acc.	H donor	molecular formula	MeOH	MeOH + 10 mM NH_3	MeOH + 2% H_2O
**46**	50-02-2	dexamethasone	392.46	12.13	-	2.03	5	3	$C_{22}H_{29}FO_5$	HSS C18; CSH PFP; PGC	HSS C18; CSH PFP; PGC	HSS C18; CSH PFP; PGC
**47**	915087-33-1	enzalutamide	464.44	13.88	-1.99	2.98	6	1	$C_{21}H_{16}F_4N_4O_2S$	HSS C18; CSH PFP; PGC	HSS C18; CSH PFP; PGC	HSS C18; CSH PFP; PGC
**48**	57-91-0	estradiol	272.38	10.27	-	4.15	2	2	$C_{18}H_{24}O_2$	HSS C18; CSH PFP	HSS C18; CSH PFP	HSS C18; CSH PFP
**49**	4245-41-4	estradiol acetate	314.42	15.06	-	4.46	3	1	$C_{20}H_{26}O_3$	HSS C18; CSH PFP	HSS C18; CSH PFP	HSS C18; CSH PFP
**50**	50-27-1	estriol	288.38	10.25	-	2.53	3	3	$C_{18}H_{24}O_3$	HSS C18; CSH PFP	HSS C18; CSH PFP	HSS C18; CSH PFP
**51**	53-16-7	estron	270.37	10.25	-	3.62	2	1	$C_{18}H_{22}O_2$	HSS C18; CSH PFP	HSS C18; CSH PFP	HSS C18; CSH PFP
**52**	774-40-3	(±)-ethyl mandelate	194.23	12.31	-	1.76	3	1	$C_{10}H_{12}O_3$	HSS C18; CSH PFP; PGC	HSS C18; CSH PFP; PGC	HSS C18; CSH PFP; PGC
**53**	470-82-6	eucalyptol (1,8-cineole)	154.25	-	-4.2	2.80	0	1	$C_{10}H_{18}O$	HSS C18	HSS C18	HSS C18
**54**	163222-33-1	ezetimibe	409.43	9.72	-0.2	3.96	4	2	$C_{24}H_{21}F_2NO_3$	HSS C18; CSH PFP	HSS C18; CSH PFP	HSS C18; CSH PFP
**55**	4602-84-0	farnesol	222.37	14.69	-	4.83	1	1	$C_{15}H_{26}O$	HSS C18; CSH PFP	HSS C18; CSH PFP	HSS C18; CSH PFP
**56**	29679-58-1	fenoprofen	242.27	4.2	-	3.72	3	1	$C_{15}H_{14}O_3$	CSH PFP; PGC	HSS C18; CSH PFP; PGC	HSS C18; CSH PFP; PGC
**57**	5104-49-4	flurbiprofen	244.26	4.14	-	3.66	2	1	$C_{15}H_{13}FO_2$	HSS C18; CSH PFP; PGC	HSS C18; CSH PFP; PGC	HSS C18; CSH PFP; PGC
**58**	93957-54-1	fluvastatin	411.47	4.27	-	4.57	5	3	$C_{24}H_{26}FNO_4$	CSH PFP	CSH PFP	CSH PFP
**59**	54-28-4	gamma-tocopherol	416.68	11.05	-	10.72	2	1	$C_{28}H_{48}O_2$	HSS C18; CSH PFP	HSS C18; CSH PFP	HSS C18; CSH PFP
**60**	14101-61-2	gamma-tocotrienol	410.63	11.05	-	9.46	2	1	$C_{28}H_{42}O_2$	HSS C18; CSH PFP	HSS C18; CSH PFP	HSS C18; CSH PFP
**61**	520-33-2	hesperetin	302.28	7.49	-	1.94	6	3	$C_{16}H_{16}O_6$	n/a	HSS C18	HSS C18
**62**	520-26-3	hesperidin	610.56	7.15	-	-1.21	15	8	$C_{28}H_{34}O_{15}$	PGC	PGC	PGC
**63**	4270-27-3	chlorouracil (4-Chloro-2,6-dihydroxypyrimidine)	146.53	6.24	-	0.03	4	2	$C_4H_3ClN_2O_2$	HSS C18; CSH PFP; PGC	HSS C18; CSH PFP; PGC	HSS C18; CSH PFP; PGC
**64**	15687-27-1	ibuprofen	206.28	4.41	-	3.50	2	1	$C_{13}H_{18}O_2$	PGC	HSS C18; PGC	PGC
**65**	50-49-7	imipramine	280.41	-	9.49	4.36	2	0	$C_{19}H_{24}N_2$	CSH PFP	HSS C18; CSH PFP	CSH PFP
**66**	1516864-05-3	atorvastatin impurity A	540.65	4.29	0.39	3.91	7	4	$C_{33}H_{36}N_2O_5$	CSH PFP	CSH PFP	HSS C18; CSH PFP

n.	CAS No.	analyte	Mw	pKa acid	pKa basic	log P	H acc.	H donor	molecular formula	MeOH	MeOH + 10 mM NH_3	MeOH + 2% H_2O
**67**	842103-12-2	atorvastatin impurity B	558.64	4.29	0.38	3.85	7	4	$C_{33}H_{35}FN_2O_5$	CSH PFP	CSH PFP	HSS C18; CSH PFP
**68**	693793-53-2	atorvastatin impurity C	576.63	4.29	0.37	3.78	7	4	$C_{33}H_{34}F_2N_2O_5$	CSH PFP	CSH PFP	HSS C18; CSH PFP
**69**	53-86-1	indomethacin	357.79	3.96	-	4.25	5	1	$C_{19}H_{16}ClNO_4$	HSS C18; CSH PFP	HSS C18; CSH PFP	HSS C18; CSH PFP
**70**	480-19-3	isorhamnetine	316.26	6.31	-	2.79	4	11	$C_{16}H_{12}O_7$	PGC	PGC	PGC
**71**	22071-15-4	ketoprofen	254.28	4.23	-	2.91	3	1	$C_{16}H_{14}O_3$	HSS C18; CSH PFP; PGC	HSS C18; CSH PFP; PGC	HSS C18; CSH PFP; PGC
**72**	36894-69-6	labetalol	328.41	8.21	9.3	2.72	5	5	$C_{19}H_{24}N_2O_3$	n/a	CSH PFP	n/a
**73**	125995-03-1	atorvastatin lactone	540.62	13.39	0.38	3.90	6	2	$C_{33}H_{33}FN_2O_4$	HSS C18; CSH PFP	HSS C18; CSH PFP	HSS C18; CSH PFP
**74**	1256388-51-8	ledipasvir	889	11.2	5.42	4.54	14	4	$C_{49}H_{54}F_2N_8O_6$	CSH PFP	HSS C18; CSH PFP	HSS C18; CSH PFP
**75**	75330-75-5	lovastatin	404.54	13.49	-	4.31	5	1	$C_{24}H_{36}O_5$	HSS C18; CSH PFP; PGC	HSS C18; CSH PFP; PGC	HSS C18; CSH PFP; PGC
**76**	491-70-3	luteolin	286.24	6.5	-	2.70	6	4	$C_{15}H_{10}O_6$	n/a	n/a	n/a
**77**	376348-65-1	maraviroc	513.67	14.8	10.24	5.30	6	1	$C_{29}H_{41}F_2N_5O$	CSH PFP	HSS C18; CSH PFP	CSH PFP
**78**	51384-51-1	metoprolol	267.36	13.89	9.43	1.63	4	2	$C_{15}H_{25}NO_3$	PGC	CSH PFP; PGC	PGC
**79**	480-41-1	naringenin	272.25	7.52	-	2.63	3	8	$C_{15}H_{12}O_5$	HSS C18; CSH PFP	HSS C18; CSH PFP	HSS C18; CSH PFP
**80**	10236-47-2	naringin	580.54	7.17	-	-0.20	8	22	$C_{27}H_{32}O_{14}$	HSS C18; CSH PFP; PGC	HSS C18; CSH PFP; PGC	HSS C18; CSH PFP; PGC
**81**	99-87-6	p-cymene	134.22	14	-	4.01	0	0	$C_{10}H_{14}$	HSS C18	HSS C18	HSS C18
**82**	60-81-1	phloridzin	436.41	7.15	-	-0.37	7	17	$C_{21}H_{24}O_{10}$	HSS C18; CSH PFP	HSS C18; CSH PFP	HSS C18; CSH PFP
**83**	13523-86-9	pindolol	248.32	13.94	9.54	1.68	4	3	$C_{14}H_{20}N_2O_2$	n/a	HSS C18; CSH PFP	n/a
**84**	147511-69-1	pitavastatin	421.46	4.24	4.68	1.92	3	8	$C_{25}H_{24}FNO_4$	HSS C18; CSH PFP	HSS C18; CSH PFP	HSS C18; CSH PFP
**85**	81093-37-0	pravastatin	424.53	4.31	-	2.21	4	11	$C_{23}H_{36}O_7$	HSS C18; CSH PFP	CSH PFP	HSS C18; CSH PFP
**86**	525-66-6	propranolol	259.34	13.84	9.5	2.90	2	5	$C_{16}H_{21}NO_2$	n/a	HSS C18; CSH PFP	n/a
**87**	501-36-0	resveratrol	228.24	9.22	-	3.02	3	3	$C_{14}H_{12}O_3$	HSS C18; CSH PFP	HSS C18; CSH PFP	HSS C18; CSH PFP
**88**	155213-67-5	ritonavir	720.94	11.47	2.51	2.33	11	4	$C_{37}H_{48}N_6O_5S_2$	HSS C18; CSH PFP	HSS C18; CSH PFP	HSS C18; CSH PFP

n.	CAS No.	analyte	Mw	pKa acid	pKa basic	log P	H acc.	H donor	molecular formula	MeOH	MeOH + 10 mM NH_3	MeOH + 2% H_2O
**89**	123441-03-2	S-rivastigmine	250.34	-	8.62	2.06	0	4	$C_{14}H_{22}N_2O_2$	PGC	HSS C18; CSH PFP; PGC	PGC
**90**	415973-05-6	R-rivastigmine	250.34	-	8.62	2.06	0	4	$C_{14}H_{22}N_2O_2$	PGC	HSS C18; CSH PFP; PGC	PGC
**91**	18559-94-9	salbutamol	239.31	9.99	9.62	0.69	4	8	$C_{13}H_{21}NO_3$	PGC	PGC	PGC
**92**	92-61-5	scopoletin	192.17	7.91	-	1.38	4	1	$C_{10}H_8O_4$	HSS C18; CSH PFP	HSS C18; CSH PFP	HSS C18; CSH PFP
**93**	138-59-0	shikimic acid	174.15	4.48	-	-2.22	5	4	$C_7H_{10}O_5$	HSS C18; CSH PFP; PGC	HSS C18; CSH PFP; PGC	HSS C18; CSH PFP; PGC
**94**	923604-59-5	simeprevir	749.94	4.47	3.01	6.10	12	2	$C_{38}H_{47}N_5O_7S_2$	HSS C18; CSH PFP	HSS C18; CSH PFP	HSS C18; CSH PFP
**95**	79902-63-9	simvastatin	418.57	13.49	-	4.72	5	1	$C_{25}H_{38}O_5$	HSS C18; CSH PFP; PGC	HSS C18; CSH PFP; PGC	HSS C18; CSH PFP; PGC
**96**	486460-32-6	sitagliptin	407.31	-	7.2	2.06	6	2	$C_{16}H_{15}F_6N_5O$	n/a	HSS C18; CSH PFP	n/a
**97**	1190307-88-0	sofosbuvir	529.45	9.39	-3.26	2.21	12	3	$C_{22}H_{29}FN_3O_9P$	HSS C18; CSH PFP	HSS C18; CSH PFP	HSS C18; CSH PFP
**98**	603-61-2	tamarixetin	316.26	6.31	-	2.67	7	4	$C_{16}H_{12}O_7$	n/a	n/a	n/a
**99**	611-40-5	tectoridin	464.4	6.1	-	0.54	11	6	$C_{22}H_{22}O_{11}$	CSH PFP	n/a	n/a
**100**	548-77-6	tectorigenin	300.26	6.49	-	2.84	3	9	$C_{16}H_{12}O_6$	CSH PFP	CSH PFP	CSH PFP
**101**	58-22-0	testosterone	288.43	15.06	-	3.18	1	3	$C_{19}H_{28}O_2$	HSS C18; CSH PFP	HSS C18; CSH PFP	HSS C18; CSH PFP
**102**	274693-27-5	ticagrelor	522.57	13.26	3.05	2.02	4	14	$C_{23}H_{28}F_2N_6O_4S$	HSS C18; CSH PFP	HSS C18; CSH PFP	HSS C18; CSH PFP
**103**	140-10-3	trans-cinnamic acid	148.16	4.34	-	1.21	2	1	$C_9H_8O_2$	CSH PFP	HSS C18; CSH PFP	CSH PFP
**104**	501-94-0	tyrosol	138.16	10.17	-	0.85	2	2	$C_8H_{10}O_2$	HSS C18; CSH PFP; PGC	HSS C18; CSH PFP; PGC	HSS C18; CSH PFP; PGC
**105**	66-22-8	uracil	112.09	8.95	-4.19	-1.04	4	2	$C_4H_4N_2O_2$	PGC	CSH PFP; PGC	CSH PFP; PGC
**106**	121-33-5	vanillin	152.15	7.78	-	1.21	3	1	$C_8H_8O_3$	HSS C18; CSH PFP; PGC	HSS C18; CSH PFP; PGC	HSS C18; CSH PFP; PGC
**107**	224785-90-4	vardenafil	488.6	9.86	7.15	3.64	10	1	$C_{23}H_{32}N_6O_4S$	CSH PFP	HSS C18; CSH PFP	CSH PFP

Table S2: Description of the molecular descriptors calculated by CDK Descriptor Calculator.

Type and Class of Molecular descriptors	Individual descriptors (abbreviation)	Meaning
**ALOGP**	ALogP	Ghose-Crippen LogKow (octanol-water coefficient)
Constitutional Descriptor	AlogP2	Ghose-Crippen octanol water coefficient squared
(atom additive logP and molar refractivity values; described by Ghose and Crippen)	AMR	Ghose-Crippen molar refractivity
**APol** ***Electronic Descriptor***	Apol	sum of the atomic polarizabilities (including implicit hydrogens)
**AcidicGroupContent** ***Constitutional Descriptor***	nAcid	number of acidic groups
**BCUT**	BCUTw-1l	nhigh (number of highest eigenvalue) lowest atom weighted BCUTS
Hybrid Descriptor	BCUTw-1h	nlow (number of lowest eigenvalue) highest atom weighted BCUTS
(eigenvalue-based descriptor noted for	BCUTc-1l	nhigh (number of highest eigenvalue) lowest partial charge weighted BCUTS
its utility in chemical diversity described	BCUTc-1h	nlow (number of lowest eigenvalue) highest partial charge weighted BCUTS
by Pearlman et al; a weighted version of	BCUTp-1l	nhigh (number of highest eigenvalue) lowest polarizability weighted BCUTS
the Burden matrix)	BCUTp-1h	nlow (number of lowest eigenvalue) highest polarizability weighted BCUTS
	PPSA-1	partial positive surface area; sum of surface area on positive parts of molecule
	PPSA-2	partial positive surface area * total positive charge on the molecule
	PPSA-3	charge weighted partial positive surface area
	PNSA-1	partial negative surface area; sum of surface area on negative parts of molecule
	PNSA-2	partial negative surface area * total negative charge on the molecule
	PNSA-3	charge weighted partial negative surface area
	DPSA-1	difference of PPSA-1 and PNSA-1
	DPSA-2	difference of FPSA-2 and PNSA-2
	DPSA-3	difference of PPSA-3 and PNSA-3
	FPSA-1	PPSA-1 / total molecular surface area
**CPSA**	FPSA-2	PPSA-2 / total molecular surface area
Electronic and Geometrical Descriptor	FPSA-3	PPSA-3 / total molecular surface area
(29 Charged Partial Surface Area	FNSA-1	PNSA-1 / total molecular surface area
Descriptors)	FNSA-2	PNSA-2 / total molecular surface area
	FNSA-3	PNSA-3 / total molecular surface area
	WPSA-1	PPSA-1 * total molecular surface area / 1000
	WPSA-2	PPSA-2 * total molecular surface area / 1000
	WPSA-3	PPSA-3 * total molecular surface area / 1000
	WNSA-1	PNSA-1 * total molecular surface area / 1000
	WNSA-2	PNSA-2 * total molecular surface area / 1000
	WNSA-3	PNSA-3 * total molecular surface area / 1000
	RPCG	relative positive charge; most positive charge / total positive charge
	RNCG	relative negative charge; most negative charge / total negative charge
	RPCS	relative positive charge surface area; most positive surface area * RPCG

Type and Class of Molecular descriptors	Individual descriptors (abbreviation)	Meaning
	RNCS	relative negative charge surface area; most negative surface area * RNCG
	THSA	sum of solvent accessible surface areas of atoms with absolute value of partial charges less than 0.2
	TPSA	sum of solvent accessible surface areas of atoms with absolute value of partial charges less than 0.2
	RHSA	THSA / total molecular surface area
	RPSA	TPSA / total molecular surface area
**WHIM** *Hybrid Descriptor* ***(Weighted Holistic Invariant Molecular descriptors; based on a number of atom weightings)***	Wlambda1.unity;	directional descriptor; related to molecular size
	Wlambda2.unity	directional descriptor; related to molecular size
	Wlambda3.unity	directional descriptor; related to molecular size
	Wnu1.unity	directional descriptor; related to molecular shape
	Wnu2.unity	directional descriptor; related to molecular shape
	Weta1.unity	directional descriptor; related to density of the atoms distribution
	Weta2.unity	directional descriptor; related to density of the atoms distribution
	Weta3.unity	directional descriptor; related to density of the atoms distribution
	WT.unity	non-directional descriptor; related to linear contributions to the total molecular dimension
	WA.unity	non-directional descriptor; related to quadratic contributions to the total molecular dimension
	WV.unity	non-directional descriptor; contains also the third-order term;
	WK.unity	non-directional descriptor; molecular shape
	WD.unity	non-directional descriptor; the total molecular density
**MDE** ***Topological Descriptor*** **(*Molecular Distance Edge Descriptors for C, O, and N*)**	MDEC-11	molecular distance edge between all primary carbons
	MDEC-12	molecular distance edge between all primary and secondary carbons
	MDEC-13	molecular distance edge between all primary and tertiary carbons
	MDEC-14	molecular distance edge between all primary and quaternary carbons
	MDEC-22	molecular distance edge between all secondary carbons
	MDEC-23	molecular distance edge between all secondary and tertiary carbons
	MDEC-24	molecular distance edge between all secondary and quaternary carbons
	MDEC-33	molecular distance edge between all tertiary carbons
	MDEC-34	molecular distance edge between all tertiary and quaternary carbons
	MDEC-44	molecular distance edge between all quaternary carbons
	MDEO-11	molecular distance edge between all primary oxygens
	MDEO-12	molecular distance edge between all primary and secondary oxygens
	MDEO-22	molecular distance edge between all secondary oxygens
	MDEN-12	molecular distance edge between all primary and secondary nitrogens
	MDEN-13	molecular distance edge between all primary and tertiary nitrogens
	MDEN-22	molecular distance edge between all secondary nitrogens
	MDEN-23	molecular distance edge between all secondary and tertiary nitrogens
	MDEN-33	molecular distance edge between all tertiary nitrogens
**AromaticAtomsCount**	naAromAtom	number of aromatic atoms in an atom container

Type and Class of Molecular descriptors	Individual descriptors (abbreviation)	Meaning
Constitutional Descriptor		
**AromaticBonsCount**<br>***Constitutional Descriptor***	nAromBond	number of aromatic atoms in an AtomContainer; based on the number of aromatic bounds
**AtomCount**<br>***Constitutional Descriptor***	nAtom	number of atoms of a certain element type
**AutocorrelationCharge**<br>***Topological Descriptor***<br>***(the Moreau-Broto autocorrelation descriptors using partial charges)***	ATSc1<br>ATSc2<br>ATSc3<br>ATSc4<br>ATSc5	ATS autocorrelation descriptor, weighted by charges<br>ATS autocorrelation descriptor, weighted by charges<br>ATS autocorrelation descriptor, weighted by charges<br>ATS autocorrelation descriptor, weighted by charges<br>ATS autocorrelation descriptor, weighted by charges
**AutocorrelationMass**<br>***Topological Descriptor***<br>***(the Moreau-Broto autocorrelation descriptors using atomic weight)***	ATSm1<br>ATSm2<br>ATSm3<br>ATSm4<br>ATSm5	ATS autocorrelation descriptor, weighted by scaled atomic mass<br>ATS autocorrelation descriptor, weighted by scaled atomic mass<br>ATS autocorrelation descriptor, weighted by scaled atomic mass<br>ATS autocorrelation descriptor, weighted by scaled atomic mass<br>ATS autocorrelation descriptor, weighted by scaled atomic mass
**AutocorrelationPolarizability**<br>***Topological Descriptor***<br>***(the Moreau-Broto autocorrelation descriptors using polarizability)***	ATSp1<br>ATSp2<br>ATSp3<br>ATSp4<br>ATSp5	ATS autocorrelation descriptor, weighted by polarizability<br>ATS autocorrelation descriptor, weighted by polarizability<br>ATS autocorrelation descriptor, weighted by polarizability<br>ATS autocorrelation descriptor, weighted by polarizability<br>ATS autocorrelation descriptor, weighted by polarizability
**BPol**<br>***Electronic Descriptor***	bpol	sum of the absolute value of the difference between atomic polarizabilities of all bonded atoms in the molecule (including implicit hydrogens)
**BasicGroupCount**<br>***Constitutional Descriptor***	nBase	number of basic groups
**BondCount**<br>***Constitutional Descriptor***	nBx	single value with name nBX where X can be s(single bond), d (double), t (triple), a (aromatic), “”(all)
**CarbonTypes**<br>***Topological Descriptor***<br>***(carbon connectivity in the terms of hybridization)***	C1SP1<br>C1SP2<br>C2SP2<br>C3SP2<br>C1SP3<br>C2SP3<br>C3SP3<br>C4SP3	triply bound carbon bound to one other carbon<br>triply bound carbon bound to two other carbons<br>doubly bound carbon bound to two other carbons<br>doubly bound carbon bound to three other carbons<br>singly bound carbon bound to one other carbon<br>singly bound carbon bound to two other carbons<br>singly bound carbon bound to three other carbons<br>singly bound carbon bound to four other carbons
**ChiChain**<br>***Topological Descriptor***<br>***(evaluates the Kier & Hall Chi chain indices of orders 3,4,5 and 6; type of chain)***	SCH-3<br>SCH-4<br>SCH-5<br>SCH-6<br>SCH-7<br>VCH-3	simple chain, order 3<br>simple chain, order 4<br>simple chain, order 5<br>simple chain, order 6<br>simple chain, order 7<br>valence chain, order 3

Type and Class of Molecular descriptors	Individual descriptors (abbreviation)	Meaning
	VCH-4	valence chain, order 4
	VCH-5	valence chain, order 5
	VCH-6	valence chain, order 6
	VCH-7	valence chain, order 7
**ChiCluster** *Topological Descriptor (evaluates the Kier & Hall Chi cluster indices of orders 3,4,5, and 6)*	SC-3	simple cluster, order 3
	SC-4	simple cluster, order 4
	SC-5	simple cluster, order 5
	SC-6	simple cluster, order 6
	VC-3	valence cluster, order 3
	VC-4	valence cluster, order 4
	VC-5	valence cluster, order 5
	VC-6	valence cluster, order 6
**ChiPathCluster** *Topological Descriptor (evaluates the Kier & Hall Chi path cluster indices of orders 4,5, and 6)*	SPC-4	simple path cluster, order 4
	SPC-5	simple path cluster, order 5
	SPC-6	simple path cluster, order 6
	VPC-4	valence path cluster, order 4
	VPC-5	valence path cluster, order 5
	VPC-6	valence path cluster, order 6
**ChiPath** *Topological Descriptor (evaluates the Kier & Hall Chi path indices of orders 0,1,2,3,4,5,6 and 7)*	SP-0	simple path, order 0
	SP-1	simple path, order 1
	SP-2	simple path, order 2
	SP-3	simple path, order 3
	SP-4	simple path, order 4
	SP-5	simple path, order 5
	SP-6	simple path, order 6
	SP-7	simple path, order 7
	VP-0	valence path, order 0
	VP-1	valence path, order 1
	VP-2	valence path, order 2
	VP-3	valence path, order 3
	VP-4	valence path, order 4
	VP-5	valence path, order 5
	VP-6	valence path, order 6
	VP-7	valence path, order 7
**EccentricConnectivityIndex** *Topological Descriptor*	ECCEN	combining distance and adjacency information
**FMF** *Topological Descriptor*	FMF	ratio of heavy atoms in the framework to the total number of heavy atoms in the molecule; characterize the complexity of the molecule
**FragmentComplexity** *Topological Descriptor*	fragC	Complexity of a system; C=abs(B^2-A^2+A)+H/100 where C=complexity, A=number of non-hydrogen atoms, B=number of bonds and H=number of heteroatoms

Type and Class of Molecular descriptors	Individual descriptors (abbreviation)	Meaning
**GravitationalIndex**<br>***Geometrical Descriptor***<br>***(mass distribution of the molecule)***	GRAV-1	gravitational index of heavy atoms
	GRAV-2	square root of gravitational index of heavy atoms
	GRAV-3	cube root of gravitational index of heavy atoms
	GRAVH-1	gravitational index - hydrogens included
	GRAVH-2	square root of hydrogen-included gravitational index
	GRAVH-3	cube root of hydrogen-included gravitational index
	GRAV-4	grav1 for all pairs of atoms (not just bonded pairs)
	GRAV-5	grav2 for all pairs of atoms (not just bonded pairs)
	GRAV-6	grav3 for all pairs of atoms (not just bonded pairs)
**HBondAcceptorCount**<br>***Electronic Descriptor***	nHBAcc	the number of H bond acceptors using a slightly simplified version of the PHACIR atom types.<br>The groups counted as acceptors: (i) Any O where the formal charge of the oxygen is non-positive (i.e. formal charge ≤ 0) except an aromatic ether O and an O that is adjacent to a N; (ii) Any N where the formal charge of the N is non-positive (i.e. formal charge ≤ 0) except a N that is adjacent to an O
**HBondDonorCount**<br>***Electronic Descriptor***	nHBDon	number of H bond donors using a slightly simplified version of the PHACIR atom types.<br>The groups counted as donors: (i) Any-OH where the formal charge of the O is non-negative (i.e. formal charge ≥ 0); (ii) Any-NH where the formal charge of the N is non-negative (i.e. formal charge ≥ 0)
**HybridizationRatio**<br>***Topologocial Descriptor***	HybRatio	the fraction of sp3 carbons to sp2 carbons; complexity of the molecule
**KierHallSmarts**<br>***Topological Descriptor***<br>***(it counts the number of occurrences of the E-state fragments;***<br>***- single bond; =double bond; # triple bond; : aromatic bond)***	khs.sCH3	count of atom-type E-state: $-CH_3$
	khs.ssCH2	count of atom-type E-state: $-CH_2-$
	khs.dsCH	count of atom-type E-state: =CH-
	khs.aaCH	count of atom-type E-state: :CH:
	khs.sssCH	count of atom-type E-state: >CH-
	khs.tsC	count of atom-type E-state: #C-
	khs.dssC	count of atom-type E-state: =C<
	khs.aasC	count of atom-type E-state: :C:-
	khs.aaaC	count of atom-type E-state: ::C:
	khs.ssssC	count of atom-type E-state: >C<
	khs.sNH2	count of atom-type E-state: $-NH_2$
	khs.ssNH	count of atom-type E-state: $-NH_2-^+$
	khs.aaNH	count of atom-type E-state: :NH:
	khs.tN	count of atom-type E-state: #N
	khs.aaN	count of atom-type E-state: :N:
	khs.sssN	count of atom-type E-state: >N-
	khs.aasN	count of atom-type E-state: :N:-
	khs.sOH	count of atom-type E-state: -OH
	khs.dO	count of atom-type E-state: =O

Type and Class of Molecular descriptors	Individual descriptors (abbreviation)	Meaning
	khs.ssO	count of atom-type E-state: -O-
	khs.aaO	count of atom-type E-state: :O:
	khs.sF	count of atom-type E-state: -F
	khs.dsssP	count of atom-type E-state: ->P=
	khs.dS	count of atom-type E-state: =S
	khs.ssS	count of atom-type E-state: -S-
	khs.aaS	count of atom-type E-state: :S:
	khs.ddssS	count of atom-type E-state: >S==
	khs.sCl	count of atom-type E-state: -Cl
	khs.sBr	count of atom-type E-state: -Br
**KappaShapeIndices**	Kier1	first kappa shape index
Topological Descriptor	Kier2	second kappa shape index
(Kier and Hall kappa molecular shape indices compare the molecular graph with minimal and maximal molecular graphs)	Kier3	third kappa shape index
	MOMI-X	moment of inertia along X axis
**MomentOfInertia**	MOMI-Y	moment of inertia along Y axis
Geometrical Descriptor	MOMI-Z	moment of inertia along Z axis
(principal moment of inertia, their ratios,	MOMI-XY	ratio X/Y
and radius of gyration; characterize the	MOMI-XZ	ratio X/Z
mass distribution of a molecule)	MOMI-YZ	ratio Y/Z
	MOMI-R	radius of the gyration of the molecule
**WeightedPath**	WTPT-1	molecular ID
Topological Descriptor	WTPT-2	molecular ID / number of atoms
(The weighted path (molecular ID)	WTPT-3	sum of path lengths starting from heteroatoms
descriptors described by Randic. They	WTPT-4	sum of path lengths starting from oxygens
characterize molecular branching.)	WTPT-5	sum of path lengths starting from nitrogens
**WienerNumbers**	WPATH	weiner path number
Topological Descriptor (described by Randic, characterize molecular branching)	WPOL	weiner polarity number
**LargestChain** ***Constitutional Descriptor***	nAtomLC	number of atoms in the largest chain
**LargestPiSystem** ***Constitutional Descriptor***	nAtomP	number of atoms in the largest pi system
**LongestAliphaticChain** ***Constitutional Descriptor***	nAtomLAC	number of atoms in the longest aliphatic chain
**PetitjeanNumber** ***Topological Descriptor***	PetitjeanNuber	the eccentricity of a vertex corresponds to the distance from that vertex to the most remote vertex in the graph. The distance is obtained from the distance matrix as the count

Type and Class of Molecular descriptors	Individual descriptors (abbreviation)	Meaning
		of edges between the two vertices. If r(i) is the largest matrix entry in row i of the distance matrix D, then the radius is defined as the smallest of the r(i). The graph diameter D is defined as the largest vertex eccentricity in the graph
**MannholdLogP** *Constitutional Descriptor*	MLogP	prediction of logP based on the number of carbon and hetero atoms
**PetitjeanShapeIndex** *Topological/Geometrical Descriptor (anisotropy of molecule)*	topoShape<br>geomShape	topological shape index<br>geometric shape index
**RuleOfFive** *Constitutional Descriptor*	LipinskiFailures	number failures of the Lipinski's Rule Of 5
**TPSA** *Topological/Electronic Descriptor*	TopoPSA	topological polar surface area based on fragment contributions
**VABC** *Constitutional Descriptor*	VABC	values derived from the van der Waals Volume as a Sum of Atomic and Bond Contributions (VABC)
**VAdjMa** *Topological Descriptor*	VAdjMat	Vertex adjacency information (magnitude): 1 + log2 m where m is the number of heavy-heavy bonds
**Weight** *Constitutional Descriptor*	MW	based on the weight of atoms of a certain element type
**XLogP** *Constitutional Descriptor*	XLogP	prediction of logP based on the atom-type method
**ZagrebIndex** *Topological Descriptor*	Zagreb	the sum of the squares of atom degree over all heavy atoms i
**RotatableBondsCount** *Constitutional Descriptor*	nRotB	number of rotatable bonds is given by the SMARTS specified by Daylight
Other Constitutional Descriptors	tpsaEfficiency<br>nSmallRings<br>nAromRings<br>nRingBlocks<br>nAromBlocks<br>nRings3<br>nRings4<br>nRings5<br>nRings6<br>nRings7	Polar surface area expressed as a ratio to molecular size<br>Number of small rings from size 3 to 9<br>Number of aromatic rings<br>Total number of distinct ring blocks<br>Total number of “aromatically connected components”<br>individual breakdown of small ring, size 3<br>individual breakdown of small ring, size 4<br>individual breakdown of small ring, size 5<br>individual breakdown of small ring, size 6<br>individual breakdown of small ring, size 7

Table S3: Molecular descriptors used in the study grouped by primary category. For the description of molecular descriptors see SI Table S2.

**primary group**	***parameter***
lipophilicity	**ALogP**
	**ALogp2**
	**MLogP**
	**XLogP**
molecular weight/size	**BCUTw-1l**
	**BCUTw-1h**
	**Wlambda1,unity**
	**Wlambda2,unity**
	**Wlambda3,unity**
	**WT,unity**
	**WA,unity**
	**WV,unity**
	**ATSm1**
	**ATSm2**
	**ATSm3**
	**ATSm4**
	**ATSm5**
	**MW**
polarizability	**AMR**
	**BCUTp-1l**
	**BCUTp-1h**
	**apol**
	**ATSp1**
	**ATSp2**
	**ATSp3**
	**ATSp4**
	**ATSp5**
	**bpol**
	**WPOL**
charge	**BCUTc-1l**
	**BCUTc-1h**
	**THSA**
	**TPSA**
	**RHSA**
	**RPSA**
	**ATSc1**
	**ATSc2**
	**ATSc3**
	**ATSc4**
	**ATSc5**
	**TopoPSA**

primary group	*parameter*
positive charge	**PPSA-1**
	**PPSA-2**
	**PPSA-3**
	**DPSA-1**
	**DPSA-2**
	**DPSA-3**
	**FPSA-1**
	**FPSA-2**
	**FPSA-3**
	**WPSA-1**
	**WPSA-2**
	**WPSA-3**
	**RPCG**
	**RPCS**
	**tpsaEfficiency**
negative charge	**PNSA-1**
	**PNSA-2**
	**PNSA-3**
	**FNSA-1**
	**FNSA-2**
	**FNSA-3**
	**WNSA-1**
	**WNSA-2**
	**WNSA-3**
	**RNCG**
	**RNCS**
carbon groups	**fragC**
	**C1SP1**
	**C1SP2**
	**C2SP2**
	**C3SP2**
	**C1SP3**
	**C2SP3**
	**C3SP3**
	**C4SP3**
	**HybRatio**
	**khs,sCH3**
	**khs,ssCH2**
	**khs,dsCH**
	**khs,sssCH**
	**khs,tsC**
	**khs,dssC**
	**khs,ssssC**

primary group	*parameter*
	nAtomLC
	nAtomLAC
	MDEC-11
	MDEC-12
	MDEC-13
	MDEC-14
	MDEC-22
	MDEC-23
	MDEC-24
	MDEC-33
	MDEC-34
	MDEC-44
molecular shape	Wnu1,unity
	Wnu2,unity
	WK,unity
	Kier1
	Kier2
	Kier3
	MOMI-X
	MOMI-Y
	MOMI-Z
	MOMI-XY
	MOMI-XZ
	MOMI-YZ
	MOMI-R
	geomShape
atom density	Weta1,unity
	Weta2,unity
	Weta3,unity
	WD,unity
acidity/basicity	nAcid
	nBase
	LipinskiFailures
	PetitjeanNumber
chi chain/valence electrons	SCH-3
	SCH-4
	SCH-5
	SCH-6
	SCH-7
	VCH-3
	VCH-4
	VCH-5
	VCH-6

**primary group**	***parameter***
	**VCH-7**
	**SC-3**
	**SC-4**
	**SC-5**
	**SC-6**
	**VC-3**
	**VC-4**
	**VC-5**
	**VC-6**
	**SP-0**
	**SP-1**
	**SP-2**
	**SP-3**
	**SP-4**
	**SP-5**
	**SP-6**
	**SP-7**
	**VP-0**
	**VP-1**
	**VP-2**
	**VP-3**
	**VP-4**
	**VP-5**
	**VP-6**
	**VP-7**
	**SPC-4**
	**SPC-5**
	**SPC-6**
	**VPC-4**
	**VPC-5**
	**VPC-6**
	**ECCEN**
	**WPATH**
cyclicity	**FMF**
	**topoShape**
gravitational index	**GRAV-1**
	**GRAV-2**
	**GRAV-3**
	**GRAVH-1**
	**GRAVH-2**
	**GRAVH-3**
	**GRAV-4**
	**GRAV-5**

primary group	*parameter*
	GRAV-6
H-bond	nHBDon
	nHBAcc
N groups	khs,sNH2
	khs,ssNH
	khs,tN
	khs,sssN
	MDEN-12
	MDEN-13
	MDEN-22
	MDEN-23
	MDEN-33
	WTPT-5
O groups	khs,sOH
	khs,dO
	khs,ssO
	MDEO-11
	MDEO-12
	MDEO-22
	WTPT-3
	WTPT-4
halogen groups	khs,sF
	khs,sCl
	khs,sBr
S groups	khs,dsssP
	khs,dS
	khs,ssS
	khs,ddssS
connectivity/bonds	nAtom
	nB
	nAtomP
	nRotB
	VAdjMat
	VABC
	WTPT-1
	WTPT-2
	Zagreb
aromates	naAromAtom
	nAromBond
	khs,aaCH
	khs,aasC
	khs,aaaC
	khs,aaNH

**primary group**	***parameter***
	**khs,aaN**
	**khs,aasN**
	**khs,aaO**
	**khs,aaS**
	**nSmallRings**
	**nAromRings**
	**nRingBlocks**
	**nAromBlocks**
	**nRings3**
	**nRings4**
	**nRings5**
	**nRings6**
	**nRings7**

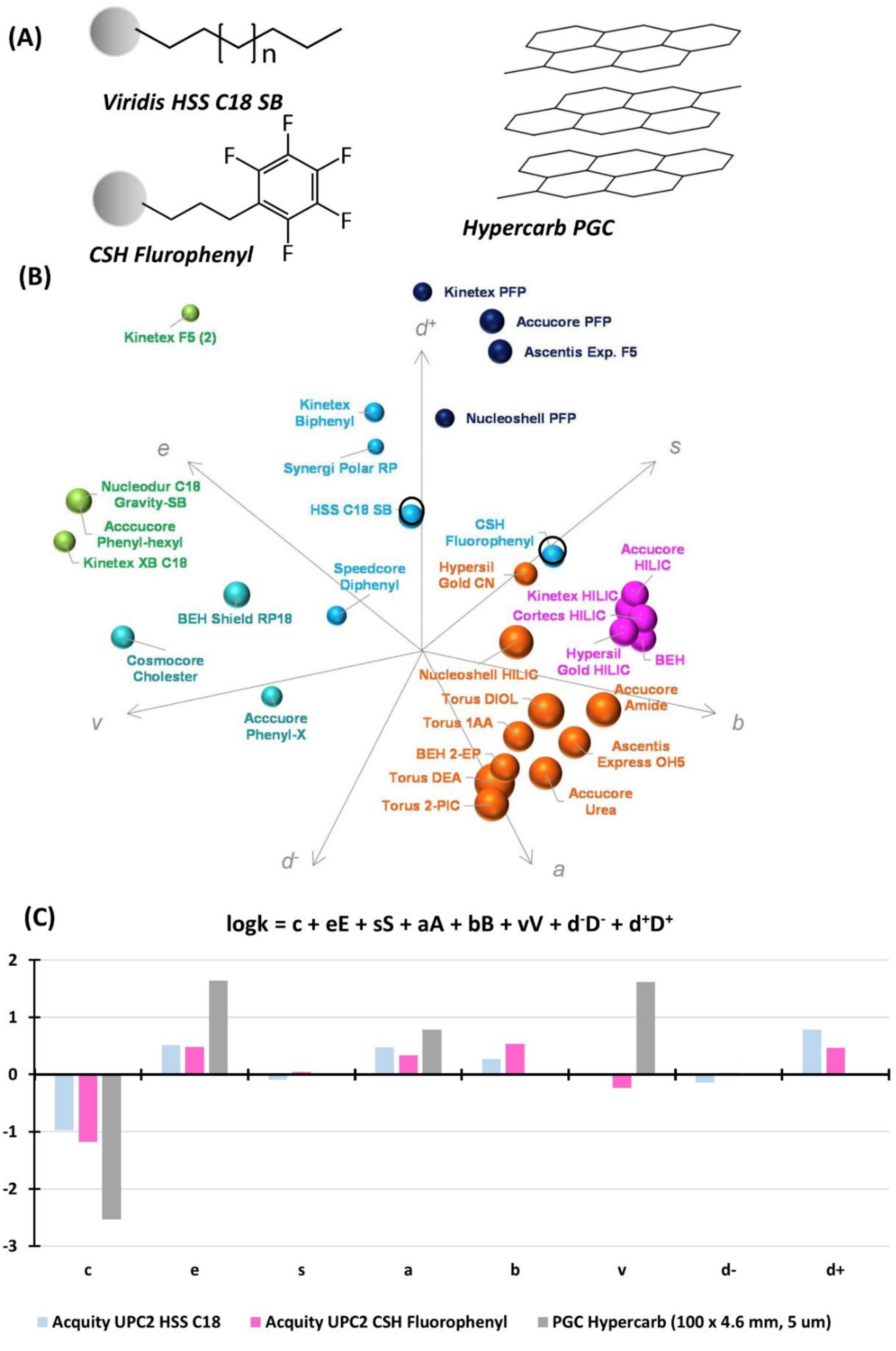

Figure S1. (A) The chemical structure of tested stationary phases with amine moieties, (B) the effect of LSER parameters on HSS C18 SB, CSH PFP, and PGC columns, and (C) a spider diagram characterizing stationary phases based on LSER characteristics. Black circles mark selected columns. The values of LSER parameters were taken from the article published by West et al.[1] The spider diagram was reprinted with permission from ref. 2.[2]

S2. Results

Table S4: Compounds eluting from HSS C18 SB column only using $MeOH+NH_3$ but not using MeOH and/or $MeOH+H_2O$.

compound	pK_a	pK_b
4-methoxy benzoic acid	4.47	-
abiraterone acetate	5.31	-
atomoxetine	10.15	-
bopindolol	17.59	9.4
cetirizin	3.46	6.71
cytosine	9	4.18
daclatasvir	10.92	6.56
darifenacin	15.7	9.32
desipramin	-	10.4
fenoprofen	4.2	-
hesperetin	7.49	-
ibuprofen	4.41	-
imipramine	-	9.49
ledipasvir	11.2	5.42
maraviroc	14.8	10.24
pindolol	13.94	9.54
propranolol	13.84	9.5
rivastigmin	-	8.62
rivastigmin isomer	-	8.62
sitagliptin	-	7.2
trans-cinnamic acid	4.34	-
vardenafil	9.86	7.15

Table S5: Compounds eluting from the CSH PFP column which were not possible to analyze on the HSS C18 SB column.

compounds eluting on CHS PFP but not on C18	pK_a	pK_b
(+)-thalidomide	10.7	-2.55
4-methoxy benzoic acid	4.47	-
abiraterone acetate	5.31	-
atorvastatin	4.29	0.38
daclatasvir	10.92	6.56
fenoprofen	4.2	-
fluvastatin	4.27	-
imipramine	-	9.49
Imp A	4.29	0.39
Imp B	4.29	0.38
Imp C	4.29	0.37
ledipasvir	11.2	5.42
maraviroc	14.8	10.24
pravastatin	4.31	-
shikimic acid	4.48	-
tectorigenin	6.49	-
trans-cinnamic acid	4.34	-
vardenafil	9.86	7.15
**compounds eluting on CSH PFP only using MeOH+NH_3**	**pK_a**	**pK_b**
2,3-dihydroxy benzoic acid	2.96	-
2,4-dihydroxy benzoic acid	3.32	-
2,5-dihydroxy benzoic acid	3.01	-
2,6-dihydroxy benzoic acid	1.3	-
2-hydroxy cinnamic acid	4.51	-
acebutolol	13.78	9.4
atenolol	13.88	9.43
atomoxetine	10.15	-
beta tocopherol	11.05	-
beta tocotrienol	11.05	-
bopindolol	17.59	9.4
cetirizin	3.46	6.71
cytosine	9	4.18
darifenacin	15.7	9.32
dasatinib	10.94	7.29
labetalol	8.21	9.3
metoprolol	13.89	9.43
pindolol	13.94	9.54
propranolol	13.84	9.5
rivastigmin	-	8.62
rivastigmin isomer	-	8.62
sitagliptin	-	7.2
uracil	8.95	-4.19

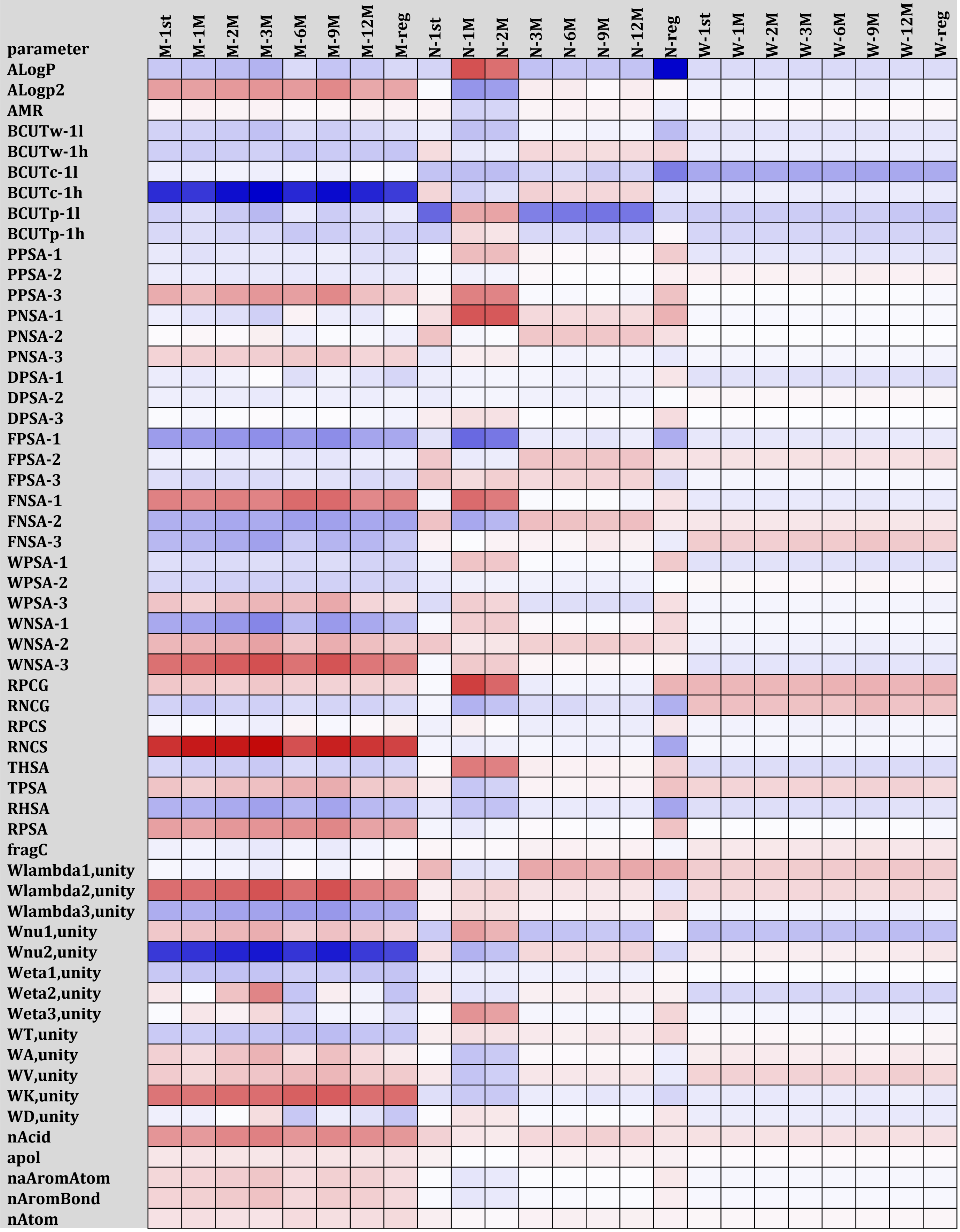
parameter
M-1st
M-1M
M-2M
M-3M
M-6M
M-9M
M-12M
M-reg
N-1st
N-1M
N-2M
N-3M
N-6M
N-9M
N-12M
N-reg
W-1st
W-1M
W-2M
W-3M
W-6M
W-9M
W-12M
W-reg
ALogP
ALogp2
AMR
BCUTw-1l
BCUTw-1h
BCUTc-1l
BCUTc-1h
BCUTp-1l
BCUTp-1h
PPSA-1
PPSA-2
PPSA-3
PNSA-1
PNSA-2
PNSA-3
DPSA-1
DPSA-2
DPSA-3
FPSA-1
FPSA-2
FPSA-3
FNSA-1
FNSA-2
FNSA-3
WPSA-1
WPSA-2
WPSA-3
WNSA-1
WNSA-2
WNSA-3
RPCG
RNCG
RPCS
RNCS
THSA
TPSA
RHSA
RPSA
fragC
Wlambda1,unity
Wlambda2,unity
Wlambda3,unity
Wnu1,unity
Wnu2,unity
Weta1,unity
Weta2,unity
Weta3,unity
WT,unity
WA,unity
WV,unity
WK,unity
WD,unity
nAcid
apol
naAromAtom
nAromBond
nAtom

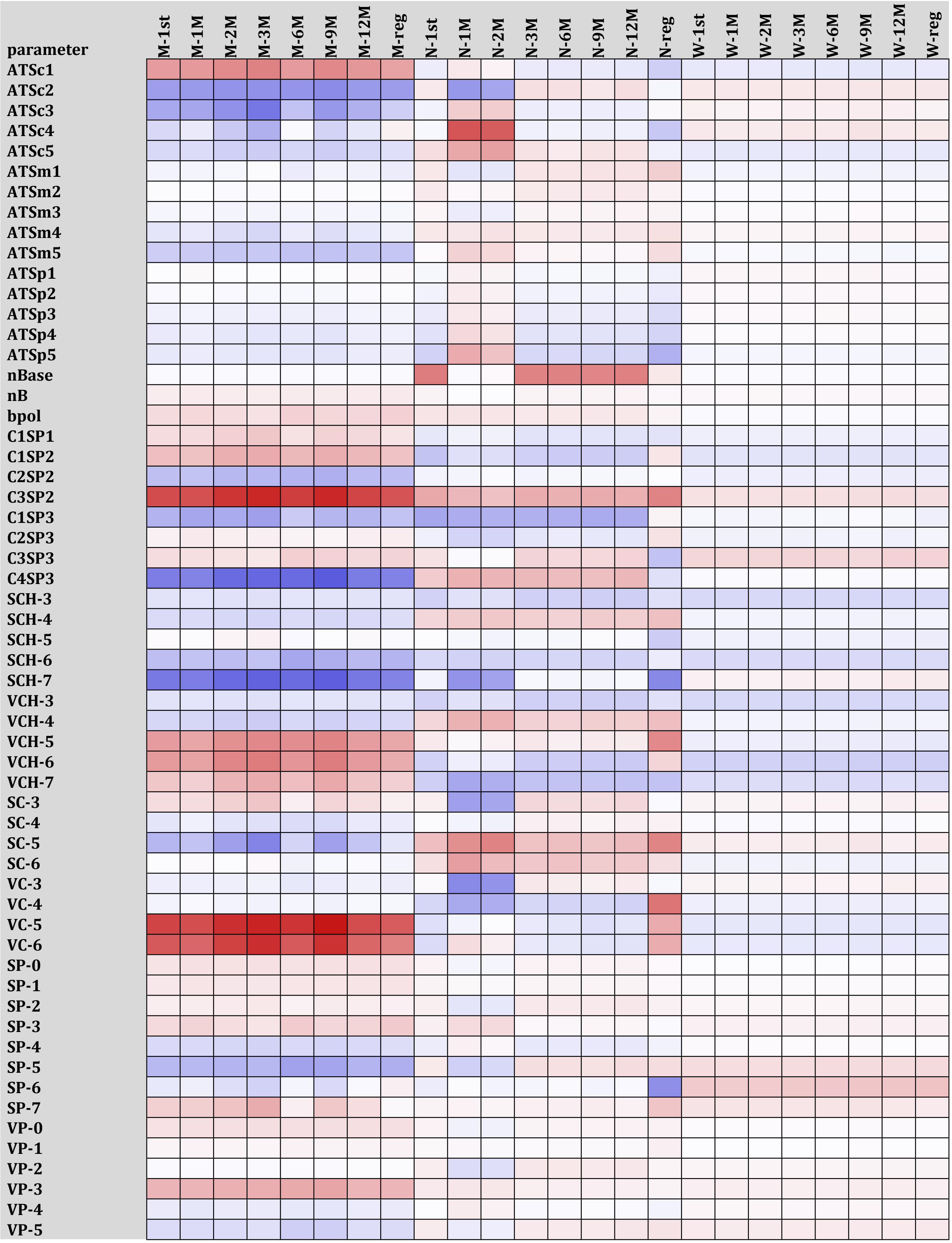
parameter
M-1st
M-1M
M-2M
M-3M
M-6M
M-9M
M-12M
M-reg
N-1st
N-1M
N-2M
N-3M
N-6M
N-9M
N-12M
N-reg
W-1st
W-1M
W-2M
W-3M
W-6M
W-9M
W-12M
W-reg
ATSc1
ATSc2
ATSc3
ATSc4
ATSc5
ATSm1
ATSm2
ATSm3
ATSm4
ATSm5
ATSp1
ATSp2
ATSp3
ATSp4
ATSp5
nBase
nB
bpol
C1SP1
C1SP2
C2SP2
C3SP2
C1SP3
C2SP3
C3SP3
C4SP3
SCH-3
SCH-4
SCH-5
SCH-6
SCH-7
VCH-3
VCH-4
VCH-5
VCH-6
VCH-7
SC-3
SC-4
SC-5
SC-6
VC-3
VC-4
VC-5
VC-6
SP-0
SP-1
SP-2
SP-3
SP-4
SP-5
SP-6
SP-7
VP-0
VP-1
VP-2
VP-3
VP-4
VP-5

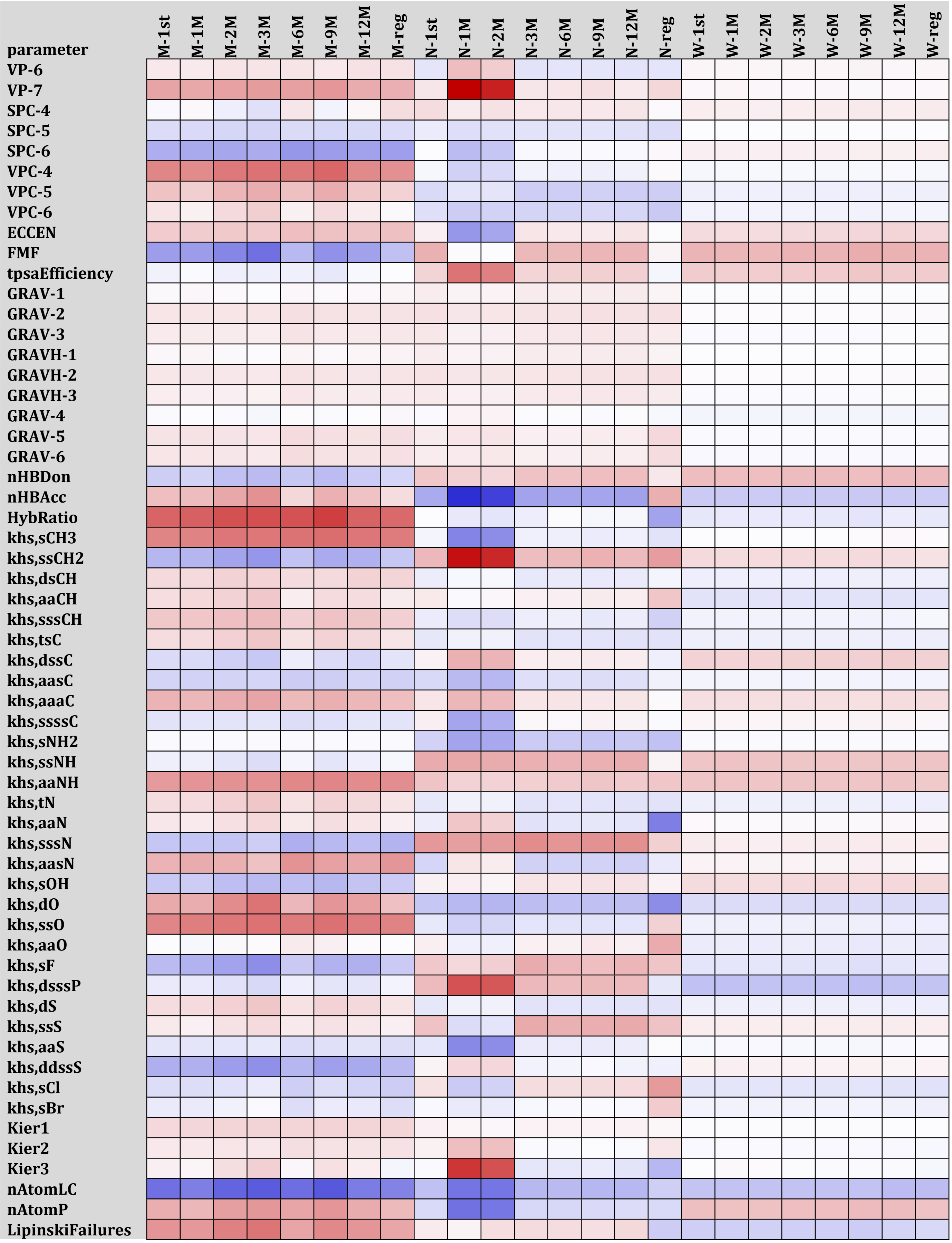
parameter
M-1st
M-1M
M-2M
M-3M
M-6M
M-9M
M-12M
M-reg
N-1st
N-1M
N-2M
N-3M
N-6M
N-9M
N-12M
N-reg
W-1st
W-1M
W-2M
W-3M
W-6M
W-9M
W-12M
W-reg
VP-6
VP-7
SPC-4
SPC-5
SPC-6
VPC-4
VPC-5
VPC-6
ECCEN
FMF
tpsaEfficiency
GRAV-1
GRAV-2
GRAV-3
GRAVH-1
GRAVH-2
GRAVH-3
GRAV-4
GRAV-5
GRAV-6
nHBDon
nHBAcc
HybRatio
khs,sCH3
khs,ssCH2
khs,dsCH
khs,aaCH
khs,sssCH
khs,tsC
khs,dssC
khs,aasC
khs,aaaC
khs,ssssC
khs,sNH2
khs,ssNH
khs,aaNH
khs,tN
khs,aaN
khs,sssN
khs,aasN
khs,sOH
khs,dO
khs,ssO
khs,aaO
khs,sF
khs,dsssP
khs,dS
khs,ssS
khs,aaS
khs,ddssS
khs,sCl
khs,sBr
Kier1
Kier2
Kier3
nAtomLC
nAtomP
LipinskiFailures

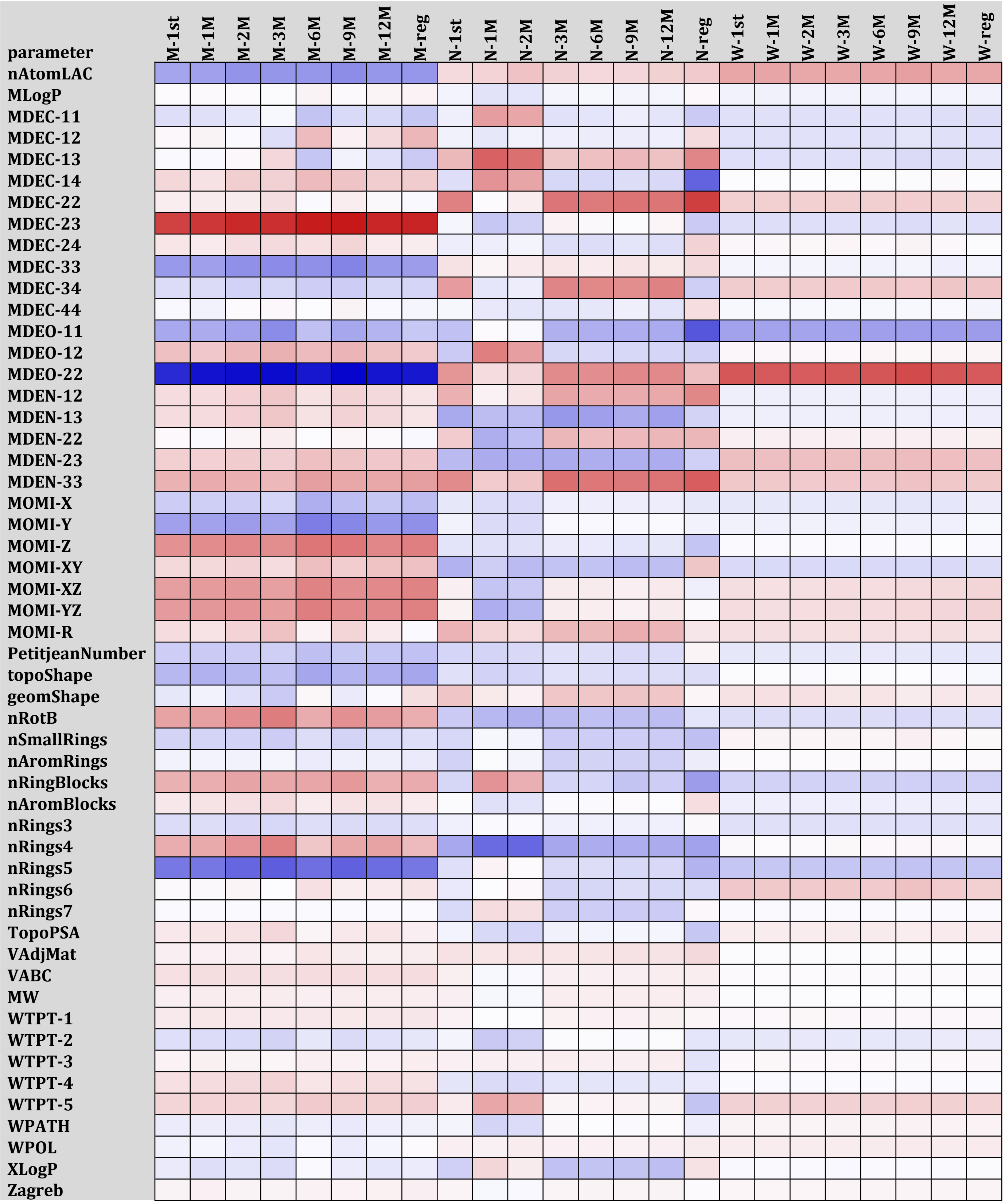

Figure S2. Heatmap of molecular descriptor weights representing their effect on the retention on HSS C18 SB column as determined by ANN. Blue – decreasing retention, red – increasing retention. M – MeOH as organic modifier, N – MeOH+10 mmol/L NH_3 as organic modifier, W- MeOH+2% H_2O as organic modifier. Data calculated based on the first injection (1st), or after 1 month of use (1M), 2 months (2M), 3 months (3M), 6 months (6M), 9 months (9M), 12 months (12M), or after the regeneration procedure (reg).

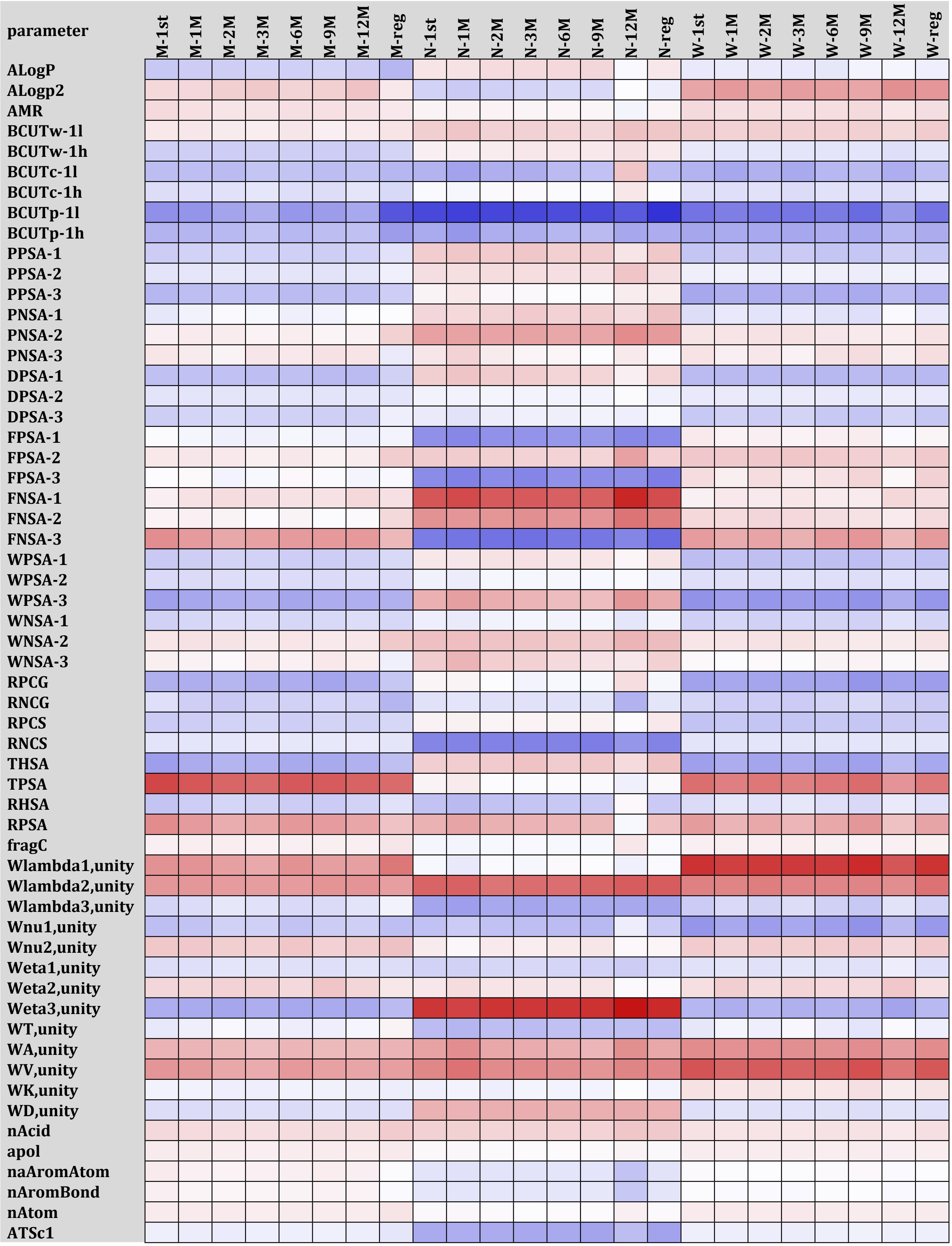
parameter
M-1st
M-1M
M-2M
M-3M
M-6M
M-9M
M-12M
M-reg
N-1st
N-1M
N-2M
N-3M
N-6M
N-9M
N-12M
N-reg
W-1st
W-1M
W-2M
W-3M
W-6M
W-9M
W-12M
W-reg
ALogP
ALogp2
AMR
BCUTw-1l
BCUTw-1h
BCUTc-1l
BCUTc-1h
BCUTp-1l
BCUTp-1h
PPSA-1
PPSA-2
PPSA-3
PNSA-1
PNSA-2
PNSA-3
DPSA-1
DPSA-2
DPSA-3
FPSA-1
FPSA-2
FPSA-3
FNSA-1
FNSA-2
FNSA-3
WPSA-1
WPSA-2
WPSA-3
WNSA-1
WNSA-2
WNSA-3
RPCG
RNCG
RPCS
RNCS
THSA
TPSA
RHSA
RPSA
fragC
Wlambda1,unity
Wlambda2,unity
Wlambda3,unity
Wnu1,unity
Wnu2,unity
Weta1,unity
Weta2,unity
Weta3,unity
WT,unity
WA,unity
WV,unity
WK,unity
WD,unity
nAcid
apol
naAromAtom
nAromBond
nAtom
ATSc1

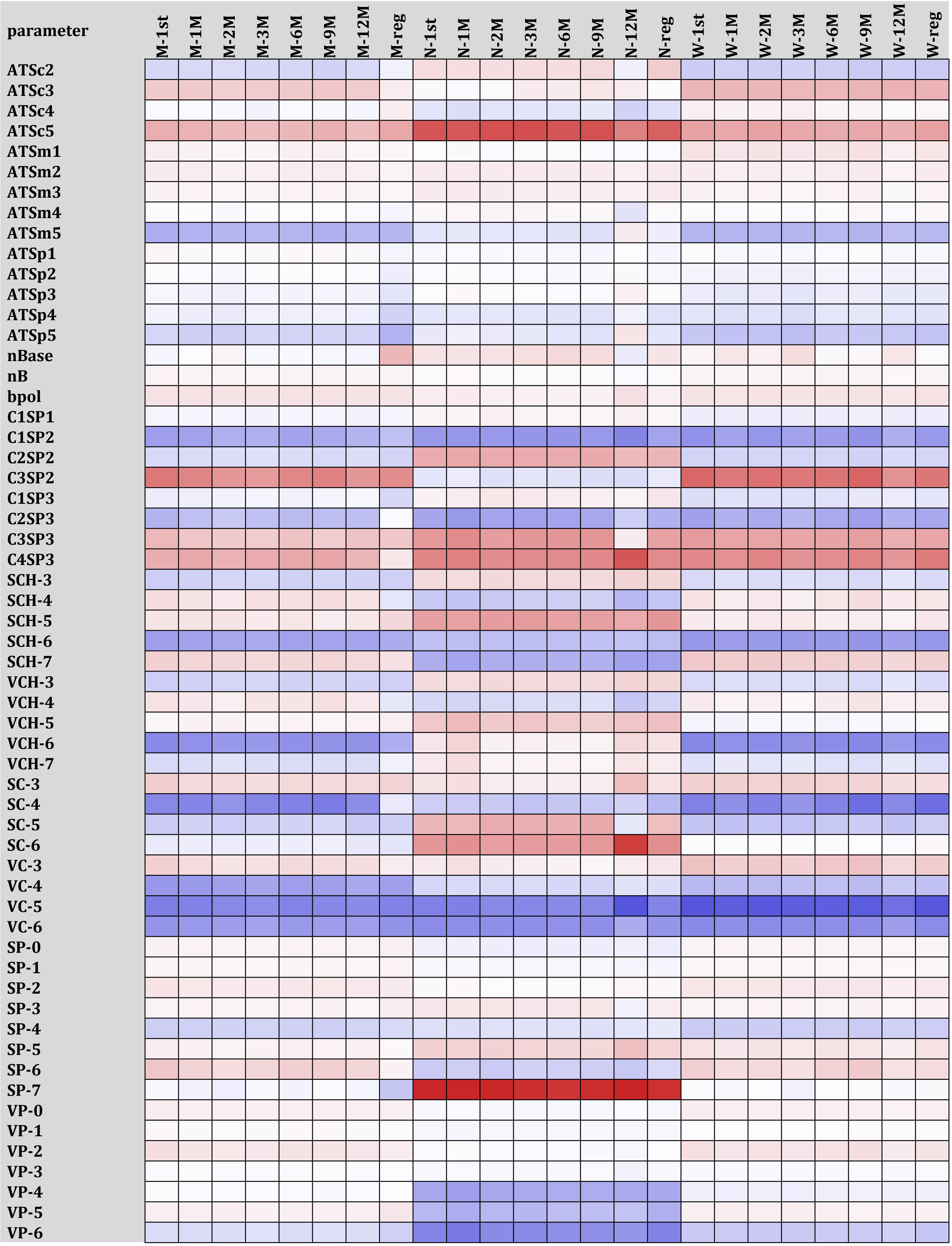
parameter
M-1st
M-1M
M-2M
M-3M
M-6M
M-9M
M-12M
M-reg
N-1st
N-1M
N-2M
N-3M
N-6M
N-9M
N-12M
N-reg
W-1st
W-1M
W-2M
W-3M
W-6M
W-9M
W-12M
W-reg
ATSc2
ATSc3
ATSc4
ATSc5
ATSm1
ATSm2
ATSm3
ATSm4
ATSm5
ATSp1
ATSp2
ATSp3
ATSp4
ATSp5
nBase
nB
bpol
C1SP1
C1SP2
C2SP2
C3SP2
C1SP3
C2SP3
C3SP3
C4SP3
SCH-3
SCH-4
SCH-5
SCH-6
SCH-7
VCH-3
VCH-4
VCH-5
VCH-6
VCH-7
SC-3
SC-4
SC-5
SC-6
VC-3
VC-4
VC-5
VC-6
SP-0
SP-1
SP-2
SP-3
SP-4
SP-5
SP-6
SP-7
VP-0
VP-1
VP-2
VP-3
VP-4
VP-5
VP-6

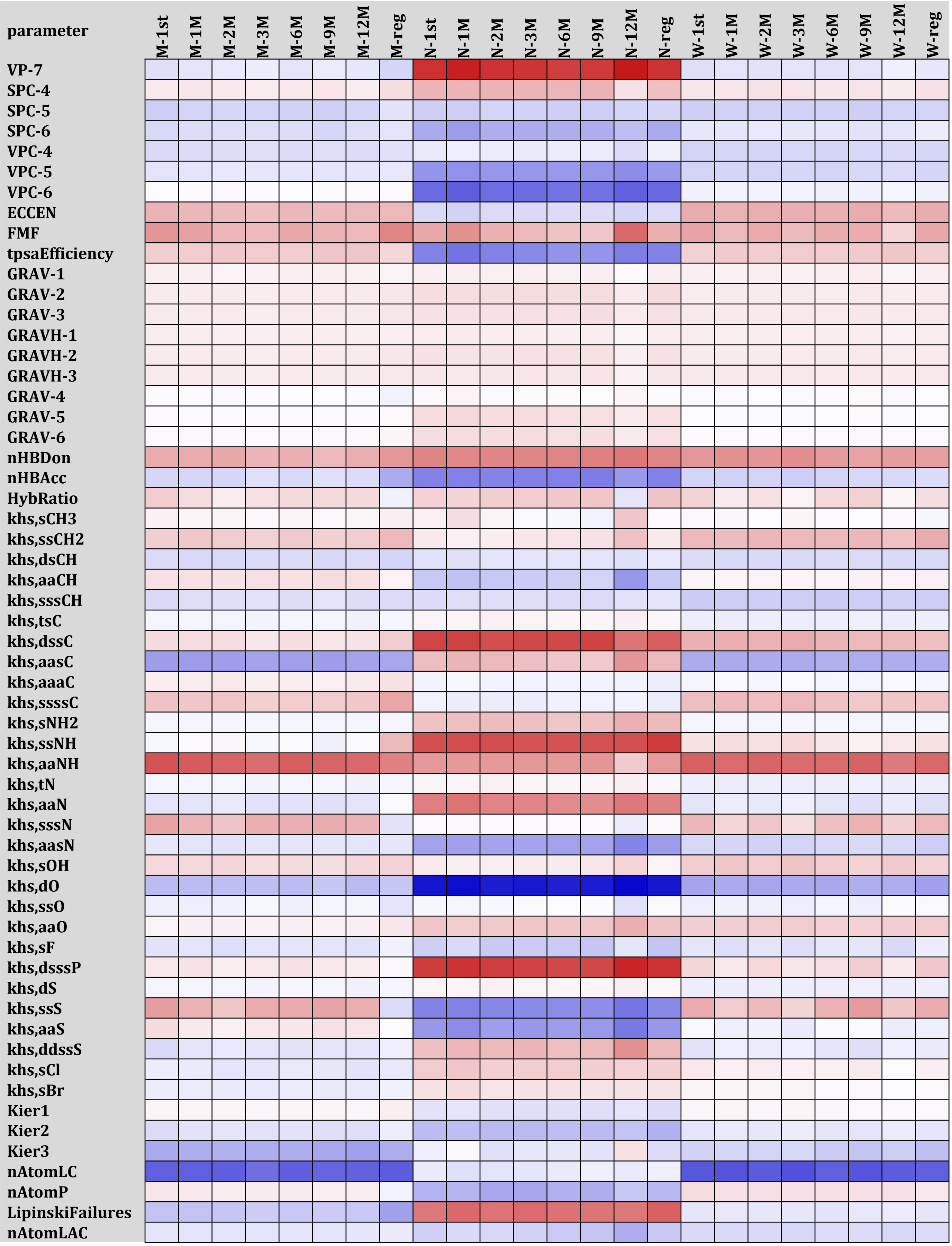
parameter
M-1st
M-1M
M-2M
M-3M
M-6M
M-9M
M-12M
M-reg
N-1st
N-1M
N-2M
N-3M
N-6M
N-9M
N-12M
N-reg
W-1st
W-1M
W-2M
W-3M
W-6M
W-9M
W-12M
W-reg
VP-7
SPC-4
SPC-5
SPC-6
VPC-4
VPC-5
VPC-6
ECCEN
FMF
tpsaEfficiency
GRAV-1
GRAV-2
GRAV-3
GRAVH-1
GRAVH-2
GRAVH-3
GRAV-4
GRAV-5
GRAV-6
nHBDon
nHBAcc
HybRatio
khs,sCH3
khs,ssCH2
khs,dsCH
khs,aaCH
khs,sssCH
khs,tsC
khs,dssC
khs,aasC
khs,aaaC
khs,ssssC
khs,sNH2
khs,ssNH
khs,aaNH
khs,tN
khs,aaN
khs,sssN
khs,aasN
khs,sOH
khs,dO
khs,ssO
khs,aaO
khs,sF
khs,dsssP
khs,dS
khs,ssS
khs,aaS
khs,ddssS
khs,sCl
khs,sBr
Kier1
Kier2
Kier3
nAtomLC
nAtomP
LipinskiFailures
nAtomLAC

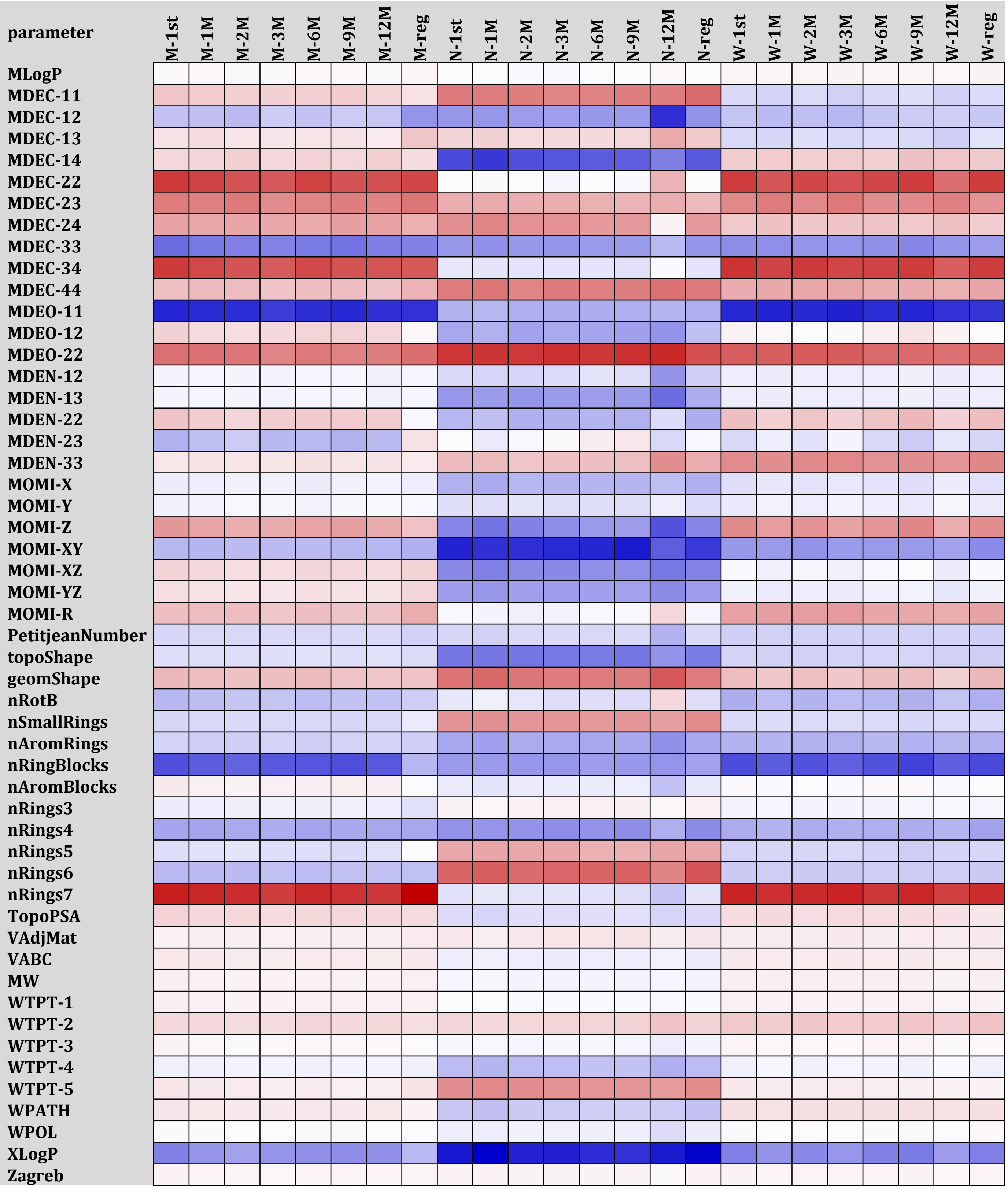

Figure S3. Heatmap of molecular descriptor weights representing their effect on the retention on CSH PFP column as determined by ANN. Blue – decreasing retention, red – increasing retention. M – MeOH as organic modifier, N – MeOH+10 mmol/L NH_3 as organic modifier, W- MeOH+2% H_2O as organic modifier . Data calculated based on the first injection (1st), or after 1 month of use (1M), 2 months (2M), 3 months (3M), 6 months (6M), 9 months (9M), 12 months (12M), or after the regeneration procedure (reg).

parameter	M-1st	M-1M	M-2M	M-3M	M-6M	M-9M	M-12M	M-reg	N-1st	N-1M	N-2M	N-3M	N-6M	N-9M	N-12M	N-reg	W-1st	W-1M	W-2M	W-3M	W-6M	W-9M	W-12M	W-reg
ALogP																								
ALogp2																								
AMR																								
BCUTw-1l																								
BCUTw-1h																								
BCUTc-1l																								
BCUTc-1h																								
BCUTp-1l																								
BCUTp-1h																								
PPSA-1																								
PPSA-2																								
PPSA-3																								
PNSA-1																								
PNSA-2																								
PNSA-3																								
DPSA-1																								
DPSA-2																								
DPSA-3																								
FPSA-1																								
FPSA-2																								
FPSA-3																								
FNSA-1																								
FNSA-2																								
FNSA-3																								
WPSA-1																								
WPSA-2																								
WPSA-3																								
WNSA-1																								
WNSA-2																								
WNSA-3																								
RPCG																								
RNCG																								
RPCS																								
RNCS																								
THSA																								
TPSA																								
RHSA																								
RPSA																								
fragC																								
Wlambda1,unity																								
Wlambda2,unity																								
Wlambda3,unity																								
Wnu1,unity																								
Wnu2,unity																								
Weta1,unity																								
Weta2,unity																								
Weta3,unity																								
WT,unity																								
WA,unity																								
WV,unity																								
WK,unity																								
WD,unity																								
nAcid																								
apol																								
naAromAtom																								
nAromBond																								
nAtom																								
ATSc1																								

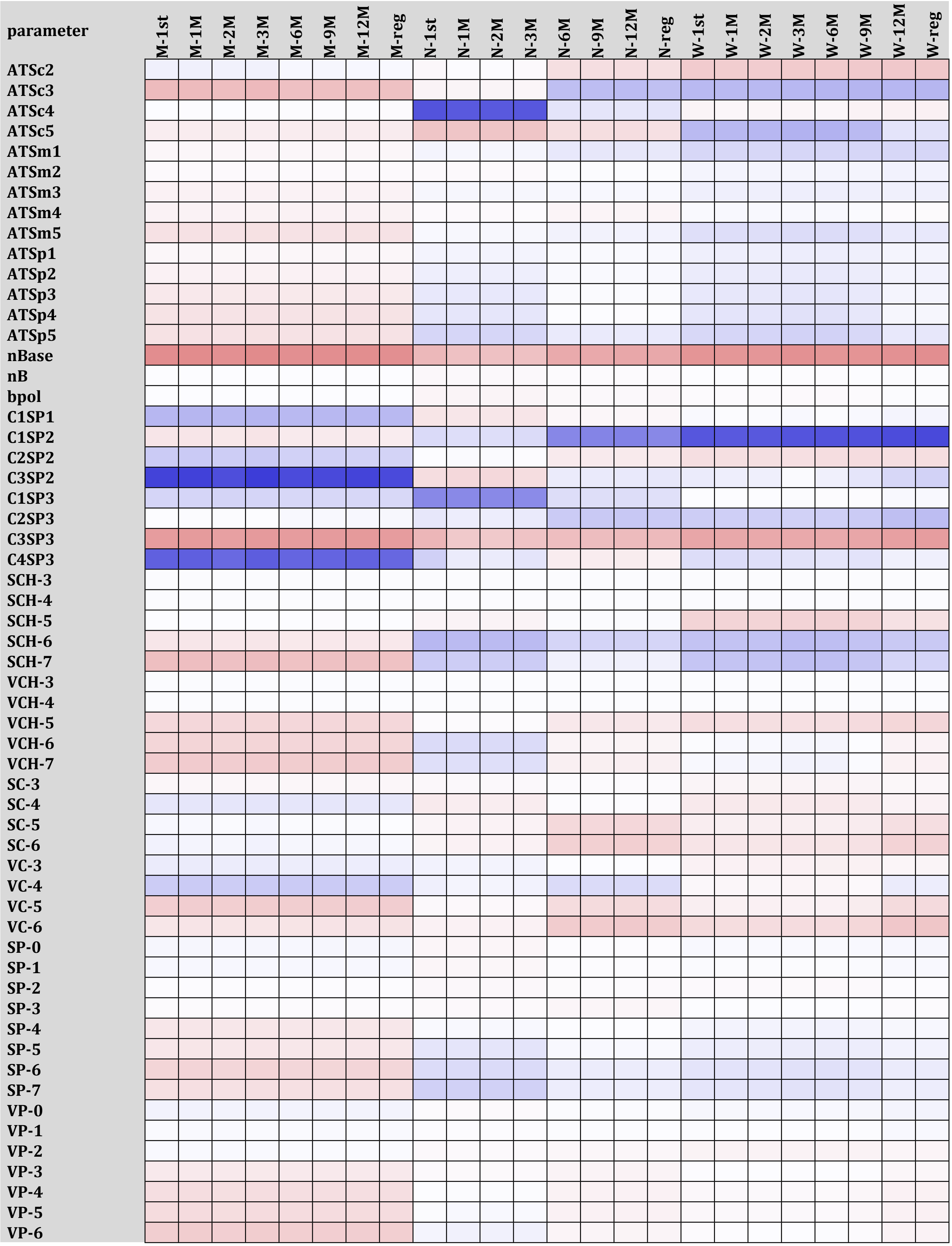
parameter
M-1st
M-1M
M-2M
M-3M
M-6M
M-9M
M-12M
M-reg
N-1st
N-1M
N-2M
N-3M
N-6M
N-9M
N-12M
N-reg
W-1st
W-1M
W-2M
W-3M
W-6M
W-9M
W-12M
W-reg
ATSc2
ATSc3
ATSc4
ATSc5
ATSm1
ATSm2
ATSm3
ATSm4
ATSm5
ATSp1
ATSp2
ATSp3
ATSp4
ATSp5
nBase
nB
bpol
C1SP1
C1SP2
C2SP2
C3SP2
C1SP3
C2SP3
C3SP3
C4SP3
SCH-3
SCH-4
SCH-5
SCH-6
SCH-7
VCH-3
VCH-4
VCH-5
VCH-6
VCH-7
SC-3
SC-4
SC-5
SC-6
VC-3
VC-4
VC-5
VC-6
SP-0
SP-1
SP-2
SP-3
SP-4
SP-5
SP-6
SP-7
VP-0
VP-1
VP-2
VP-3
VP-4
VP-5
VP-6

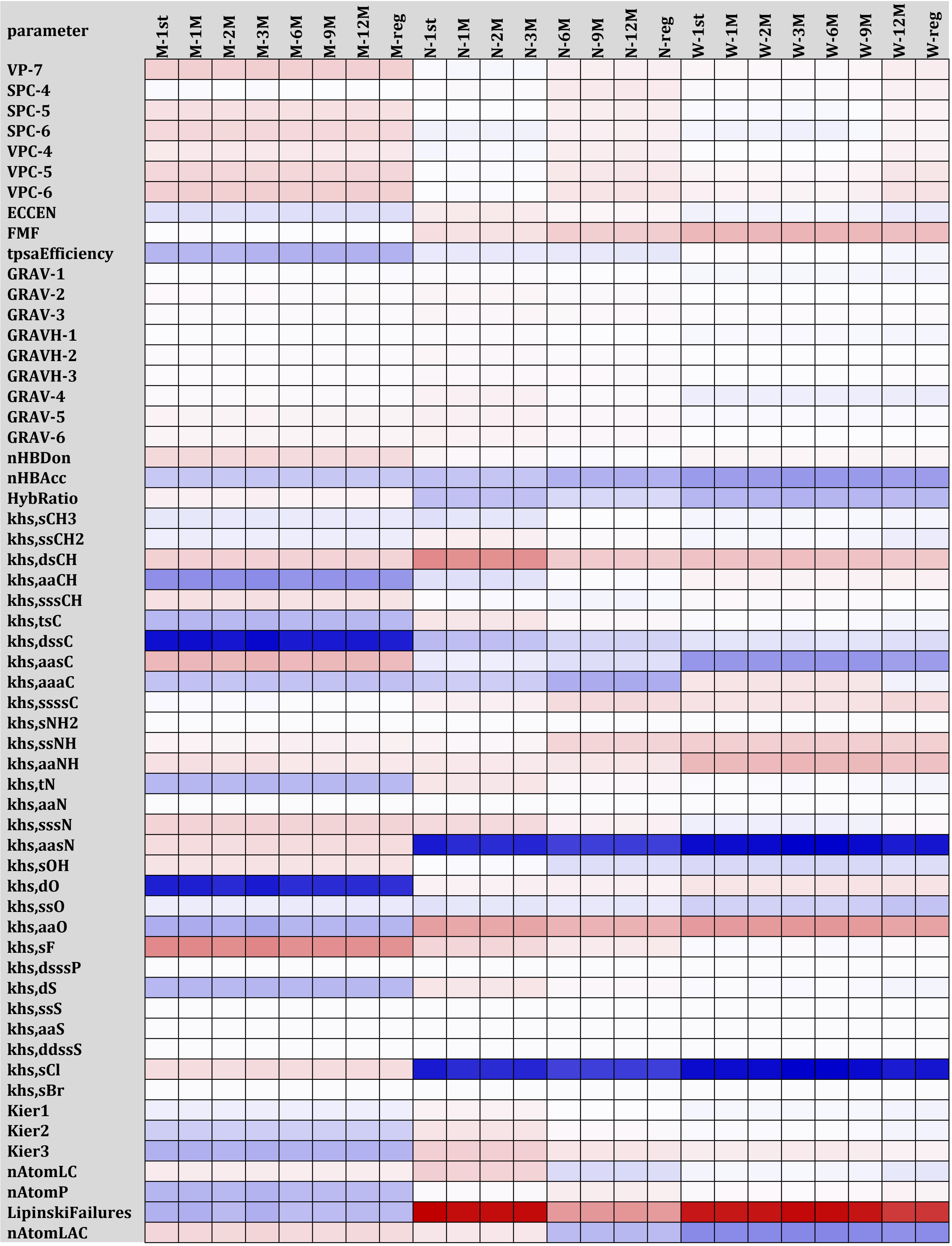
parameter
M-1st
M-1M
M-2M
M-3M
M-6M
M-9M
M-12M
M-reg
N-1st
N-1M
N-2M
N-3M
N-6M
N-9M
N-12M
N-reg
W-1st
W-1M
W-2M
W-3M
W-6M
W-9M
W-12M
W-reg
VP-7
SPC-4
SPC-5
SPC-6
VPC-4
VPC-5
VPC-6
ECCEN
FMF
tpsaEfficiency
GRAV-1
GRAV-2
GRAV-3
GRAVH-1
GRAVH-2
GRAVH-3
GRAV-4
GRAV-5
GRAV-6
nHBDon
nHBAcc
HybRatio
khs,sCH3
khs,ssCH2
khs,dsCH
khs,aaCH
khs,sssCH
khs,tsC
khs,dssC
khs,aasC
khs,aaaC
khs,ssssC
khs,sNH2
khs,ssNH
khs,aaNH
khs,tN
khs,aaN
khs,sssN
khs,aasN
khs,sOH
khs,dO
khs,ssO
khs,aaO
khs,sF
khs,dsssP
khs,dS
khs,ssS
khs,aaS
khs,ddssS
khs,sCl
khs,sBr
Kier1
Kier2
Kier3
nAtomLC
nAtomP
LipinskiFailures
nAtomLAC

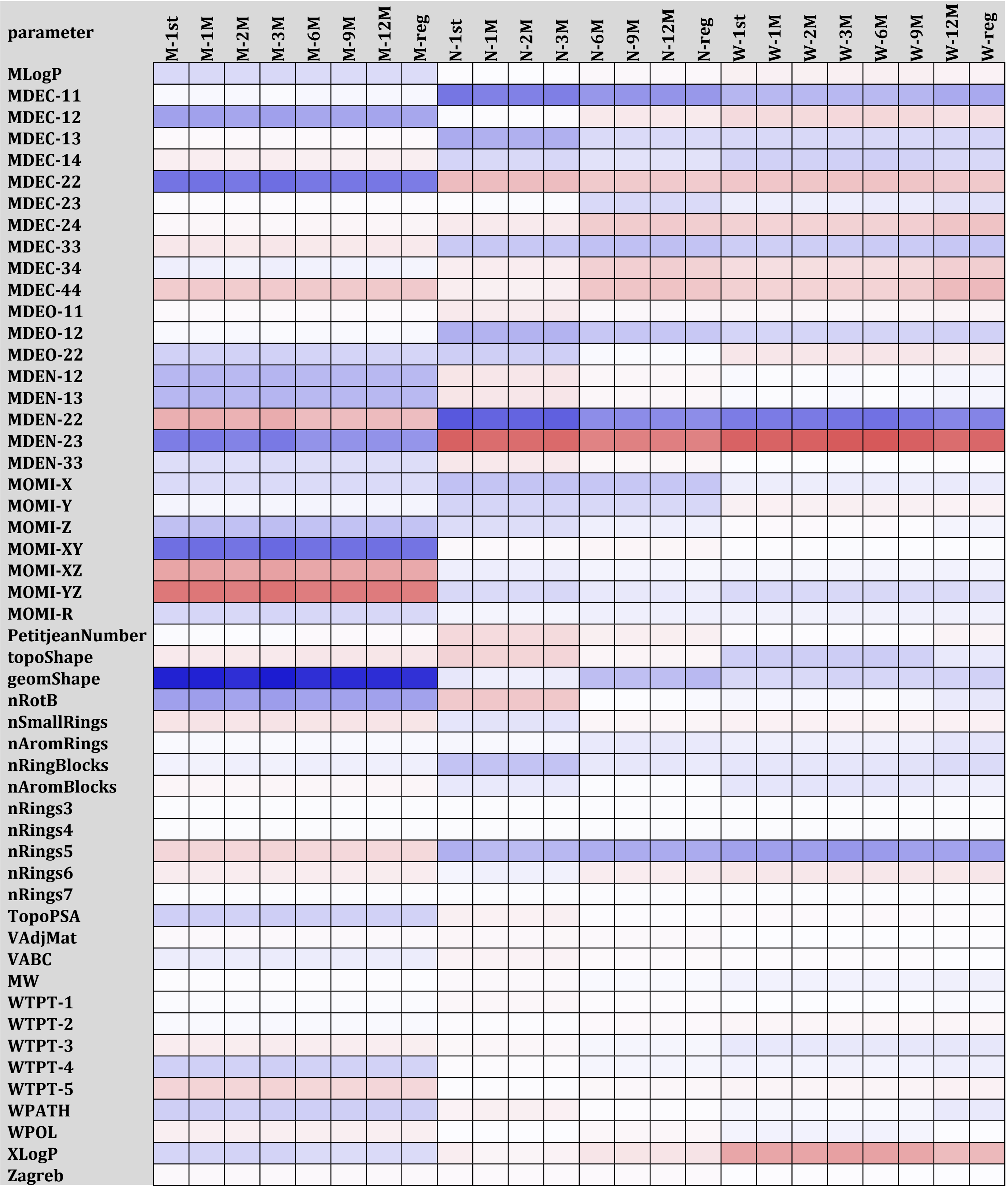

Figure S4. Heatmap of molecular descriptor weights representing their effect on the retention on PGC column as determined by ANN. Blue – decreasing retention, red – increasing retention. M – MeOH as organic modifier, N – MeOH+10 mmol/L NH_3 as organic modifier, W- MeOH+2% H_2O as organic modifier. Data calculated based on the first injection (1st), or after 1 month of use (1M), 2 months (2M), 3 months (3M), 6 months (6M), 9 months (9M), 12 months (12M), or after the regeneration procedure (reg).

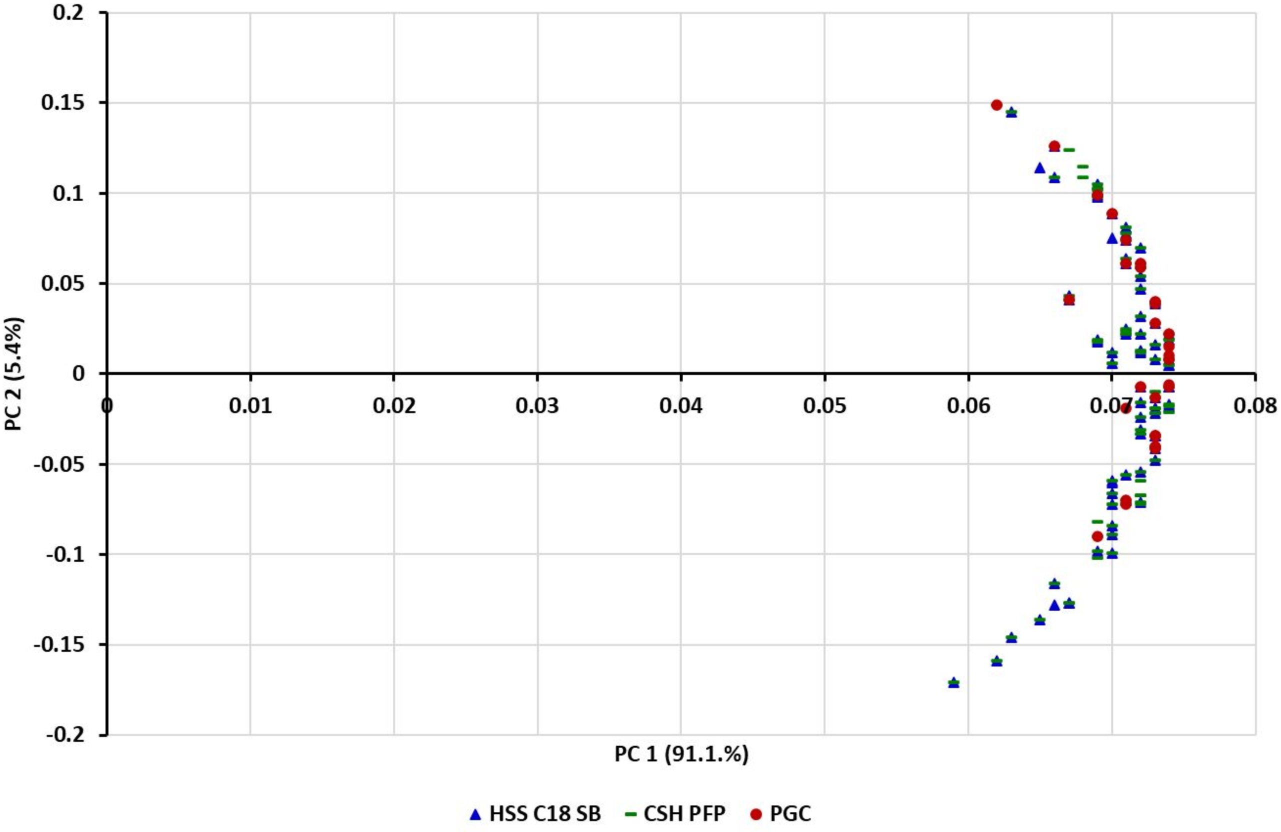

Figure S5. Principal Component Analysis of analytes eluting on each of the tested stationary phase, i.e., HSS C18 SB (blue), CSH PFP (green), PGC (red) based on their molecular descriptors.

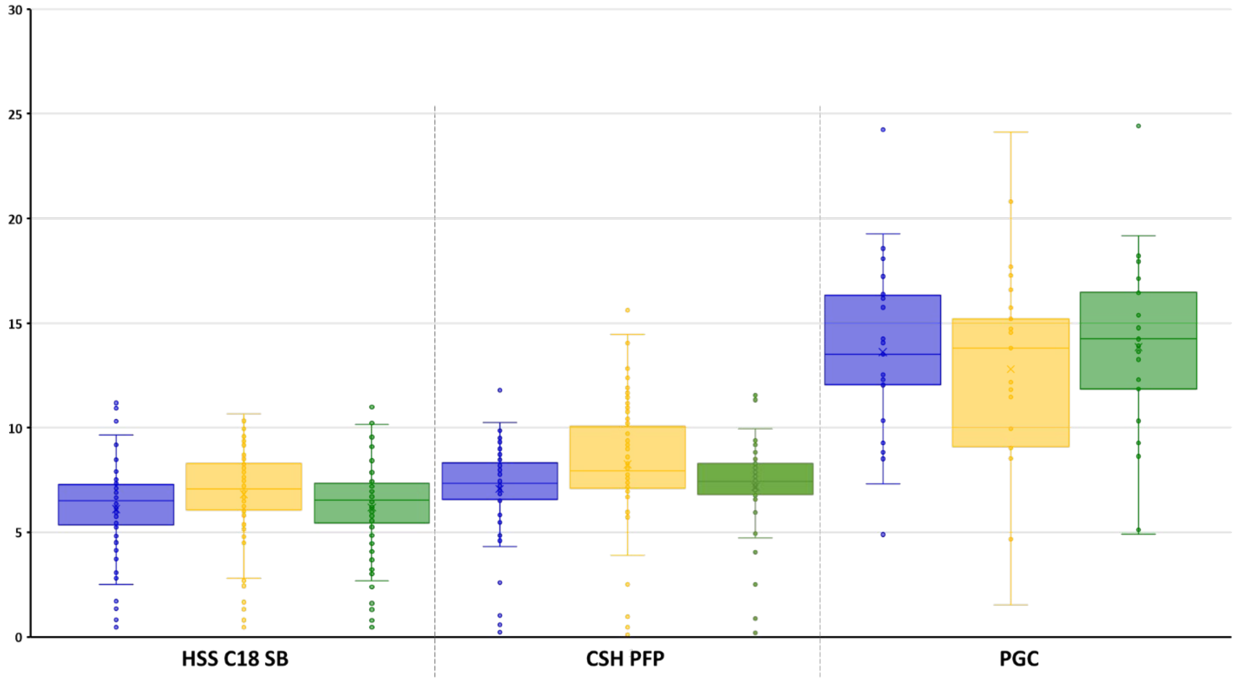

Figure S6. Boxplots of retention factors of compounds eluting on each stationary phase using methanol (blue), 10 mmol/L ammonia in methanol (yellow), and 2% water in methanol (green) as organic modifier.

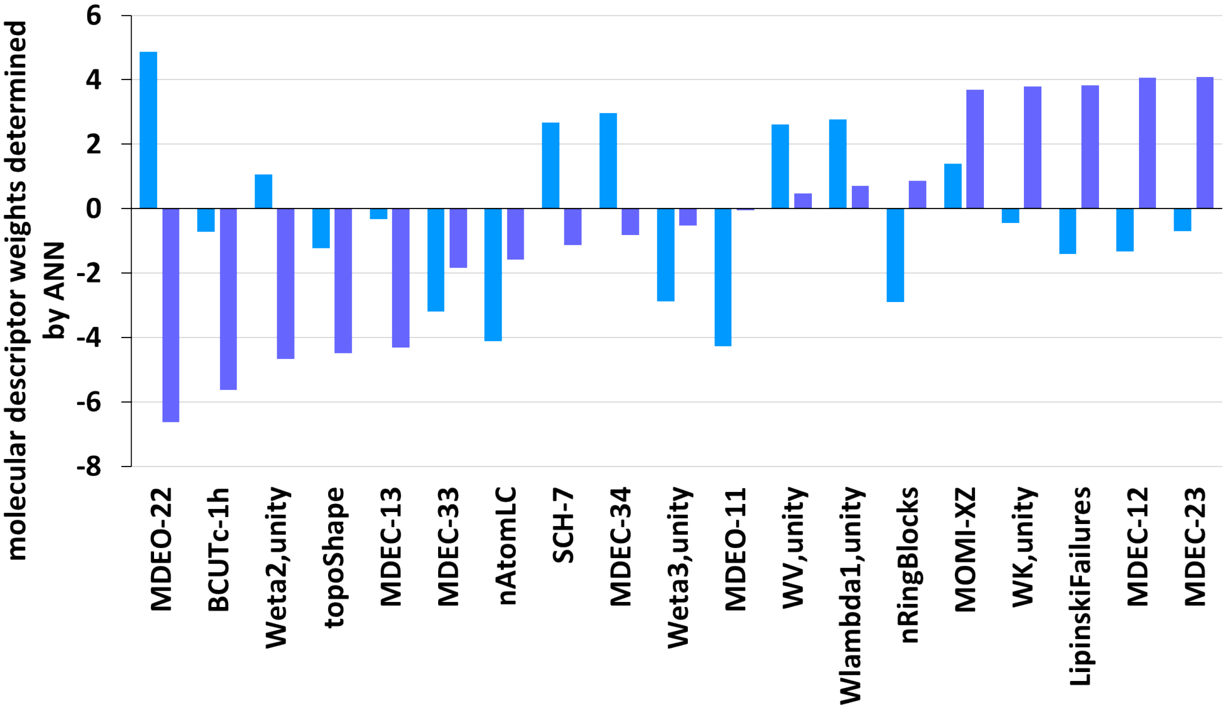

Figure S7. Comparison of molecular descriptors with the highest weight differences assigned by ANN to HSS C18 SB (blue) and CSH PFP (purple) stationary phase, using MeOH as organic modifier and the common set of 50 analytes (Table S1).

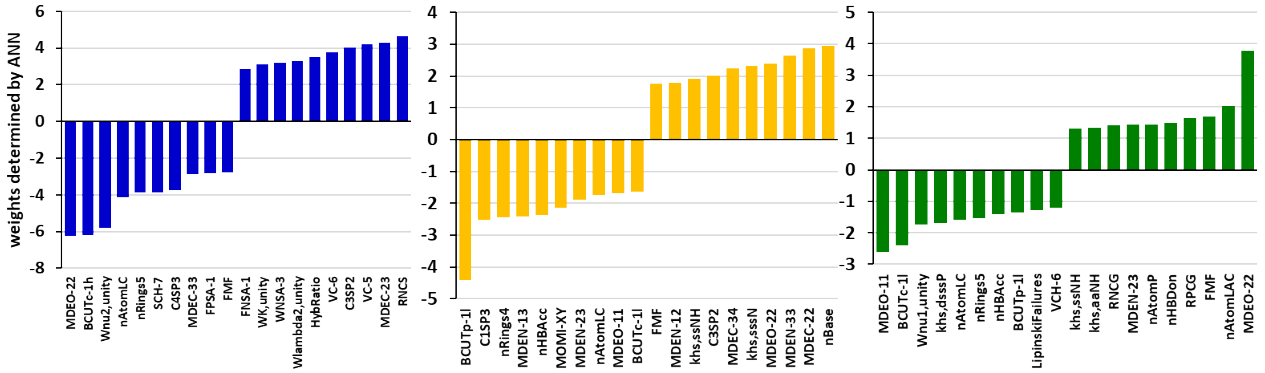

Figure S8. Comparison of weights of key molecular descriptors affecting retention on HSS C18 SB based on used organic modifier. MeOH (blue), MeOH+10 mmol/L NH_3 (yellow), and MeOH + 2% H_2O (green) as organic modifier, with a set of 57 eluted analytes (Table S1).

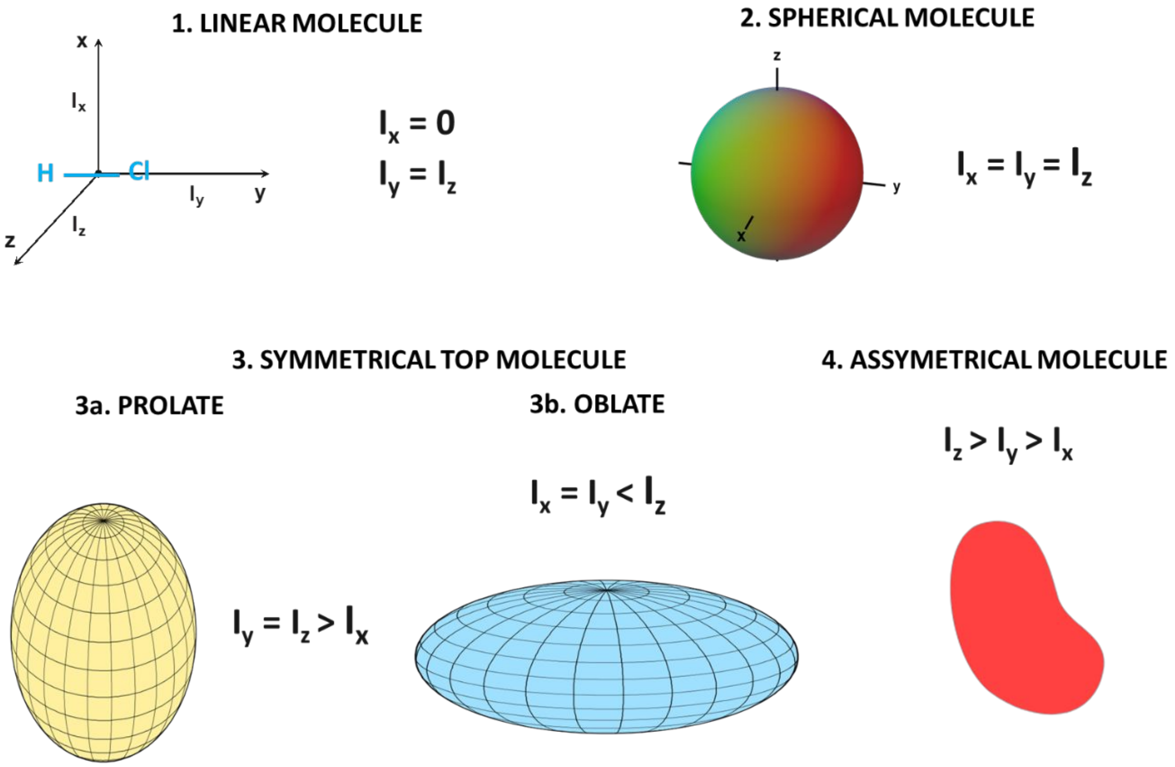

Figure S9. Description and general representation of the moment of inertia (MOMI) molecular descriptor.

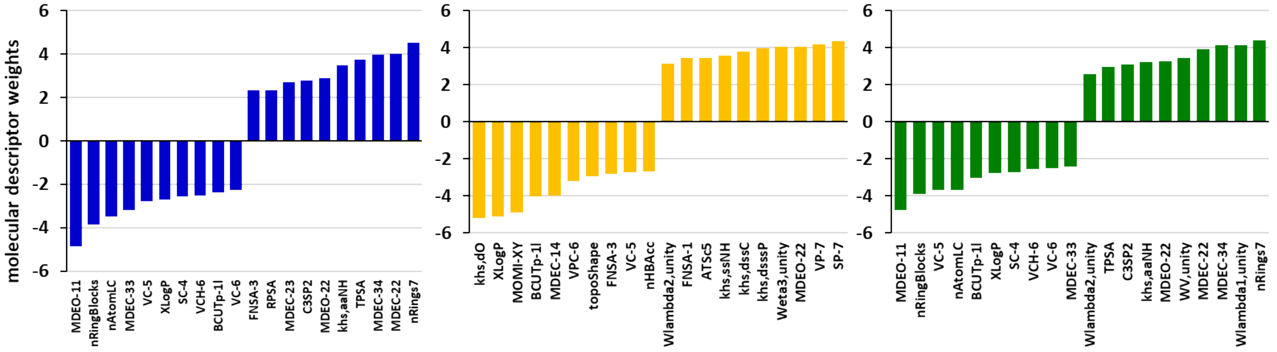

Figure S10. Comparison of the weights of key molecular descriptors affecting retention on CSH PFP based on organic modifier used. MeOH (blue), MeOH+10 mmol/L NH_3 (yellow), and MeOH + 2% H_2O (green) as organic modifier, with a set of 68 eluted analytes (Table S1).

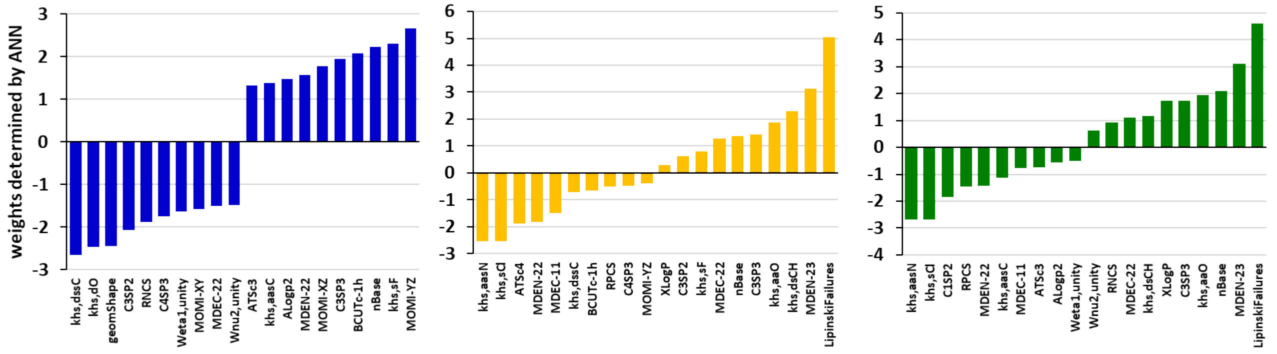

Figure S11. Comparison of weights of key molecular descriptors affecting retention on PGC based on organic modifier used. MeOH (blue), MeOH+10 mmol/L NH_3 (yellow), and MeOH + 2% H_2O (green) as organic modifier, with a set of 28 eluted analytes (Table S1).

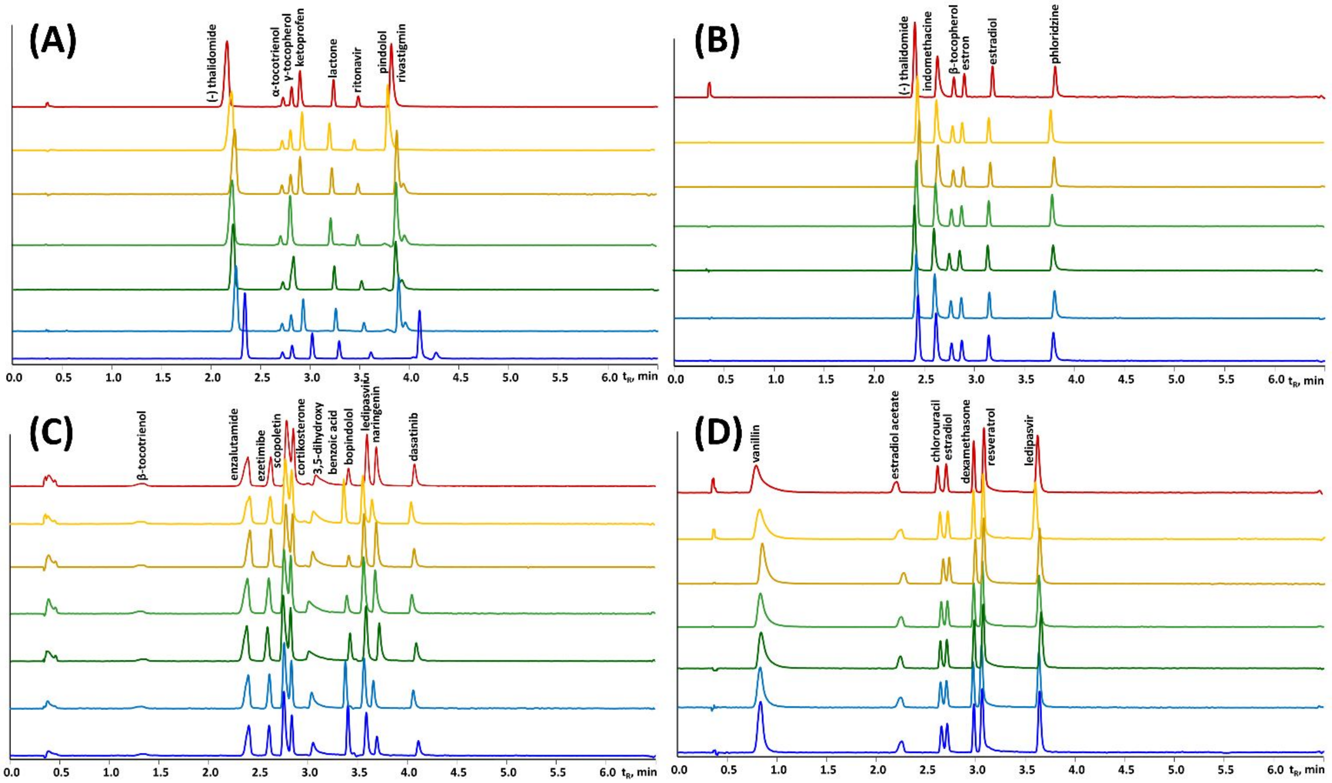

Figure S12. SFC-UV chromatograms of selected analytes collected at different data points using (A) HSS C18 SB/MeOH+10 mmol/L ammonia, (B) HSS C18 SB/MeOH+2% water, (C) CSH PFP/MeOH+10 mmol/L ammonia, (D) CSH PFP/MeOH+2% water. 1st injection (dark blue), month 1 (light blue), month 2 (dark green), month 3 (light green), month 6 (dark yellow), month 9 (yellow), and month 12 (red).

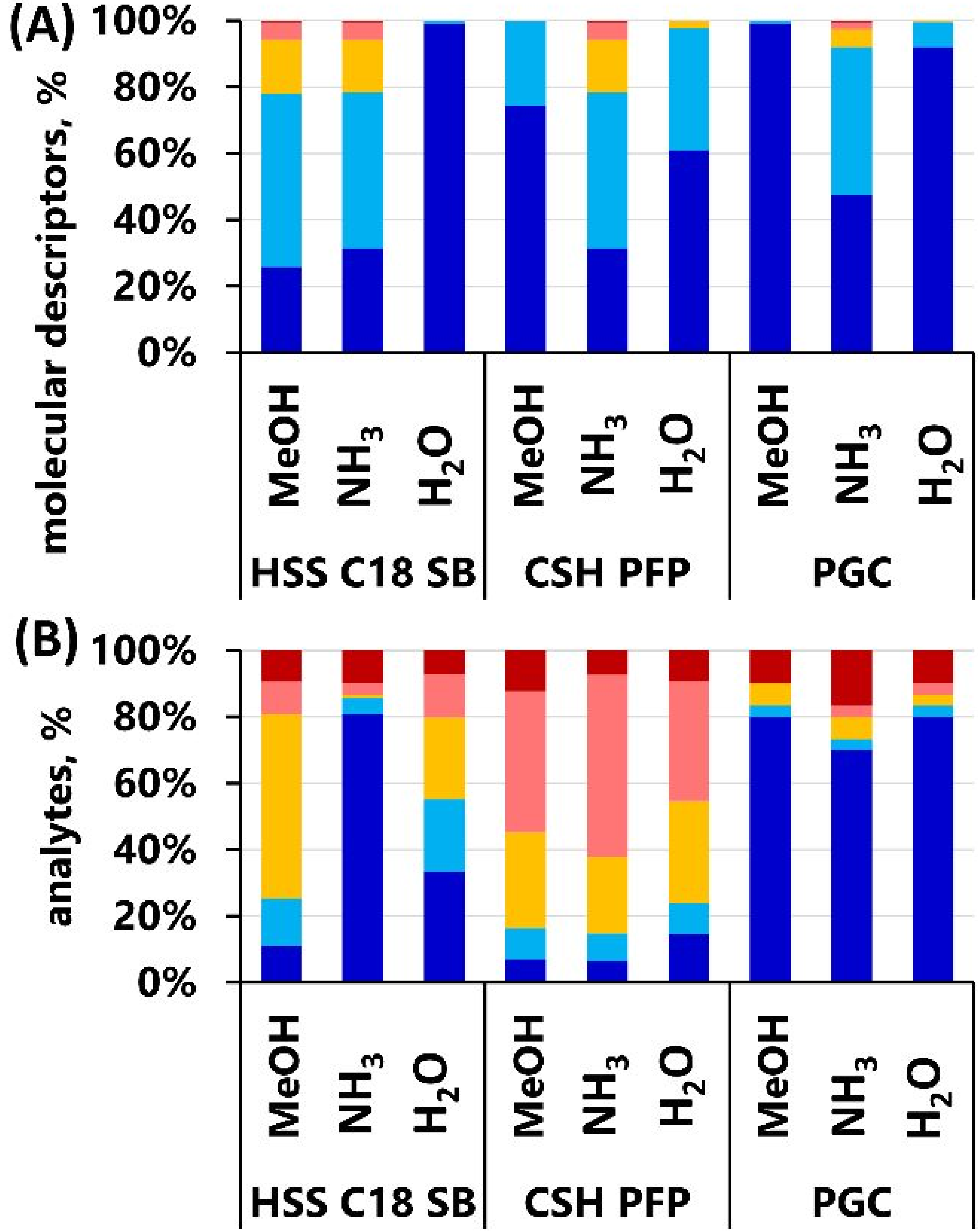

Figure S13. (A) Percentage of molecular descriptors with standard deviations between weights determined by ANN at each data point: < 0.1 (dark blue), 0.1-0.3 (light blue), 0.3-0.5 (yellow), 0.5-1.0 (light red), > 1.0 (dark red). (B) Percentage of compounds with % error between t_R at the 1st injection and after regeneration procedure: < 0.5 (dark blue), 0.5-1.0 (light blue), 1.0-2.0 (yellow), 2.0-5.0 (light red), > 5.0 (dark red).

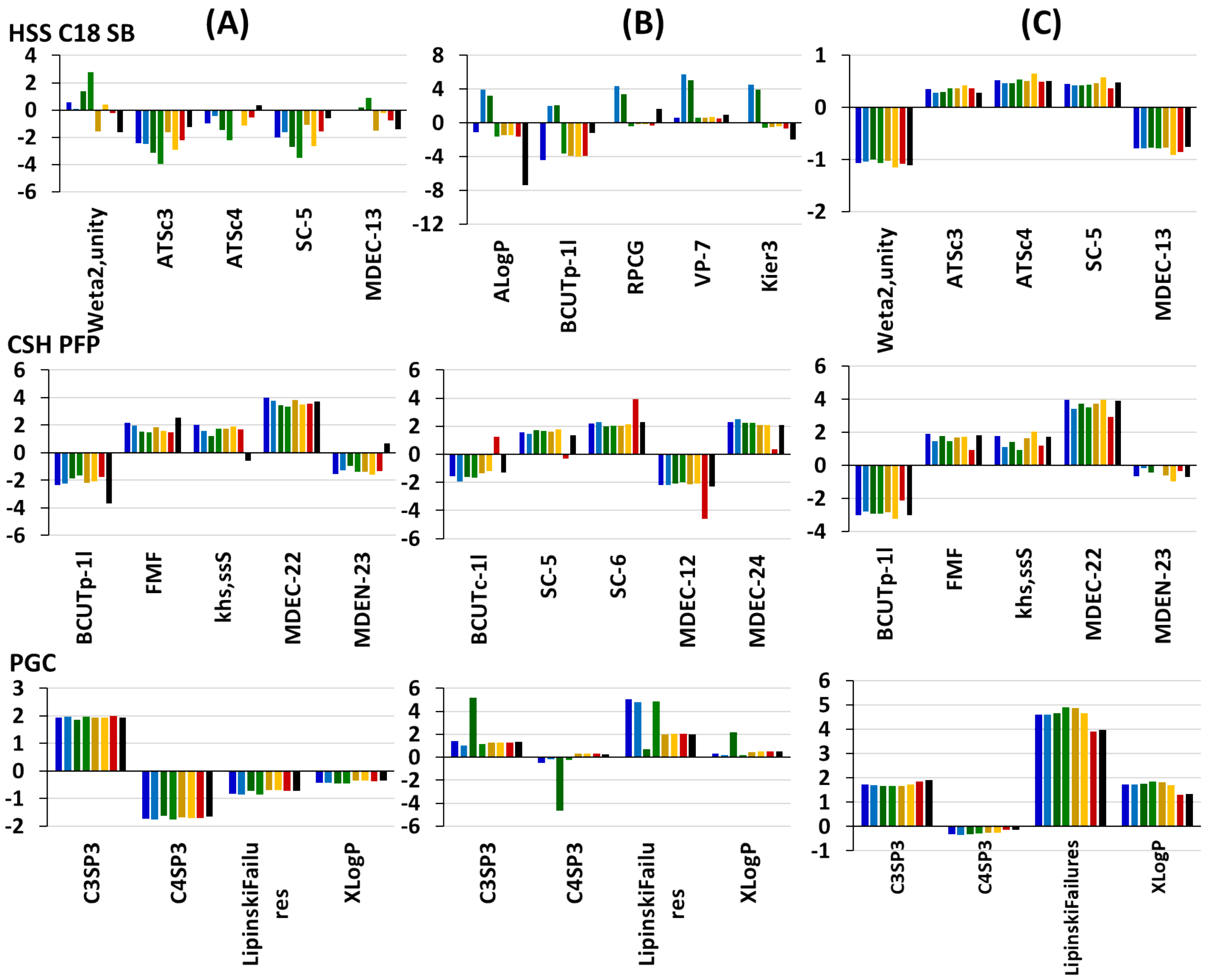

(A)
(B)
(C)
HSS C18 SB
CSH PFP
PGC
Weta2,unity
ATSc3
ATSc4
SC-5
MDEC-13
ALogP
BCUTp-1l
RPCG
VP-7
Kier3
FMF
khs,ssS
MDEC-22
MDEN-23
BCUTc-1l
SC-6
MDEC-12
MDEC-24
C3SP3
C4SP3
LipinskiFailures
XLogP

Figure S14. The molecular descriptors with the weights with the largest changes over time on HSS C18 SB, CSH PFP, and PGC columns using (A) MeOH, (B) MeOH + 10mmol/L ammonia, (C) MeOH + 2% H_2O. Weights determined by ANN based on analysis at 1st injection (dark blue), month 1 (light blue), month 2 (dark green), month 3 (light green), month 6 (dark yellow), month 9 (yellow), and month 12 (red).

(1) West, C.; Lemasson, E.; Bertin, S.; Hennig, P.; Lesellier, E. *Journal of Chromatography A* **2016**, *1440*, 212-228, DOI: https://doi.org/10.1016/j.chroma.2016.02.052
(2) Gros, Q.; Molineau, J.; Noireau, A.; Duval, J.; Bamba, T.; Lesellier, E.; West, C. *J Chromatogr A* **2021**, *1639*, 461923, DOI: 10.1016/j.chroma.2021.461923
